# First chemoenzymatic stereodivergent synthesis of both enantiomers of promethazine and ethopropazine

**DOI:** 10.3762/bjoc.10.322

**Published:** 2014-12-18

**Authors:** Paweł Borowiecki, Daniel Paprocki, Maciej Dranka

**Affiliations:** 1 Warsaw University of Technology, Faculty of Chemistry, Noakowskiego St. 3, 00-664 Warsaw, Poland

**Keywords:** ethopropazine, lipase-catalyzed kinetic resolution, Mosher methodology, promethazine, stereodivergent synthesis

## Abstract

Enantioenriched promethazine and ethopropazine were synthesized through a simple and straightforward four-step chemoenzymatic route. The central chiral building block, 1-(10*H*-phenothiazin-10-yl)propan-2-ol, was obtained via a lipase-mediated kinetic resolution protocol, which furnished both enantiomeric forms, with superb enantioselectivity (up to *E* = 844), from the racemate. Novozym 435 and Lipozyme TL IM have been found as ideal biocatalysts for preparation of highly enantioenriched phenothiazolic alcohols (up to >99% ee), which absolute configurations were assigned by Mosher’s methodology and unambiguously confirmed by XRD analysis. Thus obtained key-intermediates were further transformed into bromide derivatives by means of PBr_3_, and subsequently reacted with appropriate amine providing desired pharmacologically valuable (*R*)- and (*S*)-stereoisomers of title drugs in an ee range of 84–98%, respectively. The modular amination procedure is based on a solvent-dependent stereodivergent transformation of the bromo derivative, which conducted in toluene gives mainly the product of single inversion, whereas carried out in methanol it provides exclusively the product of net retention. Enantiomeric excess of optically active promethazine and ethopropazine were established by HPLC measurements with chiral columns.

## Introduction

Since enantiomorphs of biologically active compounds exhibit significant differences in their pharmacokinetic and pharmacodynamic behavior, optical purity of chiral drugs has emerged as an important factor in efficacy and safety of their application [1–3]. Thus, the development of an effective, inexpensive and environmentally friendly method for the formulation of single enantiomers still remains a challenging task for organic chemists. Nowadays, classical chemical manufacturing technologies are increasingly displaced by pure biotechnological processes or biotransformations coupled with chemical operations [4–10]. The main advantages of these impactful techniques are high substrate specificity as well as regio- and stereoselectivity of the reaction [11–13]. In addition, the easy handling (not involving the use of a complex chemical apparatus), possibility of catalyst recovery, mild experimental (room temperature, atmospheric pressure, etc.) and non-hazardous environmental conditions as well as organic solvent-, moisture- and air-stability make tandem chemoenzymatic processes attractive for both: laboratory and industrial applications. Since enzyme-catalyzed processes provide an access to enantiomerically enriched compounds, they became an extremely practical tool for chemistry and biotechnology [14–21]. A vast number of publications have recently shown the enormous potential of enzymatic bioconversions in the synthesis of high value-added active ingredients and useful chiral building blocks for pharmaceutical or fine-chemical industries [13,22–31]. Among the enzymes applied, lipases occupy a prominent place in advanced organic synthesis as biocatalysts of chiral recognition [32–39].

Nowadays, nervous and mental health problems mainly caused by the pace of life and the rapid development of civilization are very prevalent and dangerous diseases. An irregular and unsanitary lifestyle adversely affects the central nervous system resulting in neuroses and psychiatric disorders. Among the antipsychotic medications phenothiazine derivatives [40–41], and especially promethazine, widely known by its brand name Phenergan^®^, hold well deserved place. As it posses excellent antihistaminic activity (H_1_-antagonist) [42], pronounced antimuscarinic [43], anticholinergic [44], sedative, antiemetic and adrenergic blocking action as well as some local anesthetic properties [45], promethazine is approved for the effective therapy of various disorders clearly diverging from the original application. It has gained widespread usage in the treatment of allergic reactions, itching dermatitis, cough (when applied together with codeine or dextromethorphan), sleeplessness or symptoms of motion, morning and Ménière's sicknesses including nausea and vomiting. For over four decades it has been considered that both enantiomers of promethazine posses exactly the same therapeutic activities [46–47], and thus it was administered in clinical settings as racemate. However, recent findings have shown that the different physiological and pharmacological effects of the two enantiomers of promethazine are a fact, what makes this drug returned to the laboratories. For example, it has been determined that (+)-promethazine reduced the cytokine IL-6 production in histamine-stimulated cells to 90% while (−)-promethazine induced only 50% reduction in cytokine IL-6 production [48]. Moreover, Boland and McDonought [49–50] found that preferably the (+)-enantiomer of promethazine is particularly effective in inhibiting the formation of bone resorbing cells (osteoclasts) thus providing a new class of agents useful for preventing or even treating bone loss, mainly associated with periodontitis and osteoporosis. Moreover, kinetic resolution of the enantiomers of the key intermediate 1-(10*H*-phenothiazin-10-yl)propan-2-ol may simultaneously provide access to another valuable compound in enantioenriched form, that is: ethopropazine (profenamine). In turn, this antidyskinetic drug has been widely used in clinical practice for over the past 30 years in the treatment of Parkinson's disease, although there is no data available regarding its pharmacokinetic properties in humans. Only one study on the mechanism of stereoselective interaction between butyrylcholinesterase (BChE) and ethopropazine enantiomers was performed leading to the final conclusion that BChE possesses a significantly higher affinity for the (*R*)-configured ethopropazine [51]. In order to find whether ethopropazine displays stereoselectivity in its pharmacokinetics and exhibits different biological profiles of its stereoisomers, obtaining both enantiomers in more than analytical quantities is particularly desirable. Moreover, the racemic mixture of this pharmaceutical is commercially available, however it is sold at high price, as its production by standard synthetic procedures is time-consuming and laborious. Therefore, a low-cost, practical, and scalable method for the straightforward preparation of ethopropazine is still a great challenge.

To the best of our knowledge, heretofore no reports concerning the enzymatic preparative-scale synthesis of optically active promethazine and ethopropazine have been reported. Only enantioselective analytical methods towards enantioresolution of promethazine employing various chiral selectors including proteins, cyclodextrines, modified crown ethers or macrocyclic antibiotics have been proposed [52–56]. Other two reports [57–58] containing information about ethopropazine enantiomers separation (mainly via fractional crystallization or column chromatography splitting of diastereomeric dibenzoyltartaric acid salts) have been described. Despite the above-mentioned results, the availability of a scalable stereoselective procedure has, as far as we know, been deemed insufficient. We believe that the lipase-catalyzed resolution procedure presented herein, will be an interesting alternative to these published methods, and will open a novel route worth considering in multigram-scale synthesis of both aforementioned pharmaceuticals.

Therefore, the aim of this study was to take advantage of the extraordinary properties of enzyme catalysis and develop a new method based on lipase-mediated kinetic resolution (KR) of 1-(10*H*-phenothiazin-10-yl)propan-2-ol enantiomers, which combined with convenient chemical reactions could provide a simple and straightforward approach for the preparation of optically active promethazine and ethopropazine molecules, respectively.

## Results and Discussion

**Scheme 1:**
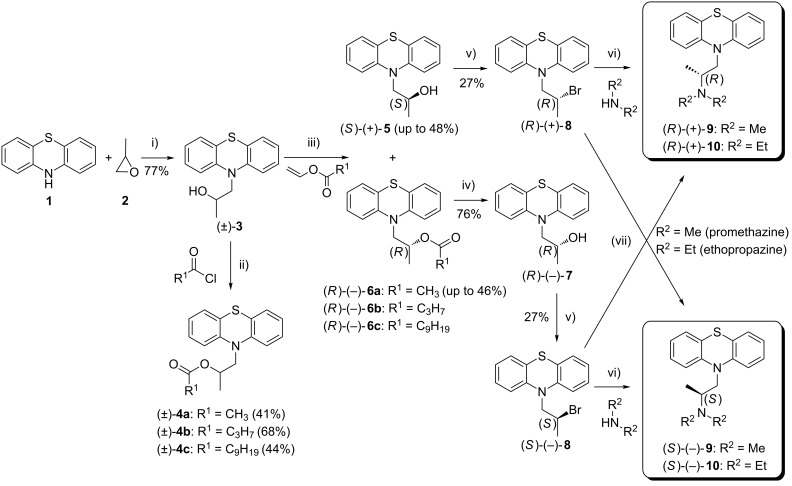
Chemoenzymatic synthesis of enantioenriched enantiomers of promethazine **9** and ethopropazine **10**. Reagents and conditions: (i) *n*-BuLi (1.5 equiv), dry THF, 1 h at −78 °C, then propylene oxide **2** (2 equiv), 12 h at rt; (ii) CH_3_COCl (1.5 equiv) or C_3_H_7_COCl (1.5 equiv) or C_9_H_19_COCl (1.5 equiv), NEt_3_ (1.5 equiv), DMAP (0.1 equiv), dry CH_2_Cl_2_, 12 h at rt; (iii) vinyl ester (3 equiv), lipase [20% (w/w)], MTBE, 25 °C, 500 rpm (magnetic stirrer); (iv) NaOH (1.1 equiv), MeOH, 1 h at rt; (v) PBr_3_ (1 equiv), CH_2_Cl_2_, 2 h at rt; (vi) Me_2_NH (5 equiv) or Et_2_NH (20 equiv), MeOH, sealed tube, 4 d at 90 °C (temperature of oil bath); (vii) Me_2_NH (25 equiv) or Et_2_NH (50 equiv), PhCH_3_, sealed tube, 7 d at 140 °C (temperature of oil bath).

Herein, we wish to present an original chemoenzymatic procedure for the enantioselective synthesis of promethazine **9** and ethopropazine **10**. Most of our efforts during these studies have been focused toward extensive screening of the conditions for the lipase-catalyzed kinetic resolution of 1-(10*H*-phenothiazin-10-yl)propan-2-ol racemate (±)-**3**. The other part of the work involved investigation of the enzymatic reactions stereoselectivity, which was accomplished by means of the assignment of the absolute configuration of the resolved enantiopure alcohol (*S*)-(+)-**5** determined by a modified Mosher’s methodology, and was unambiguously confirmed by X-ray diffraction analysis. Optically active intermediates (*S*)-(+)-**5** and (*R*)-(−)-**7** achieved in this manner were subsequently transformed into active pharmaceuticals **9** and **10** as (*R*)- and (*S*)-enantiomers afforded in high enantiopurity (84–98% ee) (Scheme 1) and in a stereodivergent fashion.

### Synthesis of the racemic 1-(10*H*-phenothiazin-10-yl)propan-2-ol (±)-**3** and its acyl esters (±)-**4a–c**

In the first step, racemic 1-(10*H*-phenothiazin-10-yl)propan-2-ol (±)-**3** was synthesized according to the method described by Clement et al. [59], in which propylene oxide (**2**) was regioselectively opened by phenothiazine (**1**) in the presence of *n*-butyllithium (*n*-BuLi) at ambient temperature providing desired alcohol (±)-**3** in 64–77% yield depending on the applied scale and the purification method (see Supporting Information File 1). Paradoxically, the product was obtained in higher yield when the reaction was performed on a bigger scale, and when the product was isolated by vacuum distillation instead of column chromatography (SiO_2_). It is worth to note, that the preparation of (±)-**3** was very problematic. Before we decided to repeat the reaction procedure proposed by Clement, we attempted to obtain (±)-**3** by adopting the original methodology reported by Dahlbom [60]. Unfortunately, quite surprisingly the reaction conducted by this approach, which assumed usage of sodium amide (NaNH_2_) as a base, produced enormous amounts of byproducts, which significantly hampered both the isolation and purification procedures. The same phenomenon was observed if sodium hydride (NaH) was utilized instead of NaNH_2_ under similar reaction conditions, what suggests that this particular process is most probably inaccessible through the sodium salt of phenothiazine (**1**) due to multiple substitution at aromatic ring carbon atoms and some other byproducts of the non-regioselective propylene oxide ring opening. With the hope of eliminating the unwanted side-product formation, we thereby performed the reaction under phase transfer catalysis (PTC) conditions. The results of this experiments have shown that independently from the type of the used reaction media (toluene, CH_2_Cl_2_, diethyl ether), bases (50% NaOH or 60% KOH), and the applied PTC-catalysts [tetrabutylammonium bromide (TBAB) or tetrabutylammonium hydrogensulfate (TBAHS)], the epoxide **2** ring opening proceeded unsuccessfully. Again, the amount of the formed impurities was too large to isolate pure fraction. This provoked us to change the synthetic strategy by excluding at first propylene oxide (**2**) as the reagent. Reported in the literature various PTC-mediated alkylations of phenothiazine (**1**) with various alkyl and alkenyl halides [61–64], inspired us to prepare the 10-(prop-2-en-1-yl)-10*H*-phenothiazine derivative as potent substrate for the synthesis of (±)-**3**. This was smoothly achieved in high yield (76%) by using allyl bromide in biphasic system composed of CH_2_Cl_2_/50% NaOH and the additive TBAHS as the catalyst at room temperature. The obtained 10-(prop-2-en-1-yl)-10*H*-phenothiazine was subsequently conducted into an oxymercuration–demercuration reaction accordingly to the procedure proposed by Abreu et al. [65]. Although the recorded mass spectra (HRMS–ESI) for the thus prepared product (±)-**3** showed the quasi-molecular peak corresponding to the calculated value, and both the gas chromatography (GC) as well as thin layer chromatography (TLC) showed a single peak and spot respectively, the signals of the measured ^1^H NMR spectra were strongly diffused and could not be easily interpreted. This can be explained by the presence of traces of diamagnetic impurities (Hg^2+^-complex) in the sample of (±)-**3**, which caused severe broadening of the signals due to reduction of relaxation times. This prevents (±)-**3** to be accomplished in the required purity for pharmaceuticals production. All other attempts performed by us, which concerned *N*-alkylation of **1** with various alkyl agents such as: 1-chloropropan-2-ol, (1-chloropropan-2-yloxy)trimethylsilane or chloroacetone under PTC or NaH-base conditions, failed as well leading to complex mixtures. The above mentioned drawbacks were finally overcome by using the lithium salt of phenothiazine **1** for the oxirane **2** ring opening as it was described at the beginning of this paragraph.

To obtain racemic esters (±)-**4a**–**c** which are required for robust analytical HPLC separation studies of the corresponding pairs of enantiomers and for the measurement of the enantiomeric excess values of the compounds prepared in the biocatalyzed reactions, the afore-prepared alcohol (±)-**3** was reacted with the appropriate acyl chloride in dry dichloromethane in the presence of triethylamine and a catalytic amount of 4-*N*,*N*-dimethylaminopyridine (DMAP). This was leading to moderate yields (41–68%) of the acetates.

### Lipase-catalyzed kinetic resolution of (±)-**3**

To address the challenges associated with the determination of the best reaction conditions for the asymmetric chemoenzymatic total synthesis of promethazine (**9**) and ethopropazine (**10**), necessary optimization studies of the lipase-catalyzed kinetic resolution of (±)-**3** were undertaken. For that purpose, we considered the influence of major factors including: (i) type of the lipase, (ii) choice of the co-solvent, (iii) reaction time, and (iv) kind of acyl donor on the yield and enantioselectivity outcome of the overall process. For preliminary analysis, we followed the strategy of keeping all the other experimental parameters constant, i.e., stirring speed (500 rpm), enzyme loading in respect to substrate (20% w/w), substrate-to-acyl group donor molar ratio (1:30), and the temperature (25 °C). Finally, after finding optimal biotransformation conditions in a mg-scale, we have successfully performed the reaction in gram- and multigram-scales providing sufficient amounts of optically active alcohol intermediates (*S*)-(+)-**5** and (*R*)-(−)-**7** enabling to continue the planned synthesis. Each of the reaction parameters and up-scaling approaches are discussed in detail in the following paragraphs.

### Biocatalyst effect on the kinetic resolution of (±)-**3**

The enzymatic kinetic resolution of (±)-**3** was initially attempted using a representative set of 14 different lipases isolated from various microorganisms (see Supporting Information File 1 for details). Among the broad panel of investigated commercially available preparations, immobilized lipases of a fungal origin such as Novozym 435, Chirazyme L-2, C2, Chirazyme L-2, C3, and Lipozyme TL IM were established as optimal biocatalysts for the enantioselective transesterification when suspended in 30 equiv of vinyl acetate as the acyl donor, methyl *tert*-butyl ether (MTBE) as co-solvent, and carried out at 25 °C, with agitation speed of the magnetic stirrer arranged at 500 rpm, and an enzyme loading of 20% w/w in respect to substrate (±)-**3**.

**Figure 1:**
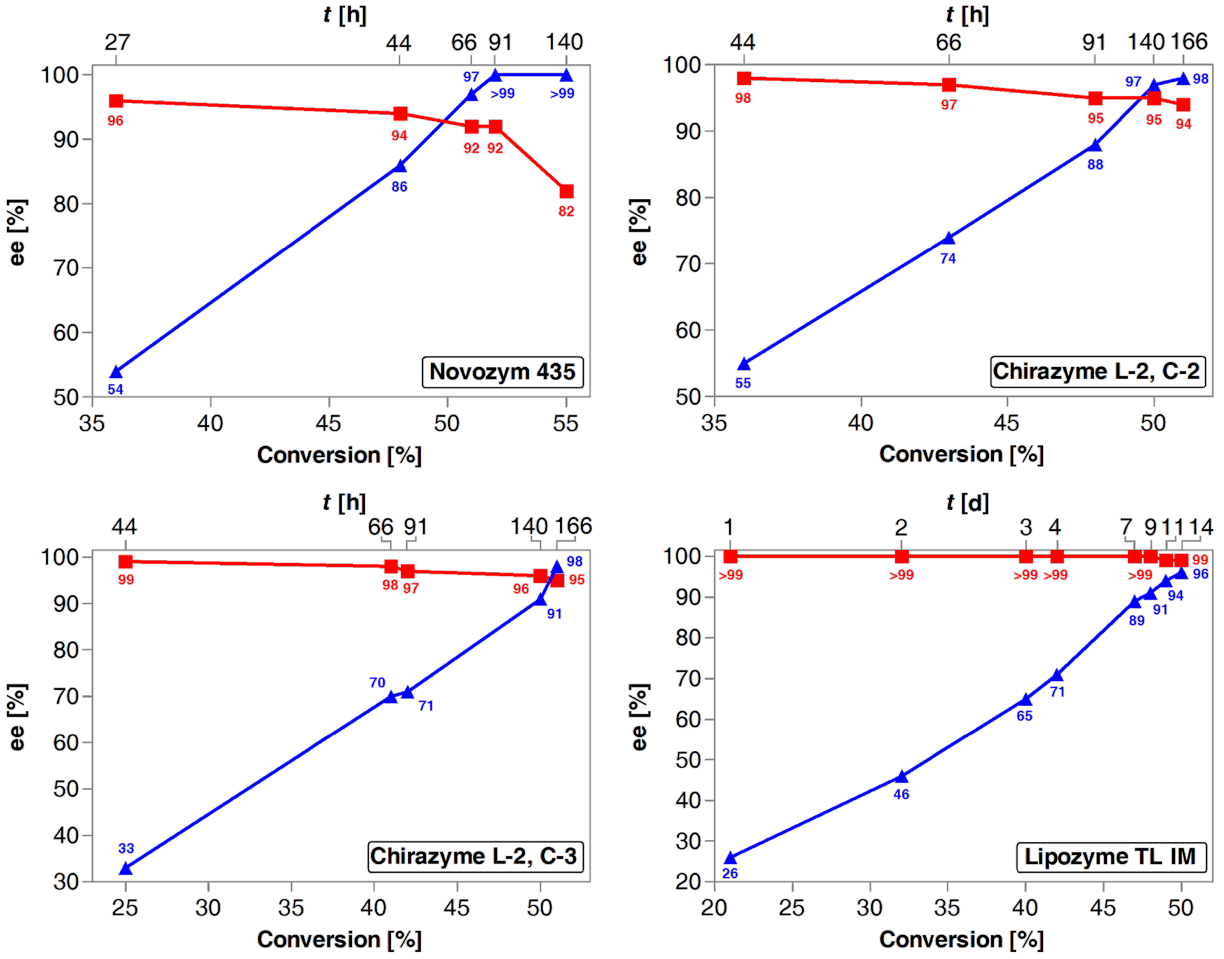
Dependence of optical purities (% ee) of (*R*)-(−)-**6a** (red curve, ■) and (*S*)-(+)-**5** (blue curve, ▲) on the conversion degree of (±)-**3** during lipase-catalyzed acetylation with vinyl acetate in a MTBE solution at 25 °C (magnetic stirrer, at 500 rpm).

Whereas preliminary experiments showed that four of the above-mentioned lipase preparations exhibited good enzymatic activities and excellent enantioselectivities towards racemic alcohol (±)-**3**, it would have been insufficient to select the most promising catalyst only on the basis of single random results obtained from the analysis of the reaction mixtures. By virtue of the importance of enzyme selection, and to find the most suitable catalyst for the transesterification of racemic alcohol (±)-**3** as well as to provide better insight into the reaction progress of each of the chosen lipases in general, comprehensive kinetic studies were assessed (Figure 1). Effect of the reaction time on the conversion degree of phenothiazinic alcohol (±)-**3** and the overall progress of the followed enzymatic process was estimated by high-performance liquid chromatography (HPLC) analysis using a Chiralcel OD-H column. The products could be analyzed directly from the crude mixture since the conditions of the HPLC chiral stationary phases were adjusted to the needs of both resolution products (peaks of the alcohol and the acetate enantiomers were well separated) (see the Supporting Information File 1 for details). The plots presented in Figure 1 clearly indicate that in terms of enantioselectivity, the best results were obtained with Lipozyme TL IM with an enantiomeric ratio factor (*E*) reaching up to 790 for 49% conversion achieved after 14 days, whereas other lipase preparations generally displayed lower enantioselectivities (with *E*-values up to 180).

While tracing the course of the Lipozyme TL IM-catalyzed reaction it became evident that in most cases substrate (±)-**3** was slowly but ultra-selectively converted into the corresponding acetate (*R*)-(−)-**6a** obtained with excellent enantiomeric excess (>99% ee), while the remaining alcohol (*S*)-(+)-**5** was recovered only with 96% ee due to the fact that the reaction definitely stopped at 49% conversion being reached after two weeks. The specificity of this lipase for the (*R*)-alcohol was evident since even after such a long reaction time, no trace of the (*S*)-configured ester was detected. As occurred at elevated temperature (35 °C), the reaction did not proceed further with longer reaction times. Nevertheless, the results obtained with this lipase were truly impressive, as all the investigated samples revealed that generated ester (*R*)-(−)-**6a** was enantiomerically pure (>99% ee) regardless of the time and the conversion.

In turn, the reaction rate was considerably improved in Novozym 435-catalyzed analytical scale acetylation. This revealed that *Candida antarctica* lipase B (CAL-B) immobilized on acrylic resin was superior to other enzymes in both the reaction rate and the obtained enantiomeric excess values of the slower reacting enantiomer (*S*)-(+)-**5**. In this case, racemic alcohol (±)-**3** has been successfully resolved affording the unreacted substrate (*S*)-(+)-**5** in enantiopure form (>99% ee), whereas the ester (*R*)-(−)-**6a** was yielded in high enantiomeric excess (82–92% ee) when the reaction slightly exceeded 52% conversion. As can be seen in Figure 1, the time necessary to achieve the appropriate 51% conversion for Novozym 435-catalyzed reaction was diminished by 2.5 orders-of-magnitude when compared to both Chirazyme-type preparations. Even more significant (by 5 orders-of-magnitude) difference in the speed of the reactions was observed when comparing the time necessary for ca. 48% conversion achievement in the case of Novozym 435 and Lipozyme TL IM lipases. On the other hand, Chirazyme L-2, C2 and Chirazyme L-2, C3 displayed very high enantioselectivity (*E* = 149–180) yielding stereoselectively acetylated derivative (*R*)-(−)-**6a** in high enantiomeric excess (94–95% ee) and unreacted alcohol (*S*)-(+)-**5** with 98% ee. Examination of both reaction progresses showed very characteristic trends in kinetics for this type of lipase-catalyzed reaction, where an almost linear increase in the enantiomeric excess during the formation of optically active alcohol (*S*)-(+)-**5** could be observed. However, although good selectivities were attained in the kinetic resolution of (±)-**3** catalyzed by both Chirazyme preparations, the reaction rates were low and 51% conversion was achieved after 166 h hampering the development of a straightforward route to optically active targeted pharmaceuticals **9** and **10**. The afore-outlined results forced us to cease further optimization studies with these particular lipases. The enzyme selection stage leads to the conclusion that reactions catalyzed by Novozym 435 were beneficial for obtaining the alcohol (*S*)-(+)-**5** in enantiopure form, while highly optically pure acetate (*R*)-(−)-**6a** was obtained with Lipozyme TL IM as the catalyst. These findings provoked us to investigate the kinetic resolution of (±)-**3** by using both Novozym 435 and Lipozyme TL IM preparations in the next optimization stages.

### Solvent effect on kinetic resolution of (±)-**3**

The next stage of enzymatic analytical scale studies was designed to find the most suitable co-solvent system for the enantioselective *O*-acylation of racemic alcohol (±)-**3** using vinyl acetate. All experiments of the kinetic resolution of enantiomers (±)-**3** were conducted under the same reaction conditions as described above, using immobilized lipases: Novozym 435 and Lipozyme TL IM as the catalysts, respectively.

It is well-known that the nature of solvent could significantly influence the activity and selectivity of the enzymatic reaction as well as increase the thermal stability of enzymes [66]. The high sensitivity of lipases towards the medium environment mainly stems from experimental observations that different organic solvents had a different ability to interact with amino acid residues which are responsible for the catalytic activity. For example, it is well documented [67–69] that polar solvents (log *P* < 1) are less suitable for biocatalytic purposes since hydrophilic media (such as methanol, ethanol, acetone, acetonitrile etc.) distort the active conformation of the enzyme molecule by destabilizing electrostatic interactions, and even strip off the essential water layer (water of hydration) leading to loss of the biocatalytic activity. In turn, more lipophilic organic solvents positively interact with the enzyme protein enhancing its secondary structure mainly by stabilization of the internal hydrogen-bonding network in the molecule, thus improving the reactivity and selectivity of organic substrate transformations.

Therefore, an appropriate medium selection in which a relatively high solubility of the phenothiazinic substrate (±)-**3** as well as a good enzymatic activity and selectivity are retained, is being of particular significance for the overall success of the process. Five different apolar organic solvents (varying in log *P*, dielectric constant ε, dipolar moment μ) including: *n*-hexane, pentane, toluene, MTBE, and diethyl ether (Et_2_O) were investigated as the reaction media. Additionally, both lipase catalysts were also screened in neat vinyl acetate (Table 1). The courses of all the enzymatic reactions were monitored by gas chromatography (GC), arrested deliberately after 7 days, and only then their final evolution were subsequently followed by chiral HPLC.

**Table 1:** Solvent influence on the selective *O*-acetylation of (±)-**3** in the presence of lipase preparation (Novozym 435 or Lipozyme TL IM) with vinyl acetate in MTBE at 25 °C after 7 days.

Entry	Enzyme^a^	Solvent (log *P*)^b^	conv.^c^ (%)	ee_s_^d^ (%)	ee_p_^d^ (%)	*E*^e^

1	Novozym 435	hexane (3.00)	53	>99	89	90
2		pentane (2.58)	54	>99	84	59
3		toluene (2.52)	51	98	95	180
4		MTBE (0.96)	53	>99	88	82
5		Et_2_O (0.76)	53	>99	88	82
6		vinyl acetate (0.54)	51	97	94	136

7	Lipozyme TL IM	hexane (3.00)	18	22	98	123
8		pentane (2.58)	41	70	>99	419
9		toluene (2.52)	29	40	99	295
10		MTBE (0.96)	44	78	>99	474
11		Et_2_O (0.76)	37	58	>99	359
12		vinyl acetate (0.54)	28	39	99	292

^a^Conditions: (±)-**3** 100 mg, lipase 20 mg, organic solvent 1 mL, vinyl acetate 948 mg (1 mL, 28 equiv), 25 °C, 500 rpm (magnetic stirrer). ^b^Logarithm of the partition coefficient of a given solvent between *n*-octanol and water according to ChemBioDraw Ultra 13.0 software indications. ^c^Based on GC, for confirmation the % conversion was calculated from the enantiomeric excess of the unreacted alcohol (ee_s_) and the product (ee_p_) according to the formula conv. = ee_s_/(ee_s_ + ee_p_). ^d^Determined by chiral HPLC analysis by using a Chiralcel OD-H column. ^e^Calculated according to Chen et al. [70], using the equation: *E* = {ln[(1 – conv.)(1 – ee_s_)]}/{ln[(1 – conv.)(1 + ee_s_)]}.

As a first attempt, Novozym 435-catalyzed KR of racemic alcohol (±)-**3** was carried out (Table 1, entries 1–6). After series of experiments, the influence of various co-solvents could be summarized as the following row orders in terms of enzyme activity: pentane > *n*-hexane ≈ MTBE ≈ Et_2_O > toluene ≈ vinyl acetate, and the reaction enantioselectivity: toluene > vinyl acetate > *n*-hexane > MTBE ≈ Et_2_O > pentane. As it was observed, the acetylation reaction in toluene was mostly enantioselective (*E* = 180), thus transforming the racemic starting material (±)-**3** into optically active ester (*R*)-(−)-**6a** of high enantiomeric excess (95% ee) and leaving thereby slower reacting enantiomer (*S*)-(+)-**5** of very high enantiomeric purity (98% ee). On the other hand, from the view point of remaining the enatiopurity of alcohol (*S*)-(+)-**5**, ethereal solvents (Et_2_O and MTBE) which yielded (*S*)-(+)-**5** with excellent enantiomeric excess (>99% ee) are even better. It is obvious that since methyl *tert*-butyl ether is free from dangerous peroxides, hence it was chosen for further optimization studies concerning Novozym 435 lipase.

In turn, while investigating the acylation of (±)-**3** catalyzed by Lipozyme TL IM suspended in chosen co-solvents systems (Table 1, entries 7–12) we noticed the following row order of the reaction velocity: MTBE > pentane > Et_2_O > toluene > vinyl acetate > *n*-hexane. Quite surprisingly, the same order was observed for the reaction enantioselectivity. As can be seen, among the investigated apolar solvents for Lipozyme TL IM catalyzed kinetic resolution of (±)-**3**, the best results were achieved in MTBE both in terms of enzyme activity and reaction enantioselectivity.

### The effect of the acyl donor on the kinetic resolution of (±)-**3**

A considerable number of fundamental researches have been performed to explain the influence of the acyl group donor type on the lipase-catalyzed acylation of alcohols. Amongst the wide range of this reagent type, enol esters (i.e., vinyl acetate, isopropenyl acetate), activated esters (i.e., trifluoroethyl butyrate, *S*-ethyl thiooctanoate), non-activated esters (i.e., ethyl acetate, methyl acetate), and anhydrides (i.e., acetic acid anhydride, succinic acid anhydride) are most commonly used. Unfortunately, there are several drawbacks of incorporating the above-mentioned reagents into transesterification processes, i.e., trifluoroethyl esters are very expensive, *S*-ethyl thioesters generate undesirable flavor of the formed thiols, employment of anhydrides yields free carboxylic acids causing enzyme deactivation, and finally application of conventional esters, despite their great availability and low price, could reverse acylation into alcoholysis due to alcohol formation during the course of reaction. Of course, removal of alcohol from the reaction system by, i.e., 4 Å molecular sieves could prevent lipase-catalyzed alcoholysis of the formed ester and thus shift the reaction toward the product. However, a straightforward method to avoid the formation of alcohol molecules is available, and assumes the use of vinyl esters as the acyl group donors. Moreover, from the application point of view, vinyl esters are significantly practical reagents since in situ generated unstable vinyl alcohol instantly and irreversibly tautomerizes to acetaldehyde, thus shifting the state of equilibrium in product direction. Therefore, in next experiments three different vinyl esters were used: vinyl acetate, vinyl butanoate, and vinyl decanoate. The influence of the acyl donor nature on the course of the alcohol acylation reaction was examined using 3 equiv of the appropriate vinyl ester under catalysis of the corresponding lipase (Novozym 435 or Lipozyme TL IM) suspended in MTBE as solvent, and stirred at 25 °C using a magnetic stirrer. All the assays were regularly traced by HPLC analyses after 1, 2, 4 and 7 days, respectively (Table 2). Consideration of the results summarized in Table 2 leads to the conclusion that the highest enantioselectivity (*E* = 844) of the reaction has been observed in the acylation of racemic alcohol (±)-**3** in the presence of Lipozyme TL IM, and vinyl acetate as an acyl transfer reagent. In accordance with the previous experiments, this extremely enantioselective lipase preparation afforded recovery of ester (*R*)-(−)-**6a** with perfect optical purity (>99% ee) at a conversion in the rage of 39–49% (Table 2, entries 4–7). On the other hand, since the Lipozyme TL IM-catalyzed acetylation tends to cease before achieving 50% conversion, the remaining alcohol (*S*)-(+)-**5** could be obtained only with a moderate enantiomeric excess (63–97% ee) reaching the highest optical purity (97% ee) after 7 days (Table 2, entry 7). In turn, KR of (±)-**3** conducted with vinyl acetate in the presence of Novozym 435 showed that after 24 h the enantiomeric purity of (*S*)-(+)-**5** increased over 77% ee and reached the 98% ee after 48 h (Table 2, entry 1). Complete optical purification (>99% ee) of the remaining alcohol (*S*)-(+)-**5** was observed after a reaction time of 4 days (Table 2, entry 4). As can be also seen from data presented in Table 2, both the rate and enantioselectivity of the enzymatic reactions could be significantly improved by using fatty acid vinyl esters. After analyzing the effect of those acyl group donors general findings can be presented: (i) (*S*)-(+)-**5** alcohol with excellent enantiomeric excess (>99% ee) could be obtained after a relatively short time of 24 h when vinyl butanoate was used, and the reaction was catalyzed by Novozym 435 (Table 2, entry 8); (ii) in turn, the highest enantiopurity of the formed (*R*)-enantiomer could be attained in the enzymatic transesterification proceeded with Lipozyme TL IM and vinyl butanoate as an acyl donor, appropriately arrested after 24 h, while 49% conversion was reached (Table 2, entry 11); (iii) another characteristic tendency was observed in the case of the velocity of Novozym 435-catalyzed reactions, in which the influence of the acyl group donor was pronounced more markedly than in the case of Lipozyme TL IM. For example, when the reaction was carried out with vinyl butanoate, Novozym 435 allowed 51% conversion to be achieved after remarkably 4-fold shorter reaction time compared with those conducted with vinyl acetate and vinyl decanoate (Table 2, Entry 8 vs 3 and 16), respectively.

**Table 2:** The influence of various acyl donors (3 equiv) upon the selectivity of Novozym 435 and Lipozyme TL IM for the *O*-acetylation of (±)-**3** in MTBE at 25 °C.

Entry	Enzyme^a^	Acyl donor	*t* (d)	conv.^b^ (%)	ee_s_^c^ (%)	ee_p_^c^ (%)	*E*^d^

1	Novozym 435	vinyl acetate	1	44	77	98	232
2			2	51	98	91	97
3			4	52	>99	90	99
4	Lipozyme TL IM		1	39	63	>99	382
5			2	45	80	>99	492
6			4	48	91	>99	637
7			7	49	97	>99	844

8	Novozym 435	vinyl butanoate	1	51	>99	94	170
9			2	53	>99	88	82
10			4	57	>99	76	37
11	Lipozyme TL IM		1	49	95	>99	747
12			2	50	>99	98	525
13			4	51	>99	94	170

14	Novozym 435	vinyl decanoate	1	48	91	98	317
15			2	49	>99	94	713
16			4	52	>99	91	111
17	Lipozyme TL IM		1	48	93	99	684
18			2	50	>99	98	525
19			4	50	>99	98	525

^a^Conditions: (±)-**3** 100 mg, lipase 20 mg, MTBE 2 mL, vinyl ester (3 equiv), 25 °C, 500 rpm (magnetic stirrer). ^b, c, d^See notes ^c, d^ and ^e^ above (Table 1).

As a conclusion, the enantioselectivity and rate of lipase-catalyzed KR of (±)-**3** in MTBE were significantly affected by the nature of an acyl transfer reagent, thus allowing the excellent enantiomer separation with 3 equiv of vinyl butanoate (Table 2, entries 11 and 12). However, enzymatic kinetic resolution with *E* = 747 has to be stopped at ca. 49% conversion in order to obtain (*R*)-(−)-**6b** in fully enantiopure form (>99% ee), and somewhat over 50% conversion in order to acquire the unreacted (*S*)-(+)-**5** at >99% ee and superb enantioselectivity (*E* = 525). This observation hints that careful monitoring of the reaction and its appropriate termination allows to receive both resolution products (*S*)-(+)-**5** and (*R*)-(−)-**6b** or (*R*)-(−)-**6c** with very high enantiopurity (98–100% ee) what may facilitate preparatory-scale synthesis. However, taking into account the clear advantages of vinyl acetate such as (i) the relatively high volatility (hence possibility of easy recovery of the product and remaining substrate), and (ii) lower cost when compared to the employed long-chain fatty acid vinyl esters, as well as the fact that (iii) vinyl acetate displayed acceptable impact on the performance of the lipases, we reasoned to set it as the reagent of choice for further up-scaling investigations using both heretofore examined enzymes.

### Preparative-scale lipase-catalyzed kinetic resolution of (±)-**3**

An optimized protocol for obtaining enantiopure key intermediates (*S*)-(+)-**5** and (*R*)-(−)-**6a–c** on a milligram-scale (100 mg) was successfully achieved. In order to provide further insight into enzymatic kinetic resolution of (±)-**3** enantiomers, and to show its potential as a method for asymmetric synthesis of enantiomerically pure pharmaceuticals **9** and **10**, this part of our study was aimed for investigating the feasibility of proceeding the enantioselective transesterification on a significantly larger scale. Bearing in mind that the fortune of this issue is critical for the development of a highly economic process at industrial scale, we decided to examine respective 10-fold, 20-fold and 30-fold linear enlargement of all the previously set parameters including the reagents concentrations, enzyme quantities and solvent volume under the optimized reaction conditions. Table 3 demonstrates the data of the lipase-catalyzed asymmetric *O*-acetylation of (±)-**3** in one-, two-, and three-gram-scale regarding their final effect on the total stereochemical outcome for the employed lipase preparations. We were pleased to see that the results were almost the same, regardless of the scale with, i.e., approx. 51% conversion after 4 days for the Novozym 435-mediated KR of racemic alcohol (±)-**3** (Table 2, entry 1 vs Table 3, entry 1). The kinetic resolution in the presence of this enzyme provided slower reacting enantiomer (*S*)-(+)-**5** with perfect optical purity (>99% ee), and its acetylated counterpart (*R*)-(−)-**6a** in enantioenriched form (94% ee). The separation of the resolution products on a silica gel column chromatography revealed that the unreacted alcohol (*S*)-(+)-**5** and the formed acetate (*R*)-(−)-**6a** could be prepared with isolated yield in the range of 81–95%, corresponding closely to the theoretical 50% proportions of the enantiomers in the racemate. Subsequent scaling of the reaction up to 2 and then up to 3 grams of the substrate (±)-**3** gave almost the same isolated yields of recovered alcohol (*S*)-(+)-**5** and the acetate (*R*)-(−)-**6a** with moderate to excellent enantiomeric excess for both compounds (69–100% ee) (Table 3, entries 2 and 3), respectively. Again, a very similar chirality inducement occurred under Lipozyme TL IM catalyzed conditions. In this manner, acetate (*R*)-(−)-**6a** was isolated chromatographically in 67% yield and excellent enantiomeric excess (>99%) reached when the reaction was arrested at 46% conversion after 3 days (Table 3, entry 4). From the same reaction mixture unreacted (*S*)-(+)-**5** was isolated with 84% ee. Independent experiment conducted with this lipase using 3 g of starting material (Table 3, entry 5) closely matched the above mentioned results showing that this approach effected the resolution of (±)-**3** into (*S*)-(+)-**5** alcohol (99% ee) and (*R*)-(−)-**6a** ester (86% ee) with a conversion value of 46% after 3 days. In the light of these findings, it became clear that this process proved to be flexible enough for being up-scaled in a straightforward manner. The amount of both enantiomers (*S*)-(+)-**5** and (*R*)-(−)-**7** obtained in the devised lipase-catalyzed resolution of (±)-**3** can be considered to be “in preparative scale” and ready-to-use for further transformations.

**Table 3:** Gram-scale enantioselective *O*-acetylation of (±)-**3** under kinetically controlled conditions with vinyl acetate in MTBE at 25 °C.

Entry	Enzyme	*t* (d)	conv.^a^(%)	ee_s_^b^ (%)/Yield^c^ (%)/[α]_D_^d^	ee_p_^b^ (%)/Yield^c^ (%)/[α]_D_^d^	*E*^e^

1	Novozym 435^f^	4	51	>99/95/+39.78 (*c* 1.02)	94/81/-8.75 (*c* 1.03)	170
2	Novozym 435^g^	3	57	>99/83/+35.00 (*c* 1.10)	75/86/-7.43 (*c* 1.01)	50
3	Novozym 435^h^	4	59	>99/86/N.D.^i^	69/N.D.^i^/N.D.^i^	27
4	Lipozyme TL IM^f^	3	46	84/72/+31.88 (*c* 1.49)	>99/67/−8.30 (*c* 1.44)	532
5	Lipozyme TL IM^h^	3	46	86/94/+29.56 (*c* 1.01)	99/92/−8.29 (*c* 0.91)	556

^a^The % conversion was calculated from the enantiomeric excess of the unreacted alcohol (ee_s_) and the product (ee_p_) according to the formula conv. = ee_s_/(ee_s_ + ee_p_). ^b^Determined by chiral HPLC analysis by using a Chiralcel OD-H column. ^c^Isolated yield after column chromatography (calculated on the basis of the theoretical number of moles arising from conversion rate, relative to theoretical amount, i.e., when 50% conversion is reached, up to the half of the acetate could be obtained). ^d^Specific rotation*, c* solution in methylene chloride, *T* = 22–23 °C, respectively (see Supporting Information File 1 for details). ^e^Calculated according to Chen et al. [70], using the equation: *E* = {ln[(1 – conv.)(1 – ee_s_)]}/{ln[(1 – conv.)(1 + ee_s_)]}. ^f^Conditions: (±)-**3** 1 g, lipase 0.2 g, MTBE 10 mL, vinyl acetate 1 g (3 equiv), 25 °C, 500 rpm (magnetic stirrer). ^g^Conditions: (±)-**3** 2 g, lipase 0.4 g, MTBE 20 mL, vinyl acetate 2 g (3 equiv), 25 °C, 500 rpm (magnetic stirrer). ^h^Conditions: (±)-**3** 3 g, lipase 0.6 g, MTBE 30 mL, vinyl acetate 3 g (3 equiv), 25 °C, 500 rpm (magnetic stirrer). ^i^Not detected.

### Determination of the stereochemistry of alcohol (+)-**5**

**Scheme 2:**
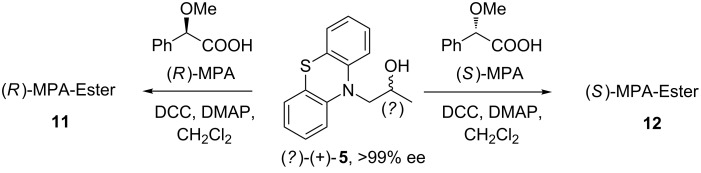
Assignment of the stereochemistry of enantiopure alcohol (+)-**5** resulting from derivatization with (*R*)- and (*S*)-MPA **11** and **12**, respectively.

The determination of the absolute configuration is crucial for all chiral molecules, but it is especially important for active pharmaceutical ingredients (APIs) like those presented herein, whereas only one enantiomer has a high positive pharmaceutical effect, while its counterpart is significantly less active. The assignment of stereochemistry of one of the kinetically resolved products on this stage could not only serve the information about the *catalytic behavior* of the enzymes but also could help us to plan further steps of the synthesis. Therefore, the stereopreference of the above-studied lipase preparations was evaluated by means of modified Mosher’s methodology described by Riguera et al. [71] In this relatively simple and widely-used experimental approach, the absolute configuration of the chiral substrate of unknown stereochemistry [in this case slower reacting alcohol (+)-**5** with an absolute enantiomeric purity (>99% ee)] was assigned by its independent reaction with the two enantiomers of an appropriate chiral derivatizing agent (CDA) followed by comparison of the ^1^H NMR spectra of the two diastereomeric derivatives obtained (**11** and **12**, Scheme 2). As already exemplified by us in few other reports [72–75], the modified Mosher’s methodology is easy to carry out and to interpret, and can be successively applied toward secondary alcohols with various heterocyclic substituents with a minimum of experimental work being invested as long as reliable CDA is employed. In all of our previous studies α-methoxy-α-phenylacetic acid (*O*-methylmandelic acid, MPA) used in both enantiomeric forms turned out to be an excellent chiral auxiliary reagent for this type of derivatized compounds since it has given diastereomeric esters that were distinguishable by NMR spectroscopy with reasonable levels of accuracy in terms of determination of stereochemistry (Scheme 2).

**Figure 2:**
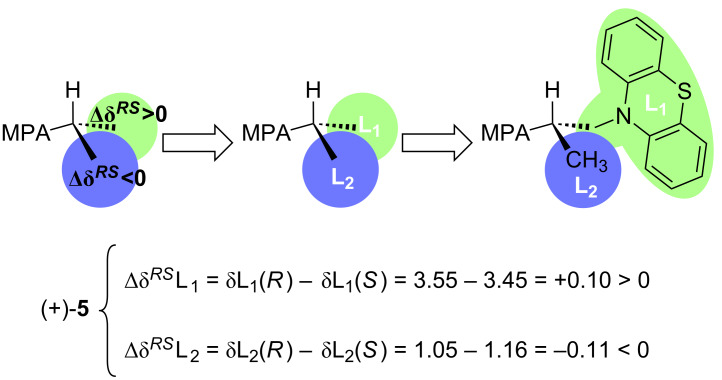
Description of substituents for determination of the absolute configuration of (+)-**5** and Δδ*^RS^* values obtained for the MPA esters **11** and **12**.

Subsequently, the chemical shifts of the substituents directly bonded to the stereogenic centre of the investigated alcohol (+)-**5** (L_1_/L_2_) were compared, and their spatial location (absolute configuration) was determined in accordance with the signs of their differences (Δδ*^RS^*L_1_ and Δδ*^RS^*L_2_) in both prepared MPA-derivatives **11** and **12** (Figure 2).

**Figure 3:**
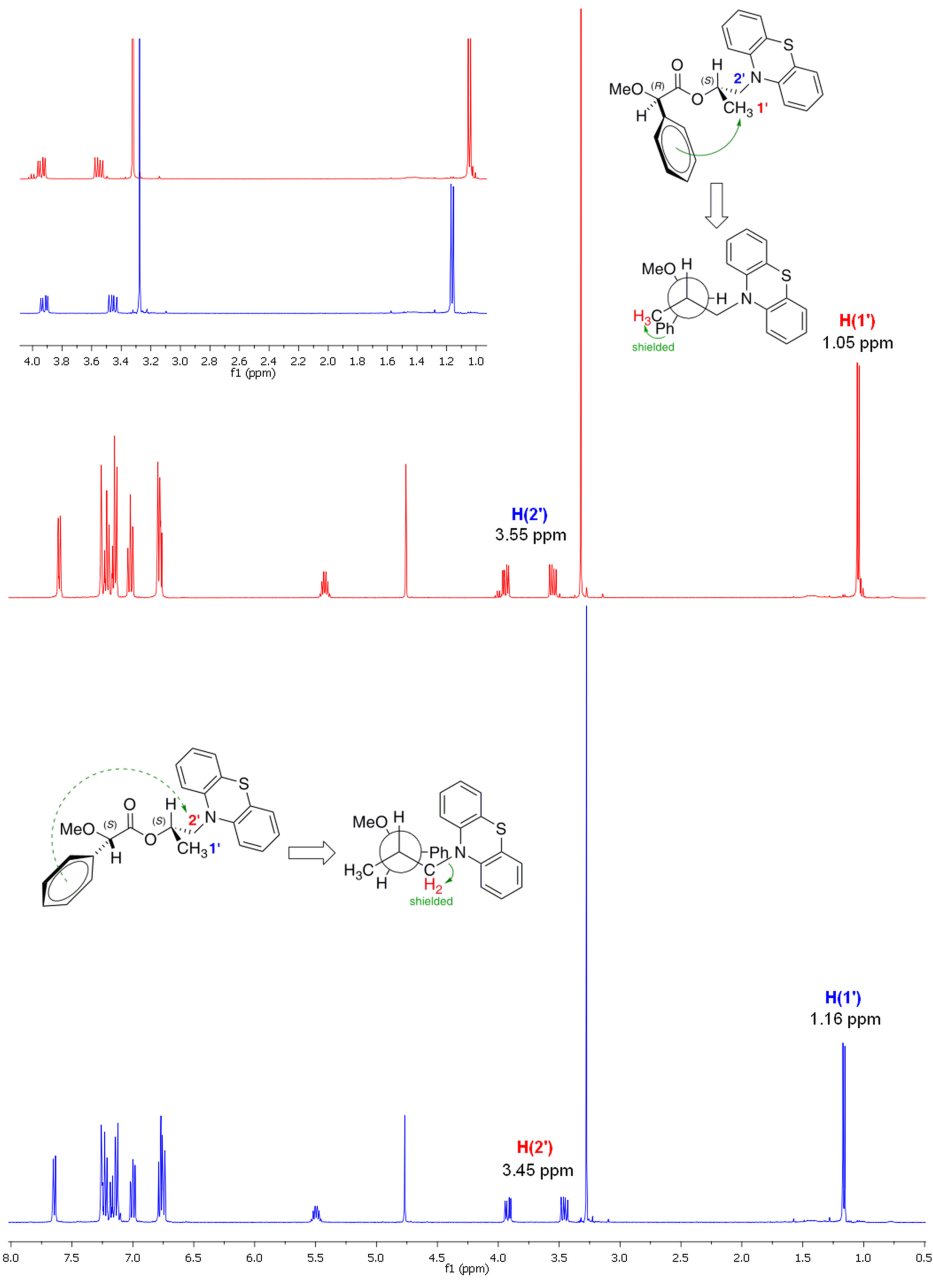
^1^H NMR (CDCl_3_, 400 MHz) spectra of the (*R*)-MPA **11** (red colored line) and (*S*)-MPA and **12** (blue colored line) derivatives of enantiopure alcohol (+)-**5** (>99% ee). Additionally, with red color are marked protons shielded by the phenyl ring of chiral auxiliary, blue labels stand for unaffected protons. Green arrows indicate the shielding effect caused by the aromatic system.

The chirality of asymmetric carbon in the alcohol (+)-**5** could be established due to the following facts: (i) It has been empirically confirmed by Latypov et al. [76] (as a result of dynamic NMR studies) that a final prevalent conformation in the CDA-substrate system in the case of MPA esters of secondary alcohols renders the *sp* rotamer, in which the methoxy group, the Cα carbon, and the carbonyl group of the MPA unit as well as the methine proton (CH) of the alcohol moiety [the substrate (+)-**5** part] are all situated in the same plane, whereas the phenyl ring is ca. perpendicular to the C=O bond and coplanar with the C_α_H bond. Both the methoxy and the carbonyl group are in a syn-periplanar disposition (see Newman projections of the appropriate (1*R*,2*S*)- and (1*S*,2*S*)-conformations in diastereomers **11** and **12** depicted in Figure 3). (ii) The main induced anisotropic magnetic field effect (shielding), which is generated by means of high π electron density (present in the phenyl ring of the chiral auxiliary regent MPA), showed appropriate strength (intensity) giving rise to perceptible shifts in the ^1^H NMR signals of the L_1_/L_2_ groups located close to the chiral carbon in each of the investigated derivatives **11** and **12**. (iii) The ^1^H NMR signals of the chiral MPA shift reagent did not overlap with those of the examined alcohol (+)-**5** thus allowing easy interpretation of the recorded spectra.

Comparing the recorded ^1^H NMR spectra of both CDAs derivatives **11** and **12** illustrated in Figure 3, the most remarkable features should be outlined as follows. The ^1^H NMR data of (*R*)-MPA ester **11** show a general shielding effect as a consequence of the close proximity of the phenyl group, with important upfield shifts (lower frequency) for the signals assigned to H(1’) hydrogen atoms.

In turn, this particular H(1’) signals in the spectrum for (*S*)-MPA derivative **12** are observed as unaffected and thus a downfield shift of the resonance signal occurs. The opposite phenomenon can be noticed when the protons of the methylene group H(2’) in both MPA esters **11** and **12** are taken into account. In this situation, the H(2’) protons of (*S*)-MPA derivative **12** are located under the shielding cone of the phenyl ring, which resulted in an upfield shift of the ^1^H NMR signals in comparison to the signals from the (*R*)-MPA derivative **11**.

The recognition of the absolute configuration of (+)-**5** clearly indicates that both Novozym 435 and Lipozyme TL IM lipases follow Kazlauskas’ rule [77], according to which the (*R*)-enantiomer of the chiral substrate (±)-**3** is preferentially acetylated.

**Figure 4:**
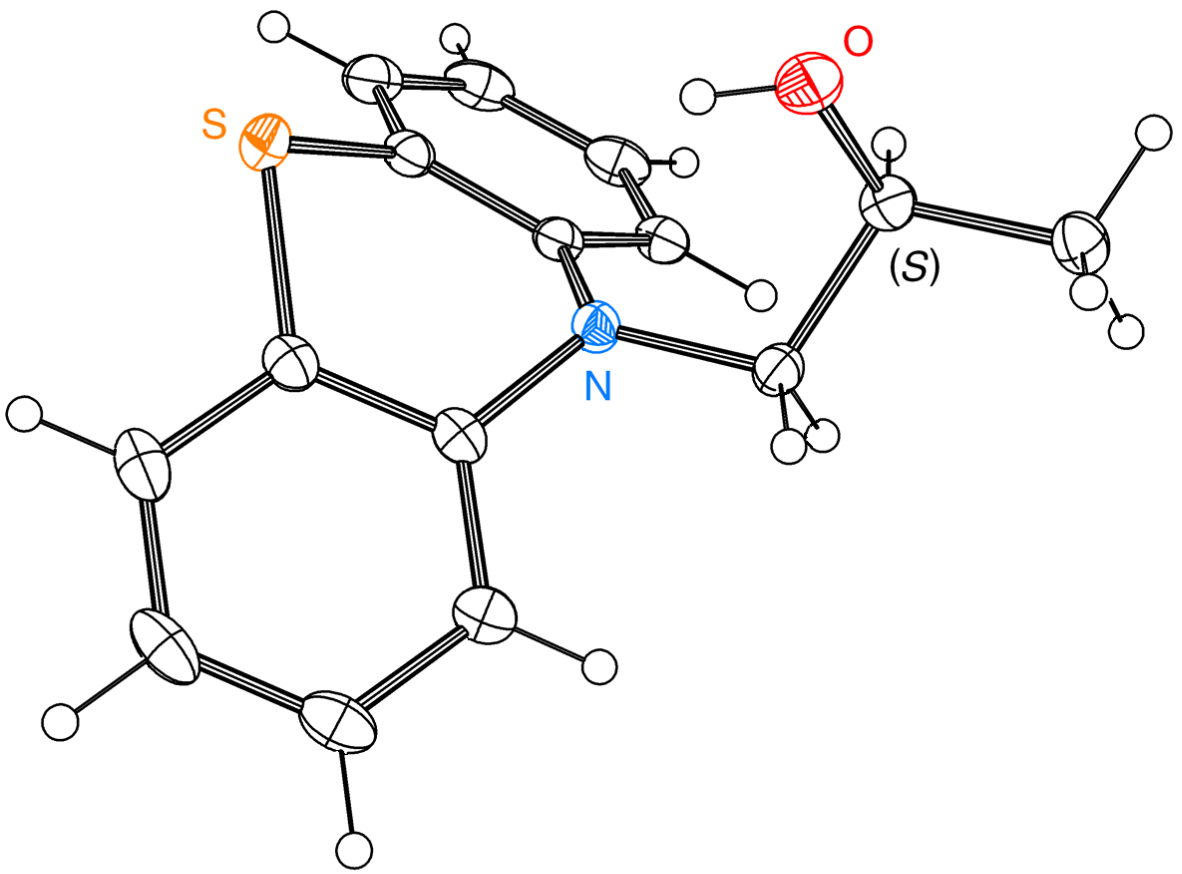
An ORTEP plot of (*S*)-(+)-1-(10*H*-phenothiazin-10-yl)propan-2-ol (*S*)-(+)-**5**. The following crystal structure has been deposited at the Cambridge Crystallographic Data Centre and allocated the deposition number CCDC-1018655.

In order to unambiguously confirm the adequacy of ^1^H NMR shift analysis, the single crystal of enantiomerically pure alcohol (+)-**5** (>99% ee) was subsequently analyzed by X-ray diffraction (XRD) method (Figure 4), which confirmed (*S*)-configuration and allowed us to conclude that Mosher’s methodology can serve as a reliable determination method of the absolute configuration toward this type of compounds. For details concerning crystallization procedure and XRD measurements see Supporting Information File 1.

### Approaches to the preparation of both enantiomeric forms of **9** and **10**

**Scheme 3:**
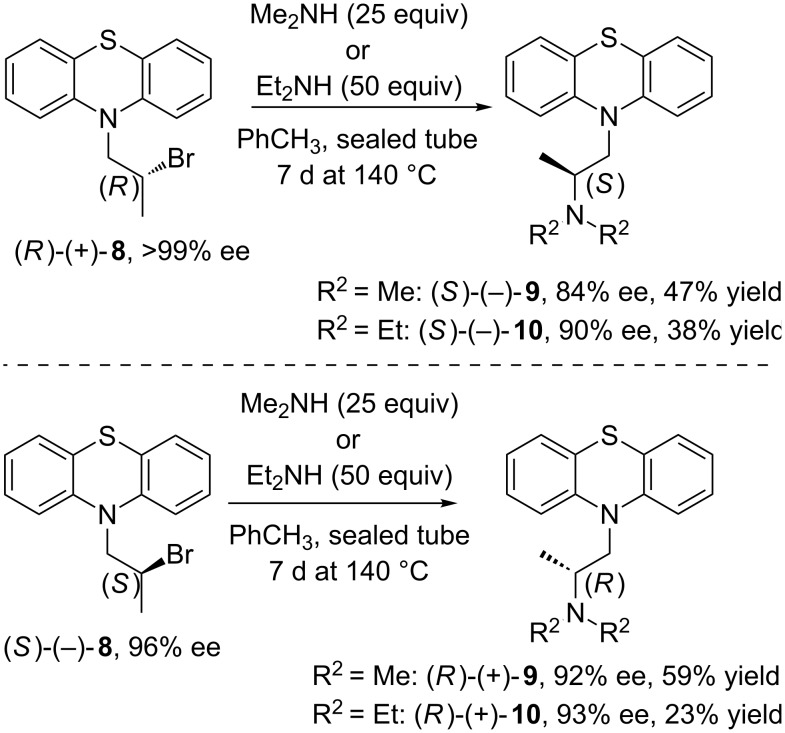
Amination of optically active bromo derivatives (*R*)-(+)-**8** or (*S*)-(−)-**8** in toluene.

Although, the remaining synthesis of the final products **9** and **10** appears deceptively straightforward, it actually turned out to be a considerable challenge as the inversion of the chiral center in the intermediate molecules **3** and **8** has been identified as a key step in optimizing the process to avoid racemization. To save one step less, we initiated our efforts by direct transformation of the *sec*-alcohol functionality in afore-obtained compound (±)-**3** under modified Mitsunobu [78–79] reaction conditions using Ph_3_P (1.1 equiv), DEAD (1.1 equiv), and the respective amine (1 equiv of Et_2_NH or Me_2_NH) conducted in dry THF. Since the Mitsunobu condensation protocol is considered as particularly attractive due to the superior reactivity of the in situ-generated corresponding oxyphosphonium intermediate on nucleophilic substitution, we envisioned that in this scenario the hydroxy moiety would be inverted under mild reaction conditions and in a desired one-pot stereospecific manner. To validate this hypothesis, the reaction was at first performed using model racemic alcohol (±)-**3**, and in two variants of reagents addition order. To our disappointment, none of these attempts led to success. When Mitsunobu chemistry fails, a hydroxy group inversion by tosylation and displacement using amine was performed. Unfortunately, attempts to carry out the reaction at higher temperature led to complete decomposition of the tosylate substrate. In the forward direction to synthesize optically active pharmaceuticals **9** and **10**, we decided to prepare both enantiomers of corresponding bromide derivative **8**. This was obtained by treatment of afore-prepared enantiopure alcohol (*S*)-(+)-**5** with 1 equiv of phosphorus tribromide (PBr_3_) at room temperature carried out for 2 h. This allows to access (*R*)-(+)-**8** in >99% ee and in 27% yield. In turn, to afford its bromide counterpart (*S*)-(−)-**8** a two-step reaction sequence has to be performed. At first, optically active acetate (*R*)-(−)-**6a** was transformed into the corresponding alcohol (*R*)-(−)-**7** through simple cleavage of the acetyl group by means of NaOH-mediated mathanolysis. This resulted in the formation of (*R*)-(−)-**7** in high 76% yield and almost without loss of the optical purity (98% ee). Secondly, thus-obtained (*R*)-(−)-**7** was converted to (*S*)-(−)-**8**, but surprisingly with partial loss in enantiomeric excess (96% ee) despite using the same procedure as for the reaction between (*S*)-(+)-**5** and PBr_3_. In addition, bromination of (*S*)-(+)-**5** under Appel reaction conditions employing tetrabromomethane (CBr_4_) as a halide ion source used along with triphenylphosphine (Ph_3_P) was also investigated. Although this approach proceeded to give (*R*)-(+)-**8** in considerably higher yield (77%), the obtained result was inferior from the stereochemical point of view as the reaction was non-selective and led to afford (*R*)-(+)-**8** of poor 23% ee. With both enantiomers of bromo derivative (*R*)-(+)-**8** and (*S*)-(−)-**8** in hand, we have subsequently proceeded amination with Et_2_NH and Me_2_NH, respectively. In all cases, a sealed glass vessel was used since these reactions need to be operated mostly at elevated temperatures, and low-boiling amines (bp. in the range of 54–60 °C) could be lost in the course of the process. The first set of reactions was performed by stirring racemic bromide (±)-**8** in an appropriate amine reagent for 24 h at room or elevated temperature (>60 °C). Unfortunately, all of these attempts failed to provide satisfactory results and led only to the recovery or the substantial decomposition of the starting material (±)-**8**. We then turned our attention to the conventional protocol optimizing *N*-alkylation by screening several aprotic polar solvents (CH_3_CN, DMF, dioxane and CH_2_Cl_2_) at room temperature or at heating, respectively. Under most of the aforementioned conditions, (±)-**8** proved to be reluctant to form respective tertiary amine products **9** or **10**, and only in the case of substitution with Me_2_NH the reactions proceeded in both media (CH_3_CN and dioxane), however sluggishly and somewhat low-yielding typically obtaining (±)-**9** in less than 5%. In the course of investigating why (±)-**8** failed to react with amines, we focused on employing various basic conditions (EtN_3_, K_2_CO_3_, *n*-BuLi), under which activation of the *N*-nucleophile might increase the reactivity and circumvent encountered drawbacks. Disappointingly, treatment of (±)-**8** with EtN_3_ showed no positive effect on the reaction progress, and when K_2_CO_3_ or *n*-BuLi were applied it led only to exhaustive decomposition of the starting material (±)-**8** producing a complex mixture. To improve the efficiency of the desired process, various catalytic conditions were subsequently evaluated. Initially, we decided to employ PTC-assisted amination in the similar manner to those reported by O’Meara [80] and Durst et al. [81], respectively. A number of different variants of these methods including use of *N*-benzyl-*N*,*N*,*N*-triethylammonium chloride [TEBA(Cl)] or dibenzo-18-crown-6 as the catalyst and a liquid–solid system comprising 1.6 equiv of grounded NaOH suspended in DMSO or a liquid–liquid systems composed of 50% NaOH or 60% KOH and the appropriate solvent (CH_2_Cl_2_, DMSO) stirred along with Et_2_NH at room or slightly increased temperature (40 °C) have been studied. However, none of the attempted changes led to an improvement in the reaction progress. By the same token, another catalytic approach using solid Ag_2_O was adopted in accordance to a method proposed by D'Angeli et al. [82]. Since the authors of this report suggested that solid Ag_2_O behaves as both a Lewis acid and Brønsted base acting synergically on the investigated 2-bromo amides, we have assumed that the extension of this concept on our reaction could activate substrate (±)-**8** and thus promote the desired *N*-alkylation. And this time, application of Ag_2_O was not successful since this reaction has been plagued by the formation of an intractable mixture of side products. The resistance of (±)-**8** toward nucleophilic substitution in both studied catalytic systems (PTC- and Ag_2_O-mediated approaches) may be explained by the fact that our substrate (±)-**8**, in contrast to those described in the literature, lacks of the neighboring carbonyl group, which presumably favored the reported processes due to weakening of the carbon–bromine bond. Next, we aimed to investigate the amination of (±)-**8** using Et_2_NH in toluene medium hoping that the employment of this aprotic and less polar solvent could shift the equilibrium toward the product formation if (±)-**10** precipitated over the course of reaction in hydrobromide salt form. To our delight, when heating this reaction mixture at 110 °C for 96 h some progress has been observed. Therefore, to improve the efficiency of this process, our next attempt involved the increase in the operating temperature (up to 140 °C of an oil bath) as well as elongation of the reaction time, what finally provided (±)-**10** in 38% yield after 7 days. An extension of this condition for the reaction between (±)-**8** and Me_2_NH gave even a better result in terms of the product (±)-**9** yield (47%). However, when the above-performed reactions were repeated with optically active bromo derivatives (*R*)-(+)-**8** (>99% ee) and (*S*)-(−)-**8** (96% ee), it turned out that the respective products (*S*)-(−)-**9** (84% ee) and (*R*)-(+)-**9** (92% ee) or (*S*)-(−)-**10** (90% ee) and (*R*)-(+)-**10** (93% ee) were obtained with partial drop of the enantiomeric excess (Scheme 3). These facts plus the modest yields (<50%) as well as long reactions time (7 days) limited this method as being a preparative one, and forced us to search for an alternative method.

**Scheme 4:**
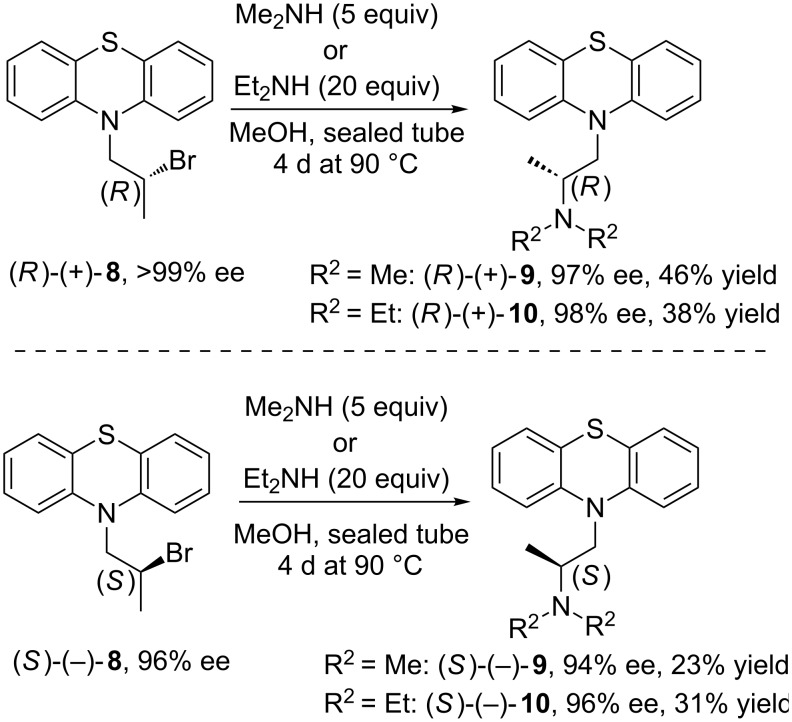
Amination of optically active bromo derivatives (*R*)-(+)-**8** or (*S*)-(−)-**8** in methanol.

Somewhat more surprisingly, the best results were obtained when the reaction was performed in MeOH similarly to the rivastigmine synthesis strategy published by Sethi and co-workers [83], but at higher temperature (90 °C of an oil bath) since at reported 25 °C the reaction with substrate (±)-**8** did not occur. This modification led to obtain (±)-**9** in yield of 38% and (±)-**10** in yield of 71%, respectively. A simple change of the solvent to methanol also drastically improved the rate as the reactions needed at least 3 days less when compared to those conducted in toluene and what is crucial at lower temperature. Moreover, under this conditions optically active substrate (*R*)-(+)-**8** (>99% ee) underwent amination selectively almost without loss of optical purity affording unexpectedly enantioenriched products (*R*)-(+)-**9** (97% ee) and (*R*)-(+)-**10** (98% ee) with reverse stereochemistry at a chiral carbon. Compared to the previously obtained products this seems to be more likely than the net retention (double inversion) occurred during this process. Their respective counterparts including (*S*)-(−)-**9** (94% ee) and (*S*)-(−)-**10** (96% ee) were obviously obtained in less enantiomerically pure forms as a consequence of preparing them from less enantiopure substrate (*S*)-(−)-**8** (96% ee) (Scheme 4).

**Scheme 5:**
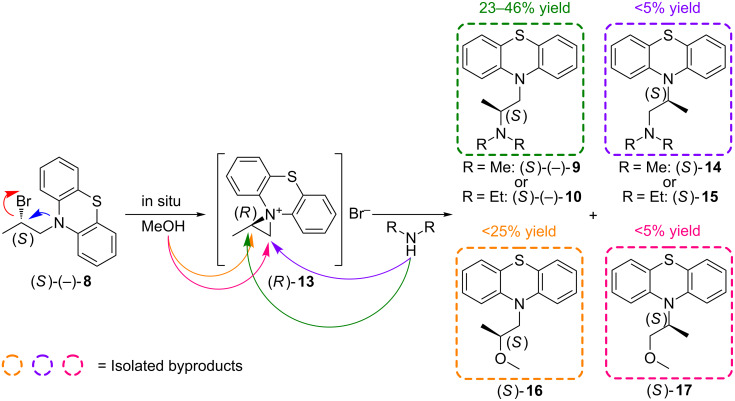
The proposed reaction mechanism for amination of optically active (*S*)-(−)-**8** in methanol.

The results of above described experiments can only be rationalized considering the formation of an intermediate aziridinium ion (*R*)-**13** upon heating in MeOH, which is then attacked by amine at the more hindered carbon atom of the aziridine ring. These observations allowed us to propose the following plausible mechanism (Scheme 5), postulated mainly on the basis of the signs of specific rotation and the enantiomeric elution order from chiral HPLC, which were opposite to those corresponding to the appropriate products obtained when toluene was applied as a solvent (see Supporting Information File 1). This phenomenon was also confirmed by isolation of byproducts characteristic for this reaction mechanism including (2*S*)-*N*,*N*-dimethyl-2-(10*H*-phenothiazin-10-yl)propan-1-amine (*iso*-promethazine) (*S*)-**14** or (2*S*)-*N*,*N*-diethyl-2-(10*H*-phenothiazin-10-yl)propan-1-amine (*iso*-ethopropazine) (*S*)-**15** (depending on the amine used). What is even more compelling 10-[(2*S*)-2-methoxypropyl]-10*H*-phenothiazine (*S*)-**16** and 10-[(2*S*)-1-methoxypropan-2-yl]-10*H*-phenothiazine (*S*)-**17** as a result of a competitive reaction between (*R*)-**13** and methanol (Scheme 5) were isolated and characterized as well.

Moreover, an unexpected regioselectivity upon ring opening by amines has been observed. Apparently, this aziridinium salt (*R*)-**13** is opened exclusively at the more hindered aziridine carbon atom since the respective products (*S*)-(−)-**9** or (*S*)-(−)-**10** (23–46% yield) and (*S*)-**16** (<25% yield) have been isolated in majority, while yield of (*S*)-**17** and both unwanted isomers (*S*)-**14** or (*S*)-**15** remained negligible (<5%). The outcome of the reaction is quite interesting since the conversion of, i.e., (*S*)-bromide into the (*S*)-configurated product has proceeded with overall retention of configuration via regio- and stereospecific opening of the aziridinium ion (*R*)-**13** at the activated *pseudo*-benzylic position, where the partial positive charge (δ^+^) is obviously better stabilized than at the neighboring carbon atom. This clearly suggests that electrostatic factors (interactions) are favored over steric hindrance effects giving the opportunity of a purely solvent-dependent stereodivergent synthesis of therapeutically active ingredients **9** and **10** in a highly stereoselective fashion.

In view of this findings, it appeared that deterioration of the enantiomeric excess of the both APIs isomers [Δ% ee = 9–15 for (*R*)-(+)-**8** (>99% ee) and Δ% ee = 3–4 for (*S*)-(−)-**8** (96% ee)] synthesized in toluene may be related to the fact that this process proceeded at least in part through the formation of activated aziridinium ion intermediate (*S*)-**13** or (*R*)-**13** (net retention), and not only via direct attack of the amine on the appropriate bromo derivative (*R*)-(+)-**8** or (*S*)-(−)-**8** (single inversion). Toluene as an aprotic-lipophilic solvent is rather responsible for unfavorable conditions for the formation of aziridinium salt due to ineffective stabilization of the cationic species. Nevertheless some portions of the bromo derivative **8** most likely undergo intramolecular nucleophilic substitution by means of the neighboring amine moiety present in the phenothiazine ring, and by this affecting the optical purity of the products.

### Determination of the enantiomeric purity of promethazine **9** and ethopropazine **10**

In order to determine the enantiomeric excess of the final pharmaceutical products **9** and **10**, we initially examined the chiral HPLC conditions proposed by Ponder et al. [52]. Among various HPLC columns studied in that paper, we have owned the Chiralcel OJ with cellulose-derived chiral stationary phase (CSP) consisting of cellulose tris(4-methylbenzoate) as chiral selector. Unfortunately, when using this column accordingly to Ponder’s indications we could only separate promethazine (±)-**9** base enantiomers (see Supporting Information File 1 for details). Since ethopropazine (±)-**10** enantiomers were not resolvable on a Chiralcel OJ column under various HPLC conditions performed in the normal phase mode, we have opted to investigate several other polysaccharide type columns including Chiralcel OD-H, Chiralcel OJ-H, Chiralcel OJ-RH and Chiralpack IA. Unfortunately, all the studied chromatographic analyses led to failure as no signs of enantiomeric separations for (±)-**10** were obtained. Therefore, we decided to investigate enantiomeric separation conditions applying a common NMR methodological approach, which assumes the use of a lanthanide-like chiral shift reagent (CSR) such as europium(III) tris[3-(heptafluoropropylhydroxymethylene)-(+)-camphorate [Eu(hfc)_3_] [84–86]. To our disappointment, the desirable enantiomeric shift difference magnitudes and enhanced spectral resolution for the studied (±)-**10** were not achieved. Under these particular circumstances we were forced to undertake another strategy, which would provide highly accurate and rapid access to quantification of the enantiomeric excess of optically active ethopropazine **10**. Finally, the assessment of enantiopurity of the synthesized (*S*)-(−)-**10** and (*R*)-(+)-**10** were determined by HPLC, but using two serially connected Chiralcel OJ columns and mobile-phase system composed of *n*-hexane/*tert*-BuOH/Et_3_N (98:1.5:0.5, v/v/v) at a flow rate of 1.1 mL/min similarly to the method reported by Šinko et al. [57].

## Conclusion

In summary, a straightforward chemoenzymatic preparation strategy of both highly enantioenriched enantiomeric forms of promethazine **9** (up to 97% ee) and ethopropazine **10** (up to 98% ee), with the chirality being introduced by the action of lipases has been devised. An extensive screening revealed both Novozym 435 and Lipozyme TL IM lipase preparations to be capable of asymmetric enantioselective transesterification of racemic 1-(10*H*-phenothiazin-10-yl)propan-2-ol ((±)-**3**). Kinetic resolution of (±)-**3** using the respective enzymes, and vinyl acetate as the acylating agent have provided an efficient access to highly enantioenriched alcohols (*S*)-(+)-**5** (>99% ee) and (*R*)-(−)-**7** (98% ee) accomplished in high isolated yields (up to 95%). Most of the critical enzymatic reaction factors such as medium, acyl group donor and operating time were investigated in depth to establish the most optimal biotransformation conditions. The obtained results of enzymatic reactions conducted in small scale (100 mg) were scaled-up with high correlation to a synthetically useful substrate amount of 3 g (±)-**3** (0.4 M concentration). Based on these findings, we can speculate that the developed procedure can facilitate a potential commercial application in the production of title drugs **9** and **10** with a good perspective in industrial scale since high conversions and excellent ee values can be achieved with low catalyst loadings. The ^1^H NMR spectroscopic assignment of the absolute configuration of the slower reacting enantiomer (+)-**5** of enzymatic KR was performed by means of a modified Mosher’s methodology, and was supported by single-crystal X-ray diffraction (XRD), which both led up to the final conclusion that the employed lipase preparations exhibit (*R*)-stereopreference towards the enantiomers of resolved alcohol (±)-**3**. The products of the enzymatic transformations were functionalized en route to the title compounds via a two-step reaction sequence using the appropriate brominating agent (PBr_3_), and subsequent amination of the thus-prepared halo derivative by means of respective aliphatic amines. A systematic investigation of the amine functionalization approach by examining various reaction parameters including solvents, catalysts, bases, additives, and operating temperatures revealed some unexpected results as the transformation of bromo derivative **8** is highly dependent on the proper choice of the reaction medium system. It turned out that in toluene solution a typical S_N_2 reaction is observed (single inversion), whereas in methanol an intramolecular cyclization occurred leading to aziridinium ion-formation, which only then reacted with the amine giving the product of double inversion. In the case of reactions carried out in methanol only a negligible loss of the enantiomeric purity (94–97% ee for **9** and 96–98% ee for **10**) was observed, while the reactions conducted in toluene proceeded with higher level of deterioration in terms of enantiomeric excess values (84–92% ee for **9** and 90–93% ee for **10**).

## Supporting Information

File 1 Complete experimental procedures and characterization data.
