# Supplementary material for: First chemoenzymatic stereodivergent synthesis of both enantiomers of promethazine and ethopropazine
Source: Beilstein J Org Chem. 2014 Dec 18;10:3038–55. doi: 10.3762/bjoc.10.322 (PMC4311712; doi:10.3762/bjoc.10.322)
Supplement: File 1 — Complete experimental procedures and characterization data. [file Beilstein_J_Org_Chem-10-3038-s001.pdf]

Supporting Information

for

First chemoenzymatic stereodivergent synthesis of both enantiomers of promethazine and ethopropazine

Paweł Borowiecki*, Daniel Paprocki and Maciej Dranka

Address: Warsaw University of Technology, Faculty of Chemistry, Noakowskiego St. 3, 00-664 Warsaw, Poland

Email: Paweł Borowiecki* - pawel_borowiecki@onet.eu

*Corresponding author

Complete experimental procedures and characterization data

<u>Table of contents</u>

General details …………………………………………………………… S2–S4

Compound characterization data and representative synthetic procedures including X-ray diffraction data ……………………………….……….. S4–S15

Table S1. Analytical separation conditions of different compounds enantiomers by Chiralcel OD-H and Chiralcel OJ (Daicel) chiral columns …………...... S16

Print screens of HPLC chromatograms ……..…………………..……... S17–S26

Copies of NMR, HRMS, IR, GC and UV/VIS spectra ……………..…. S27–S57

References ………………………………………………..…………..… S57

General details

All commercially available reagents [Sigma Aldrich Chemie GmbH (Schnelldorf, Germany), Fluka (Seelze, Germany) and POCH (Poland)] were used without further purification. Solvents (methylene chloride, tetrahydrofuran) were dried by simply allowing them to stand over activated (oven-roasted in high-vacuum) 3Å molecular sieves [20% mass/volume (m/v) loading of the desiccant] at least for 48 h [1]; *n*-Hexane and ethyl acetate were purified by fractional distillation before use. All non-aqueous reactions were carried out under oxygen-free (argon-protective) conditions using over-dried glassware; all the reactions with low-boiling dimethyl- and diethyl- amines were performed in sealed reaction vessel equipped with Teflon-cap (Sigma Aldrich Ace pressure tube, volume ca. 25 mL). Lipase from *Candida antarctica B* [Novozym 435 - immobilized on a macroporous acrylic resin, specified activity: >10000 U/g or 10 PLU/mg, purchased from Novo Nordisk; Chirazyme L-2, c.-f., C2, Lyo. - carrier-fixed on (carrier 2), and Chirazyme L-2, c.-f., C3, Lyo. - carrier-fixed on (carrier 3), specified activity: 150 kU, both enzymes purchased from Roche], lipase from *Candida antarctica A* [Lipase A - cross-linked enzyme aggregate (CLEA), specified activity: ≥1.5 U/mg, purchased from Sigma (cat. nr.: 16698)], lipase from *Burkholderia* (formerly *Pseudomonas*) *cepacia* [Lipase, immobilized on Immobead 150 from *Pseudomonas cepacia* (PS-Immobead 150), specified activity: ≥900 U/g, purchased from Sigma (cat. nr.: 54327), Amano PS - native lipase, specified activity: >23.000 U/g, Amano PS-IM - immobilized on diatomite, specified activity: 500 U/g, Amano PS-C II - immobilized on ceramic, specified activity: ≥30.000 U/g, all enzymes purchased from Amano Pharmaceutical Co., Ltd.], lipase from *Pseudomonas fluorescens* [Amano AK - native lipase, specified activity: >20.000 U/g, purchased from Amano Pharmaceutical Co., Ltd.], lipase from *Candida cylindracea* (also named *Candida rugosa*) [CCL, specified activity: 12.000 IU/g, purchased from Sigma], lipase from *Mucor javanicus* [Amano Lipase M - native lipase, specified activity: >10.000 U/g, purchased from Sigma (cat. nr.: 534803)], lipase from *Thermomyces lanuginosus* [Lipase, immobilized on Immobead 150 from *Thermomyces lanuginosus* (*Thermomyces lanuginosus*-Immobead 150), specified activity: ≥3000 U/g, purchased from Sigma (cat. nr.: 76546), and Lipozyme TL IM - immobilized on silica gel (a silica granulated), specified activity: 170 IUN/g, purchased from Novozymes (Bagsvaerd, Denmark)], lipase from *Rhizomucor miehei* [Lipozyme RM IM – commercially immobilized, specified activity: 150 IU/g, was purchased from Novozymes A/S (Bagsvaerds, Denmark)]. All enzymes studied were used without any pretreatment. Analytical scale enzymatic reaction were performed in thermo-stated glass vials (V = 4 mL) placed in Chemglass CG-1991-04 GOD Anodized Aluminum Reaction Block, 48

position 48, 19 mm hole depth, for circular top Hot plate stirrer. Melting points were obtained with an MPA100 Optimelt SRS apparatus and are uncorrected. Thin-layer chromatography was carried on TLC aluminum plates with silica gel Kieselgel 60 F_{254} (Merck, Germany) (0.2 mm thickness film) using UV light as a visualizing agent. Preparative separations were carried out by: (i) column chromatography using Merck silica gel (230-400 mesh), with grain size 40-63 μm or by (ii) PLC PSC-Fertigplatten Kieselgel 60 F_{254} (20 × 20 cm with 2 mm thickness layer) glass plates purchased from Merck, Germany. The gas chromatographic (GC) analyses were performed with an HP Series II 5890 instrument (Maryland, United States) equipped with a flame ionization detector (FID) and fitted with HP-50+ (30 m) semi-polar column; the GC injector was maintained at 250 °C; Column temperature programs are given for respective analytes in the compound characterization data paragraphs; Helium (2 mL/min) was used as carrier gas; retention times (t_R) are given in minutes under these conditions. The enantiomeric excesses (% ee) of optically active compounds were determined by high performance liquid chromatography (HPLC) analyses performed on Shimadzu CTO-10ASV chromatograph (Shimadzu Corporation, Japan) equipped with STD-20A UV detector and Chiralcel OD-H (Daicel Chemical Industries Ltd., Japan) chiral column using mixtures of *n*-hexane/*iso*-propyl alcohol or Chiralcel OJ (Daicel Chemical Industries Ltd., Japan) chiral column using mixtures of *n*-hexane/ethyl alcohol or *n*-hexane/*tert*-butanol/triethyl amine as mobile phase in appropriate ratios given in experimental section (see later for further details for each individual compound); both chiral columns were analytical type, dimension 250 mm x 4.6 mm; the HPLC analyses were executed in an isocratic manner if not stated otherwise; flow (*f*) is given in mL/min; racemic compounds were used as standards; HPLC conditions and retention times (t_R) are given in Table S1. Optical rotations were measured with a P20 polarimeter (Belligham & Stanley Ltd.) in a 2 dm long cuvette using the sodium D line (589 nm); $[\alpha]_D$ are given in units of: deg $dm^{-1}cm^3$ g^{-1}; the concentration *c* is in g/100 mL. UV spectra were measured with a Varian Cary 3 UV-Visible Spectrophotometer (Varian, Inc., USA). ^1H and ^{13}C NMR spectra were measured with a Varian Mercury 400BB spectrometer (Varian, Inc., USA) operating at 400 MHz for ^1H and 100 MHz for ^{13}C nuclei; chemical shifts (δ) are given in parts per million (ppm) on the delta scale related to the solvent peak used as reference value; signal multiplicity assignment: s, singlet; d, doublet; t, triplet; q, quartet; m, multiplet; coupling constant (*J*) are given in hertz (Hz); All samples were recorded as solutions in fully deuterated chloroform ($CDCl_3$), methanol (CD_3OD) or benzene (C_6D_6), respectively. All NMR reports for Supporting Information document were created by ACD/NMR Processor Academic Edition 12.0. (Freeware software provided by ACD/Labs, USA & Canada) and show only delta range where signals were present. Mass spectra were

recorded on a Micro-mass ESI Q-TOF spectrometer in ESI mode; sample of analyte (2 mg) was prepared by dissolving it in methanol (1 mL). The spectrometer had an electrospray ion source and a linear ion trap analyzer. FT-IR spectra of neat samples were recorded on a Perkin Elmer System 2000 FTIR Spectrometer equipped with a Pike Technologies GladiATR attenuated total reflectance (ATR) accessory with a monolithic diamond crystal stage and a pressure clamp; FT-IR spectra were recorded in transmittance mode in the 300-4000 cm^{-1} range, in ambient air at room temperature, with 2 cm^{-1} resolution, 0.5 cm^{-1} interval and accumulation of 32 scans; unit are given in %T.

Compound characterization data and representative synthetic procedures:

Synthesis of 1-(10*H*-phenothiazin-10-yl)propan-2-ol (±)-3

Method A: To a stirred solution of 10*H*-phenothiazine **1** (2 g, 10 mmol) in dry THF (70 mL) at -78 °C *n*-butyllithium (9.4 mL of 1.6 M solution in hexane, 15 mmol) was added dropwise in gentle flow of argon. After 1 h of stirring, propylene oxide **2** (1.16 g, 20 mmol, 1.4 mL) was added dropwise, then cooling bath was removed, and the reaction mixture was stirred overnight at room temperature. The reaction mixture was subsequently quenched by adding finely crushed ice (10 g), then extracted with Et_2O (3 × 20 mL). The combined organic layer was washed with water (100 mL), combined and dried over anhydrous $MgSO_4$. Evaporation of the solvent gave crude product as an oily residue, which was purified by column chromatography on silica gel using mixture of hexane/AcOEt (80:20, v/v) as an eluent. After high-vacuum drying the desired alcohol crystallized as light-gray solid (1.84 g, 7.15 mmol, 71%).

Method B: To a stirred solution of 10*H*-phenothiazine **1** (5 g, 25.1 mmol) in dry THF (120 mL) at -78 °C *n*-butyllithium (23.5 mL of 1.6 M solution in hexane, 37.6 mmol) was added dropwise in gentle flow of argon. After 1 h of stirring, propylene oxide **2** (2.91 g, 50.2 mmol, 3.5 mL) was added dropwise, then cooling bath was removed, and the reaction mixture was stirred overnight at room temperature. The reaction mixture was subsequently quenched by adding finely crushed ice (50 g), then extracted with Et_2O (3 × 50 mL). The combined organic layer was washed with water (220 mL), isolated and dried over anhydrous $MgSO_4$. After filtration and solvent evaporation under reduced pressure, an oily residue was purified by vacuum distillation thus obtaining amorphous non-crystalline solid (4.94 g, 19.2 mmol, 77%), which solidified after drying under high-vacuum. Attention: since phenothiazine **1** is light-sensitive reagent the above-mentioned reactions have been performed in flasks covered with aluminum folic.

Light-gray crystalline solid; yield 64-77%; Mp 84-85 °C [from hexane/AcOEt (80:20, v/v)] (Lit [2]: mp 78 °C); bp 156-157 °C, p = 0.1 Torr (Lit [3]: bp 192-196 °C, p = 0.3-0.5 Torr); R_f [hexane/AcOEt (80:20, v/v)] 0.20; ^{1}H-NMR ($CDCl_3$, 400 MHz) δ: 1.29 (d, J=6.1 Hz, 3H, CH_3), 2.52 (br. s, 1H, OH), 3.80 (dd, J=13.6, 8.8 Hz, 1H, NCH_2), 3.97 (dd, J=13.6, 3.4 Hz, 1H, NCH_2), 4.14-4.24 (m, 1H, CH), 6.90-7.02 (m, 4H, Ph), 7.15-7.25 (m, 4H, Ph); ^{13}C NMR ($CDCl_3$, 100 MHz) δ: 17.1 (CH$_3$), 52.3 (CH$_2$), 60.6 (CH), 113.3 (2C, Ph), 120.3 (Ph), 123.8 (Ph), 124.6 (2C, Ph), 125.0 (2C, Ph), 142.6 (Ph); HRMS (ESI$^+$, m/z): $[M+H]^+_{calcd}$ = 258.0947, $[M+H]^+_{found}$ = 258.0722; FTIR ν_{max}(neat): 3508, 2970, 2889, 1737, 1590, 1569, 1485, 1451, 1400, 1377, 1342, 1342, 1284, 1247, 1218, 1124, 1110, 1062, 1034, 943, 855, 833, 745, 732, 626, 600, 526; UV/VIS: λ_{max} = 255 nm (EtOH); GC [200-260 (10 °C/min)]: t_R = 7.98 min or [250-260 (10 °C/min)]: t_R = 5.14 min; HPLC [n-hexane/i-PrOH (90:10, v/v); f=0.8 mL/min]: t_R = 8.979 min (R)-**5**, 9.784 min (S)-**5**.

Synthesis of racemic esters (±)-4a-c

To a solution of the 1-(10H-phenothiazin-10-yl)propan-2-ol (±)-**3** (200 mg, 0.78 mmol) in dry CH_2Cl_2 (3 mL), Et_3N (118 mg, 1.17 mmol) and DMAP (9.4 mg, 0.08 mmol) were added. The mixture was cooled to 0-5 °C in ice bath. Next, one of the appropriate acyl chloride (1.17 mmol) was dissolved in dry CH_2Cl_2 (2 mL) and subsequently added dropwise to the reaction mixture. Afterward, the cooling bath was removed, and the resulting mixture was stirred at room temperature overnight, and then quenched with water (5 mL). The water phase was extracted CH_2Cl_2 (3 × 5 mL), and the combined organic layer was washed with saturated solution of $NaHCO_3$ (10 mL), brine (10 mL), and dried (Na_2SO_4). After filtering off the drying agent, and distillation off the residuals of solvent under reduced pressure the crude product was purified by column chromatography on silica gel using hexane/AcOEt (90:10, v/v) as an eluent, thus obtaining corresponding esters (±)-**4a-c** as white crystal or colorless oil with 41-68% yield, respectively.

1-(10*H*-Phenothiazin-10-yl)propan-2-yl acetate (±)-4a:

White crystal; yield 41%; Mp 68-70 °C [from hexane/AcOEt (90:10, v/v)]; R_f [hexane/AcOEt (90:10, v/v)] 0.41; ^{1}H-NMR (CD_3OD, 400 MHz) δ: 1.25 (d, J=6.3 Hz, 3H, CH_3CH), 1.92 (s, 3H, CH_3C=O), 3.88-3.96 (m, 1H, NCH_2), 4.01-4.11 (m, 1H, NCH_2), 5.19-5.28 (m, 1H, CH), 6.90-7.17 (m, 8H, Ph); ^{13}C NMR (CD_3OD, 100 MHz) δ: 18.7 (CH$_3$CH), 22.0 (CH$_3$C=O), 53.3 (NCH$_2$), 69.7 (CH), 118.1 (Ph), 124.7 (Ph), 128.0 (Ph), 129.2 (2C, Ph), 129.3 (2C, Ph), 147.5 (Ph), 173.2 (C=O); HRMS (ESI$^+$, m/z): $[M+H]^+_{calcd}$ = 300.1053, $[M+H]^+_{found}$ = 300.0837; FTIR ν_{max}(neat): 2987, 2929, 1738, 1591, 1570, 1483, 1456, 1366, 1256, 1137, 1125, 1070,

1035, 1012, 956, 940, 898, 867, 754, 598, 466; UV/VIS: λ_{max} = 253 nm (EtOH); GC [200-260 (10 °C/min)]: t_R = 8.90 min or [250-260 (10 °C/min)]: t_R = 5.75 min; HPLC [*n*-hexane/*i*-PrOH (99.5:0.5, v/v); f=0.5 mL/min]: t_R = 19.419 min (*S*)-**4a**, 20.740 min (*R*)-**4a**.

1-(10*H*-Phenothiazin-10-yl)propan-2-yl butanoate (±)-4b:

Colorless oil; yield 68%; R_f [hexane/AcOEt (90:10, v/v)] 0.43; ^{1}H-NMR (CD_3OD, 400 MHz) *δ*: 0.85 (t, *J*=7.5 Hz, 3H, C*H*$_3$), 1.26 (d, *J*=6.3 Hz, 3H, C*H*$_3$CH), 1.52 (sxt, *J*=7.4 Hz, 2H, CH$_3$C*H*$_2$), 2.18 (t, *J*=7.3 Hz, 2H, C*H*$_2$C=O), 3.90-3.98 (m, 1H, NC*H*$_2$), 4.03-4.13 (m, 1H, NC*H*$_2$), 5.18-5.30 (m, 1H, OC*H*), 6.86-7.19 (m, 8H, Ph); ^{13}C NMR (CD_3OD, 100 MHz) *δ*: 14.8 (*C*H$_3$), 18.8 (CH$_3$*C*H$_2$), 20.2 (*C*H$_3$CH), 38.0 (*C*H$_2$C=O), 53.3 (N*C*H$_2$), 69.6 (O*C*H), 118.1 (2C, Ph), 124.7 (2C, Ph), 128.0 (Ph), 129.2 (2C, Ph), 129.3 (2C, Ph), 147.5 (Ph), 175.7 (*C*=O); HRMS (ESI$^+$, m/z): $[M+H]^+_{calcd}$ = 328.1366, $[M+H]^+_{found}$ = 328.1172; FTIR ν_{max}(neat): 2964, 2874, 1728, 1593, 1571, 1456, 1379, 1341, 1285, 1253, 1226, 1178, 1132, 1066, 1037, 954, 931, 863, 744, 627, 601, 526, 469, 444; UV/VIS: λ_{max} = 254 nm (EtOH); GC [200-260 (10 °C/min)]: t_R = 11.18 min or [250-260 (10 °C/min)]: t_R = 8.02 min; HPLC [*n*-hexane/*i*-PrOH (99.5:0.5, v/v); f=0.5 mL/min]: t_R = 14.157 min (*S*)-**4b**, 15.512 min (*R*)-**4b**.

1-(10*H*-Phenothiazin-10-yl)propan-2-yl decanoate (±)-4c:

Colorless oil; yield 44%; R_f [hexane/AcOEt (90:10, v/v)] 0.56; ^{1}H-NMR (CD_3OD, 400 MHz) *δ*: 0.89 (t, *J*=7.2 Hz, 3H, C*H*$_3$), 1.20-1.29 (m, 15H, 6 × C*H*$_2$ and C*H*$_3$CH), 1.42-1.53 (m, 3H, C*H*$_2$CH$_2$C=O), 2.18 (t, *J*=7.5 Hz, 2H, C*H*$_2$C=O), 3.92-3.99 (m, 1H, NC*H*$_2$), 4.03-4.12 (m, 1H, NC*H*$_2$), 5.21-5.31 (m, 1H, OC*H*), 6.86-7.19 (m, 8H, Ph); ^{13}C NMR (CD_3OD, 100 MHz) *δ*: 15.4 (*C*H$_3$), 18.8 (*C*H$_3$CH), 24.6 (CH$_3$*C*H$_2$), 26.8 (*C*H$_2$), 31.0 (*C*H$_2$), 31.3 (*C*H$_2$), 31.3 (*C*H$_2$), 31.4 (*C*H$_2$), 33.9 (*C*H$_2$), 36.2 (*C*H$_2$C=O), 53.4 (N*C*H$_2$), 69.6 (O*C*H), 118.1 (2C, Ph), 124.7 (2C, Ph), 128.0 (Ph), 129.2 (2C, Ph), 129.3 (2C, Ph), 147.5 (Ph), 175.8 (*C*=O); HRMS (ESI$^+$, m/z): $[M+H]^+_{calcd}$ = 412.2305, $[M+H]^+_{found}$ = 412.2048; FTIR ν_{max}(neat): 2923, 2853, 1731, 1593, 1572, 1458, 1377, 1342, 1285, 1254, 1226, 1163, 1133, 1109, 1066, 1038, 965, 929, 865, 746, 726, 469; UV/VIS: λ_{max} = 254 nm (EtOH); HPLC [*n*-hexane/*i*-PrOH (99.5:0.5, v/v); f=0.5 mL/min]: t_R = 9.557 min (*S*)-**4c**, 10.242 min (*R*)-**4c**.

General procedure for analytical-scale kinetic resolution of (±)-3 - enzyme screening

At first, racemic (±)-1-(10*H*-phenothiazin-10-yl)propan-2-ol (±)-**3** (100 mg, 0.39 mmol) was dissolved in MTBE (1 mL). Afterward, the corresponding lipase preparation [20 mg, 20% w/w (catalyst/substrate)] and vinyl acetate (1 g, 11.66 mmol, 1.1 mL) were added in one portion. The reaction mixture was stirred (500 rpm, IKA RCT basic) in a thermo-stated

screw-capped test glass vial (*V* = 4 mL) at 25 °C. The progress of the reaction was monitored by GC and then HPLC analyses in order to obtain information concerning values of %-conversion, % ee and *E*. For HPLC analysis, the samples taken from the reaction mixture (30 μL) were diluted with mobile phase composed of *n*-hexane/*iso*-PrOH (1.5 mL; 3:1, v/v) and filtered before injection. For additional data, see **Figure 1** in the main manuscript.

General procedure for analytical-scale kinetic resolution of (±)-3 - solvent screening

The reaction mixture containing (±)-**3** (100 mg, 0.39 mmol), the appropriate organic solvent (1 mL), vinyl acetate (948 mg, 11 mmol, 1 mL), and respective lipase (Novozym 435 or Lipozyme TL IM) [20 mg, 20% w/w (catalyst/substrate (±)-**3**)] was stirred (500 rpm, IKA RCT basic) in thermo-stated glass vial (*V* = 4 mL) at 25 °C. Further manipulations were carried out by analogy with the previous procedure for the enzyme screening (see section *enzyme screening* above). For additional data, see **Table 1** in the main manuscript.

General procedure for analytical-scale kinetic resolution of (±)-**3 - acyl donor screening**

The reaction mixture containing racemic (±)-**3** (100 mg, 0.39 mmol), MTBE (2 mL), the corresponding acylation agent (3 equiv), and respective lipase (Novozym 435 or Lipozyme TL IM) [20 mg, 20% w/w (catalyst/substrate (±)-**3**)] was stirred (500 rpm, IKA RCT basic) in thermo-stated screw-capped test glass vial (*V* = 4 mL) at 25 °C. Further manipulations were carried out by analogy with the previous enzymatic procedures (see sections *enzyme screening* and *solvent screening* above). For additional data, see **Table 2** in the main manuscript. In order to measure the specific rotations for the enantioenriched fatty acid esters [butyrate (*R*)-(-)-**6b** and decanoate (*R*)-(-)-**6c**], the appropriate reactions with the highest ee-values were stopped, and the enzyme was filtered off, washed with MTBE (10 mL), and after solvent evaporation the crude of the reaction was purified by column chromatography on silica gel by using mixture of hexane/AcOEt (50:10, v/v) as an eluent, affording desired optically active esters (*R*)-(-)-**6b-c**, respectively. Their specific rotations are as follows: (*R*)-(-)-**6b**: $[\alpha]_D^{25}$ = -6.85 (*c* 1.46, CH_2Cl_2, for >99% ee); (*R*)-(-)-**6c**: $[\alpha]_D^{25}$ = -7.26 (*c* 1.86, CH_2Cl_2, for 99% ee).

General procedure for gram-scale (Novozym 435)-catalyzed KR of (±)-3

Racemic (±)-**3** (1 g, 3.89 mmol) was dissolved in MTBE (10 mL). Afterward, suspension of Novozym 435 [200 mg, 20% w/w (catalyst/substrate (±)-**3**)] and vinyl acetate (1 g, 11.66 mmol, 1.1 mL) was added in one portion. The reaction mixture was stirred (500 rpm, IKA RCT basic) in round-bottomed flask (50 mL) equipped with a Teflon-coated magnetic stir bar

(2 cm × 5 mm, 2 g) at 25 °C for 4 days. The progress of the reaction was monitored by GC and chiral HPLC analysis until the required conversion was achieved (ca. 51%). The enzyme was then removed by filtration and washed with separate portions of MTBE (20 mL) and methanol (10 mL), respectively. The volatiles were evaporated from the permeate under reduced pressure, and the crude residue was purified by column chromatography on silica gel (145 g of silica gel was taken) using gradient of hexane/AcOEt (50:10, 20:10, v/v) mixture as an eluent thus affording enantioenriched (*S*)-(+)-1-(10*H*-phenothiazin-10-yl)propan-2-ol (*S*)-(+)-**5** [475 mg, 1.80 mmol, 95% isolated yield, >99% ee, $[\alpha]_D^{25}$ = +12.29 (*c* 1.18, MeOH) or $[\alpha]_D^{23}$ = +39.78 (*c* 1.02, CH_2Cl_2)] and (*R*)-(-)-1-(10*H*-phenothiazin-10-yl)propan-2-yl acetate (*R*)-(-)-**6a** [469 mg, 1.6 mmol, 81% isolated yield, 94% ee, $[\alpha]_D^{25}$ = -4.26 (*c* 0.94, MeOH) or $[\alpha]_D^{24}$ = -8.75 (*c* 1.03, CH_2Cl_2)]. The isolated yield is based on the maximum amount which can be stechiometrically obtained, that is, on a half amount of (±)-**3** used. (The experimental conditions and results of enzymatic resolution reactions are collected in **Table 3** in the main manuscript; physical, spectroscopic and analytical data are identical as for the corresponding racemic standard compounds).

General procedure for gram-scale (Lipozyme TL IM)-catalyzed KR of (±)-3

The reaction was carried out under identical conditions as in the case of gram-scale synthesis catalyzed by Novozym 435 (see above). The crude mixture was analyzed by GC and chiral HPLC analyses and according to their indications the reaction was terminated when conversion reached desired ca. 46% (3 days). The appropriate purification procedure yielded enantioenriched (*S*)-(+)-1-(10*H*-phenothiazin-10-yl)propan-2-ol (*S*)-(+)-**5** [361 mg, 1.40 mmol, 72% isolated yield, 84% ee, $[\alpha]_D^{22}$ = +31.88 (*c* 1.49, CH_2Cl_2)] and (*R*)-(-)-1-(10*H*-phenothiazin-10-yl)propan-2-yl acetate (*R*)-(-)-**6a** [392 mg, 1.31 mmol, 67% isolated yield, >99% ee, $[\alpha]_D^{24}$ = -8.30 (*c* 1.44, CH_2Cl_2)].

General procedure for the determination of the absolute configuration of (*S*)-(+)-5

Esterification of (*S*)-(+)-1-(10*H*-phenothiazin-10-yl)propan-2-ol (*S*)-(+)-5 with (*R*)- or (*S*)-α-methoxy-α-phenylacetic acid

A catalytic amount of DMAP (5 mg) was added to a solution of enantiopure (*S*)-(+)-1-(10*H*-phenothiazin-10-yl)propan-2-ol (*S*)-(+)-**5** (100 mg, 0.39 mmol, >99% ee), (*R*)- or (*S*)-α-methoxy-α-phenylacetic acid (65 mg, 0.39 mmol) as appropriate, and DCC (96 mg, 0.47 mmol) in anhydrous CH_2Cl_2 (3 mL). After 24 h of stirring at room temperature, precipitated dicyclohexylurea was removed by filtration and then the urea cake was rinsed with CH_2Cl_2 (10 mL). The combined CH_2Cl_2 solutions were evaporated to dryness, and the crude product

was purified by preparative layer chromatography using a mixture of hexane/AcOEt (50:10, v/v) as an eluent. Appropriately, the separated fraction was removed from the plate together with SiO_2 gel, placed in a round-bottomed flask, and stirred along with mixture of hexane/AcOEt (100 mL; 1:1, v/v) for over 1 h. Finally, silica gel was filtered off, the fraction was rinsed with AcOEt (50 mL), and the resulting filtrate was evaporated to dryness to afford the corresponding pure product **11** or **12**.

(*R*)-MPA ester 11:

White crystals; yield 93%; Mp 110-111 °C [from hexane/AcOEt]; R_f [hexane/AcOEt (50:10, v/v)] 0.55; ^{1}H NMR (C_6D_6, 400 MHz) δ: 1.05 (d, *J*=6.2 Hz, 3H, C*H*$_3$), 3.32 (s, 3H, OC*H*$_3$), 3.55 (dd, *J*=13.6, 7.2 Hz, 1H, C*H*$_2$N), 3.94 (dd, *J*=13.6, 5.7 Hz, 1H, C*H*$_2$N), 4.76 (s, 1H, C*H*C=O), 5.38-5.47 (m, 1H, CH_3C*H*(O)CH_2), 6.74-6.82 (m, 4H, Ph*H*), 6.98-7.05 (m, 2H, Ph*H*), 7.11-7.29 (m, 5H, Ph*H*), 7.57-7.63 (m, 2H, Ph*H*); ^{13}C NMR (C_6D_6, 100 MHz) δ: 17.4 (*C*H_3), 51.4 (*C*H_2N), 57.3 (O*C*H_3), 68.7 (CH_3*C*H(O)CH_2), 83.0 (*C*HC=O), 116.4 (2C, Ph), 123.2 (2C, Ph), 126.5 (Ph), 127.5 (2C, Ph), 127.5 (2C, Ph), 127.9 (2C, Ph), 128.6 (2C, Ph), 137.3 (Ph), 145.6 (Ph), 170.2 (*C*=O); HRMS calculated for $[M+H]^+$: m/z 406.1477; found: 406.1559.

(*S*)-MPA ester 12:

Yellowish semisolid; yield 92%; R_f [hexane/AcOEt (50:10, v/v)] 0.55; ^{1}H NMR (C_6D_6, 400 MHz) δ: 1.16 (d, *J*=6.4 Hz, 3H, C*H*$_3$), 3.28 (s, 3H, OC*H*$_3$), 3.46 (dd, *J*=13.4, 7.9 Hz, 1H, C*H*$_2$N), 3.92 (dd, *J*=13.5, 5.2 Hz, 1H, C*H*$_2$N), 4.77 (s, 1H, C*H*C=O), 5.50 (dt, *J*=6.5, 1.1 Hz, 1H, CH_3C*H*(O)CH_2), 6.69-6.83 (m, 4H, Ph*H*), 6.94-7.05 (m, 2H, Ph*H*), 7.08-7.31 (m, 5H, Ph*H*), 7.60-7.69 (m, 2H, Ph*H*); ^{13}C NMR (C_6D_6, 100 MHz) δ: ppm 17.6 (*C*H_3), 51.0 (*C*H_2N), 57.2 (O*C*H_3), 68.5 (CH_3*C*H(O)CH_2), 83.1 (*C*HC=O), 116.1 (2C, Ph), 123.2 (2C, Ph), 126.3 (Ph), 127.5 (2C, Ph), 127.5 (2C, Ph), 127.9 (2C, Ph), 128.7 (2C, Ph), 137.3 (Ph), 145.5 (Ph), 170.2 (*C*=O); HRMS calculated for $[M+H]^+$: m/z 406.1477; found: 406.1559.

X-ray crystallography

Crystal structure determination of (*S*)-(+)-5

Colorless single crystals, suitable for X-ray diffraction studies, were grown by slow diffusion of hexane (0.5 mL) into a concentrated solution of the (*S*)-(+)-**5** (50 mg) in AcOEt (1 mL) at 0-5 °C. Selected crystal of dimensions 0.53×0.22×0.22 mm^3 was mounted in inert oil and transferred to the cold gas stream of the diffractometer. Diffraction data were measured at 120.0(2) K with mirror monochromated Cu*K*α radiation on an Oxford Diffraction κ-CCD Gemini A Ultra diffractometer. Cell refinement and data collection as well as data reduction and analysis were performed with the $CRYSALIS^{PRO}$ Software [4]. The structure was solved by direct methods using the SHELXS-97 structure solution program and refined by full-matrix least-squares against F^2 with SHELXL-2013 [5] and OLEX2 [6] programs. All non-hydrogen atoms were refined with anisotropic displacement parameters. Hydrogen atoms attached to carbon atoms were added to the structure model at geometrically idealized coordinates and refined as riding atoms with Uiso(H) = 1.2Ueq(CH and CH_2) or Uiso(H) = 1.5Ueq(CH_3). The hydrogen atom of the hydroxyl group was refined with a restraint of O–H = 0.84(2)Å. An absolute (*S*)-configuration for the compound molecule (+)-**5** was successfully determined using anomalous dispersion effects. Flack parameter [7] calculated from 991 selected quotients (Parsons' method) [8] equals 0.007(4). Further analysis of the absolute structure was performed using likelihood methods with PLATON [9]. A total of 999 Bijvoet pairs (coverage of 1.00) were included in the calculations. The resulting value of the Hooft parameter [10] was 0.002(4), with a P3 probability for an inverted structure smaller than 1×10^{-100}. These results indicated that the absolute structure has been correctly assigned. **Crystal Data** for (*S*)-(+)-**5**, $C_{15}H_{15}NOS$ (M =257.34): monoclinic, space group $P2_1$ (no. 4), a = 8.24979(8) Å, b = 5.57607(4) Å, c = 13.88078(11) Å, β = 96.1455(8)°, V = 634.865(10) $Å^3$, Z = 2, T = 120.0(1) K, μ(CuKα) = 2.144 mm^{-1}, $Dcalc$ = 1.346 g/cm^3, 35287 reflections measured (6.4 ≤ 2Θ ≤ 133.5), 2254 unique (R_{int} = 0.0268, R_{sigma} = 0.0100) which were used in all calculations. The final R_1 was 0.0214 ($I > 2\sigma(I)$) and wR_2 was 0.0584 (all data). CCDC-1018655 contains the supplementary crystallographic data for compound (*S*)-(+)-**5**. This can be obtained free of charge on application to CDC, 12 Union Road, Cambridge CB21EZ, UK [Fax: (+44)1223-336-033; email: deposit@ccdc.cam.ac.uk]. ORTEP drawing was made using Ortep3 for Windows [11].

Table 1 Crystal data and structure refinement parameters for (*S*)-(+)-**5**.

Compound	(*S*)-(+)-**5**
Chemical formula	$C_{15}H_{15}NOS$
M /g·mol^{-1}	257.34
Crystal size /mm^3	0.532×0.222×0.218
T /K	120.0(1)
Crystal system	monoclinic
Space group	$P2_1$
a /Å	8.24979(8)
b /Å	5.57607(4)
c /Å	13.88078(11)
β /°	96.1455(8)
V /Å^3	634.865(10)
Z	2
D_{calc} /g·cm^{-3}	1.346
Radiation, λ/ Å	CuK$_\alpha$ (λ = 1.5418)
μ /mm^{-1}	2.144
F(000)	272.0
2Θ Range /°	6.4–133.5°
Reflections collected	35287
Independent refln.	2254
R_{int}	0.0268
Parameters/restraints	168/2
S (F^2)[a]	1.078
R1, wR2 (I>2$\sigma(I)$)[b]	0.0214, 0.0584
R1, wR2 (all data)	0.0215, 0. 0584
$\Delta\rho_{min/max}$/eÅ^{-3}	+0.14/−0.14
Flack parameter	0.007(4)

[a] Goodness-of-fit $S=\{\Sigma[w(F_o^2-F_c^2)^2]/(n-p)\}^{1/2}$ where n is the reflections number and p is the parameters number; [b] $R1=\Sigma||F_o|-|F_c||/\Sigma|F_o|$, $wR2=\{\Sigma[w(F_o^2-F_c^2)^2]/\Sigma[w(F_o^2)^2]\}^{1/2}$.

Chemical base-mediated methanolysis of (*R*)-(-)-1-(10*H*-phenothiazin-10-yl)propan-2-yl acetate (*R*)-(-)-6a as the method for the synthesis of (*R*)-(-)-7

To a solution of enantiopure (*R*)-(-)-**6a** (200 mg, 0.67 mmol, >99% ee) in MeOH (2.5 mL) a solution of NaOH (29 mg, 0.73 mmol) in MeOH (5 mL) was added. The mixture was stirred at room temperature for 1 h until complete consumption of the starting material (TLC). Then MeOH was evaporated under reduced pressure, and the resulting oil was suspended in H_2O (10 mL), and the water phase was back-extracted with CH_2Cl_2 (3 × 10 mL). The organic layer was rinsed with H_2O (20 mL), combined and dried over anhydrous Mg_2SO_4. After solvent evaporation, the reaction crude was purified by column chromatography on silica gel using

hexane/AcOEt (80:20, v/v) mixture as an eluting system to afford alcohol (*R*)-(-)-**7** [130 mg; 0.51 mmol; 76%; 98% ee, $[\alpha]_D^{22}$ = -35.5 (*c* 1.73, CH_2Cl_2)].

Synthesis of (2*R*)-(+)- and (2*S*)-(−)-10-(2-bromopropyl)-10*H*-phenothiazine (*R*)-(+)-8 and (*S*)-(-)-8

Method A: Enantiopure (*S*)-(+)-1-(10*H*-phenothiazin-10-yl)propan-2-ol (*S*)-(+)-**5** (660 mg, 2.6 mmol, >99% ee) was dissolved in CH_2Cl_2 (4 mL) and cooled to 0-5 °C. Next, PBr_3 (690 mg, 2.6 mmol, 0.24 mL) was injected and after 30 min ice-bath was removed, and the reaction mixture was further stirred at room temperature for additional 2 h. The reaction was terminated by addition of saturated $NaHCO_3$ (5 mL), and the water phase was back-extracted using CH_2Cl_2 (3 × 5 mL). Excess solvent and other volatile reaction components were removed under reduced pressure, and the crude product was re-crystallized from EtOH (8 mL) to afford optically active (2*R*)-(+)-10-(2-bromopropyl)-10*H*-phenothiazine (*R*)-(+)-**8** as white crystals (220 mg, 0.69 mmol, 27%, >99% ee). The same procedure was conducted toward (*R*)-(-)-**7** [1.15 g, 4.5 mmol, 98% ee, $[\alpha]_D^{23}$ = +4.55 (*c* 0.99, CH_2Cl_2)] and allowed to yield (*S*)-(-)-**8** [392 mg, 1.2 mmol, 27%, 96% ee, $[\alpha]_D^{21}$ = -3.86 (*c* 1.16, CH_2Cl_2)].

Method B: To a solution of (*S*)-(+)-**5** (1 g, 3.89 mmol, >99% ee) in anhydrous THF (20 mL), triphenylphosphine (2.45 g, 9.33 mmol) and pyridine (338 mg, 4.27 mmol, 0.34 mL) were added and cooled to 0-5 °C. Next, solution of tetrabromomethane (1.55 g, 4.66 mmol) in anhydrous THF (10 mL) was added dropwise over 10 min with vigorous stirring. Upon addition of the tetrabromomethane, the colorless solution turned a pale brown color and was stirred for an additional 12 h at room temperature. The solvent excess was partially condensed under vacuum, and the crude product was filtered over a short pad of silica gel (CH_2Cl_2). The permeate was evaporated to dryness and the remaining solid was purified by recrystallization from EtOH (20 mL) affording (*R*)-(+)-**8** (958 mg, 2.99 mmol, 77%, 23% ee) as white crystals. White crystals; Mp 126-127 °C (from EtOH) [Lit [3]: mp 125-126 °C (from EtOH)]; R_f [hexane/AcOEt (90:10, v/v)] 0.69; ^{1}H NMR (C_6D_6, 400 MHz) δ: 1.41 (d, *J*=6.6 Hz, 3H, C*H*$_3$), 3.64-3.74 (m, 1H, C*H*), 4.02-4.15 (m, 2H, C*H*$_2$), 6.42 (dd, *J*=8.1, 0.9 Hz, 2H, Ph*H*), 6.61-6.69 (m, 2H, Ph*H*), 6.78-6.86 (m, 2H, Ph*H*), 6.98-7.05 (m, 2H, Ph*H*); ^{13}C NMR (C_6D_6, 100 MHz) δ: 23.1 (*C*H$_3$), 45.1 (*C*H$_2$), 55.6 (*C*H), 116.0 (2C, Ph), 123.3 (Ph), 126.5 (Ph), 127.6 (2C, Ph), 128.1 (2C, Ph), 145.0 (Ph); HRMS calculated for $[M+H]^+$: m/z 320.0103; found: 320.0286; FTIR ν_{max}(neat): 1590, 1569, 1483, 1454, 1441, 1376, 1333, 1284, 1250, 1229, 1186, 1124, 1102, 1049, 1032, 1005, 932, 855, 744, 727, 648, 575, 535, 502, 438; UV/VIS: λ_{max} = 255 nm (EtOH); HPLC [*n*-hexane/*i*-PrOH (99.9:0.1, v/v); f=0.5 mL/min]: t_R = 14.769 min (*R*)-**8**,

15.736 min (*S*)-**8** or HPLC [*n*-hexane/*i*-PrOH (99.9:0.1, v/v); f=0.3 mL/min]: t_R = 23.165 min (*R*)-**8**, 24.654 min (*S*)-**8**.

Synthesis of *N,N*-dimethyl-1-(10*H*-phenothiazin-10-yl)propan-2-amine (promethazine) (*R*)-(+)-9 and (*S*)-(-)-9 base

Method A (single inversion): The solution of respective optically active bromide (*R*)-(+)-**8** (>99% ee) or (*S*)-(-)-**8** (96% ee) (150 mg, 0.47 mmol) and dimethylamine (5.85 mL of 2 M solution in THF, 11.7 mmol) in dry $PhCH_3$ (15 mL) was placed in glass sealed tube (*V* = 25 mL) and stirred for 7 days at 140 °C (temperature of oil bath). The reaction was stopped by evaporation of residual volatiles of amine and subsequent addition of saturated $NaHCO_3$ (20 mL). Next, water phase was extracted with CH_2Cl_2 (3 × 15 mL), the organic solvent was directly evaporated under reduced pressure and the reaction crude purified by preparative layer chromatography (PLC) on plates coated with unmodified silica gel matrix using eluent system composed of $PhCH_3$/AcOEt/Et_3N (33:67:10, v/v/v) mixture, affording desired optically active amine (*S*)-(-)-**9** [62 mg, 0.22 mmol, 47%, 84% ee, $[\alpha]_D^{21}$ = -1.94 (*c* 0.78, CH_2Cl_2)] or (*R*)-(+)-**9** [78 mg, 0.27 mmol, 59%, 92% ee, $[\alpha]_D^{22}$ = +1.45 (*c* 1.04, CH_2Cl_2)] as a clear amber oil, respectively.

Method B (double inversion): The solution of respective optically active bromide (*R*)-(+)-**8** (>99% ee) or (*S*)-(-)-**8** (96% ee) (180 mg, 0.56 mmol) and dimethylamine (1.41 mL of 2 M solution in THF, 2.80 mmol) in MeOH (4 mL) was placed in glass sealed tube (*V* = 25 mL), and stirred for 4 days at 90 °C (temperature of oil bath). The reaction was stopped by evaporation of residual volatiles of amine and subsequent addition of saturated $NaHCO_3$ (20 mL). Next, water phase was extracted with CH_2Cl_2 (3 × 15 mL), the organic solvent was directly evaporated under reduced pressure and the reaction crude purified by preparative layer chromatography (PLC) on plates coated with unmodified silica gel using eluent system composed of $PhCH_3$/AcOEt/Et_3N (33:67:10, v/v/v) mixture yielding desired optically active (*R*)-(+)-**9** [73 mg, 0.26 mmol, 46%, 97% ee, $[\alpha]_D^{21}$ = +1.64 (*c* 0.61, CH_2Cl_2)] or (*S*)-(-)-**9** [41 mg, 0.13 mmol, 23%, 94% ee, $[\alpha]_D^{21}$ = -1.40 (*c* 1.01, CH_2Cl_2)] as a clear amber oils.

Amber oil; R_f [$PhCH_3$/AcOEt/Et_3N (33:67:10, v/v/v)] 0.15; ^{1}H NMR (C_6D_6, 400 MHz) δ: 0.87 (d, *J*=6.6 Hz, 3H, C*H*$_3$), 2.06 (s, 6H, NC*H*$_3$), 2.95-3.07 (m, 1H, C*H*), 3.45 (dd, *J*=13.3, 8.7 Hz, 1H, C*H*$_2$), 3.84 (dd, *J*=13.3, 4.1 Hz, 1H, C*H*$_3$), 6.63-6.76 (m, 4H, Ph*H*), 6.88-6.99 (m, 2H, Ph*H*), 7.05-7.12 (m, 2H, Ph*H*); ^{1}H NMR (C_6D_6, 500 MHz) δ: 0.89 (d, *J*=6.8 Hz, 3H, C*H*$_3$), 2.08 (s, 6H, NC*H*$_3$), 2.97-3.08 (m, 1H, C*H*), 3.46 (dd, *J*=13.2, 8.8 Hz, 1H, C*H*$_2$), 3.85 (dd, *J*=13.2, 4.4 Hz, 1H, C*H*$_2$), 6.67-6.76 (m, 4H, Ph*H*), 6.91-6.97 (m, 2H, Ph*H*), 7.07-7.12 (m, 2H, Ph*H*); ^{13}C NMR (C_6D_6, 100 MHz) δ: 11.9 (*C*H$_3$), 40.9 (2C, *C*H$_3$), 51.5 (*C*H$_2$), 56.1 (*C*H),

116.2 (Ph), 122.8 (Ph), 126.4 (Ph), 127.4 (Ph), 146.1 (Ph); HRMS calculated for $[M+H]^+$: m/z 285.1425; found: 285.1078; FTIR ν_{max}(neat): 3062, 2967, 2932, 2866, 2823, 2774, 1593, 1571, 1456, 1356, 1335, 1285, 1250, 1227, 1188, 1159, 1128, 1105, 1037, 974, 928, 852, 745, 723, 695, 620, 604, 541, 495, 432; UV/VIS: λ_{max} = 255 nm (EtOH); GC [200 °C (isothermally)]: t_R = 30.99 min; HPLC [*n*-hexane/*tert*-BuOH/Et_3N (96.5:3:0.5, v/v/v); f=1.0 mL/min]: t_R = 17.076 min (*R*)-**9**, 22.468 min (*S*)-**9** or [*n*-hexane/EtOH (95:5, v/v); f=0.5 mL/min]: t_R = 14.157 min (*R*)-**9**, 17.476 min (*S*)-**9**.

Synthesis of *N,N*-diethyl-1-(10*H*-phenothiazin-10-yl)propan-2-amine (ethopropazine) (*S*)-(-)-10 and (*R*)-(+)-10 base

Method A (single inversion): The solution of respective optically active bromide (*R*)-(+)-**8** (>99% ee) or (*S*)-(-)-**8** (96% ee) (150 mg, 0.47 mmol) and diethylamine (2.43 mL, 23.4 mmol) in dry $PhCH_3$ (15 mL) was placed in glass sealed tube (*V* = 25 mL), and stirred for 7 days at 140 °C (temperature of oil bath). The rest of the procedure is essentially the same as for the Method A synthesis of promethazine **9** enantiomers what allowed to obtain (*S*)-(-)-**10** [55 mg, 0.18 mmol, 38%, 90% ee, $[\alpha]_D^{25}$ = -3.94 (*c* 1.02, CH_2Cl_2)] or (*R*)-(+)-**10** [34 mg, 0.11 mmol, 23%, 93% ee, $[\alpha]_D^{22}$ = +9.68 (*c* 1.24, CH_2Cl_2)] as a clear amber oils, respectively.

Method B (double inversion): A glass sealed tube (*V* = 25 mL) was charged with a solution of respective optically active bromide (*R*)-(+)-**8** (>99% ee) or (*S*)-(-)-**8** (96% ee) (180 mg, 0.56 mmol) and diethylamine (1.16 mL, 11.20 mmol) in MeOH (4 mL), and the mixture was stirred for 4 days at 90 °C (temperature of oil bath). The rest of the procedure is essentially the same as for the Method B synthesis of promethazine **9** enantiomers what allowed to obtain (*R*)-(+)-**10** [59 mg, 0.21 mmol, 38%, 98% ee, $[\alpha]_D^{23}$ = +10.60 (*c* 0.42, CH_2Cl_2)] or (*S*)-(-)-**10** [55 mg, 0.18 mmol, 31%, 96% ee, $[\alpha]_D^{24}$ = -9.80 (*c* 0.51, CH_2Cl_2)] as a clear amber oils, respectively.

Amber oil; R_f [$PhCH_3$/AcOEt/Et_3N (33:67:10, v/v/v)] 0.22; ^{1}H NMR (CD_3OD, 400 MHz) δ: 0.95-1.04 (m, 9H, CH_3), 2.43-2.54 (m, 2H, CH_2CH_3), 2.56-2.67 (m, 2H, CH_2CH_3), 3.28-3.31 (m, 1H, C*H*), 3.72 (dd, *J*=13.6, 8.6 Hz, 1H, CH_2), 4.03 (dd, *J*=13.6, 4.5 Hz, 1H, CH_2), 6.86-6.99 (m, 4H, Ph*H*), 7.07-7.18 (m, 4H, Ph*H*) or ^{1}H NMR (C_6D_6, 400 MHz) δ: 0.88 (d, *J*=6.6 Hz, 3H, CH_3), 0.93 (t, *J*=7.2, 6H, CH_3), 2.18 (dq, *J*=13.2, 6.7 Hz, 2H, CH_2CH_3), 2.29-2.40 (m, 2H, CH_2CH_3), 3.26 (ddd, *J*=8.7, 6.5, 4.6 Hz, 1H, C*H*), 3.51 (dd, *J*=13.2, 8.8 Hz, 1H, CH_2), 3.80 (dd, *J*=13.2, 4.4 Hz, 1H, CH_2), 6.66-6.77 (m, 4H, Ph*H*), 6.91-6.97 (m, 2H, Ph*H*), 7.06-7.10 (m, 2H, Ph*H*); ^{13}C NMR (CD_3OD, 100 MHz) δ: 14.0 (CH_3), 14.8 (2C, CH_2CH_3), 45.6 (2C, CH_2CH_3), 52.3 (CH_2), 53.3 (*C*H), 118.0 (2C, Ph), 124.5 (2C, Ph), 128.0 (Ph), 129.2 (2C, Ph), 129.3 (2C, Ph), 147.7 (Ph); HRMS calculated for $[M+H]^+$: m/z 313.1738; found:

313.1496; FTIR ν_{max}(neat): 3062, 2966, 2928, 2869, 2809, 1593, 1571, 1456, 1361, 1334, 1314, 1285, 1249, 1227, 1205, 1178, 1128, 1104, 1067, 1037, 927, 868, 848, 743, 727, 696, 676, 624, 605, 539, 509, 445; UV/VIS: λ_{max} = 255 nm (EtOH); GC [200 °C (isothermally)]: t_R = 36.99 min; HPLC [*n*-hexane/*tert*-BuOH/Et_3N (96:3.5:0.5, v/v/v); f=0.9 mL/min]: t_R = 17.785 min (*S*)-**10**, 19.315 min (*R*)-**10**.

10-[(2*S*)-2-Methoxypropyl]-10*H*-phenothiazine (*S*)-16:

Amber oil; R_f [$PhCH_3$/AcOEt (90:10, v/v)] 0.64; ^1H NMR (C_6D_6, 400 MHz) δ: 1.04 (d, *J*=6.1 Hz, 3H, CH_3), 3.09 (s, 3H, OCH_3), 3.50 (dd, *J*=13.4, 6.9 Hz, 1H, one of CH_2), 3.54-3.63 (m, 1H, C*H*), 3.87 (dd, *J*=13.4, 4.9 Hz, 1H, one of CH_3), 6.60-6.71 (m, 4H, Ph*H*), 6.86-6.93 (m, 2H, Ph*H*), 7.04-7.08 (m, 2H, Ph*H*); ^{13}C NMR (C_6D_6, 100 MHz) δ: 17.8 (*C*H_3), 53.4 (*C*H_2), 56.7 (O*C*H_3), 73.9 (*C*H), 116.1 (2C, Ph), 122.9 (2C, Ph), 126.2 (Ph), 127.4 (2C, Ph), 127.9 (2C, Ph), 146.0 (Ph); HRMS calculated for $[M+H]^+$: m/z 272.1109; found: 272.0670.

Table S1. Analytical separation conditions of different compounds enantiomers by Chiralcel OD-H and Chiralcel OJ chiral columns (Daicel)

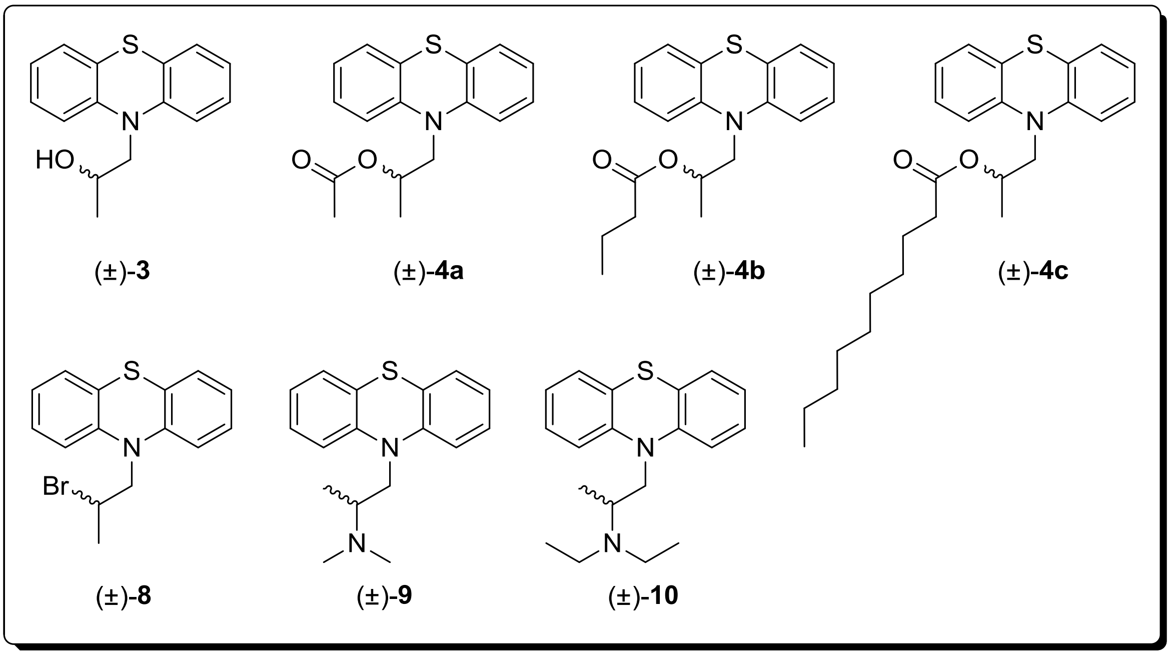

Compound[a,b]	Ratio of *n*-hexane to 2-PrOH [v/v]	Elution velocity [mL/min]	Retention time (min)
(±)-**3**	90:10	0.8	8.979 (*R*) and 9.784 (*S*)
(±)-**4a**	99.5:0.5	0.5	19.419 (*S*) and 20.740 (*R*)
(±)-**3** + (±)-**4a**	0-20 min: 99.5:0.5<br>20-22 min: gradient<br>22-45 min: 90:10	0-20 min: 0.5<br>20-22 min: gradient<br>22-45 min: 0.8	(±)-**4a**: 18.986 (*S*) and 20.188 (*R*)<br>(±)-**3**: 33.078 (*R*) and 33.900 (*S*)
(±)-**4b**	99.5:0.5	0.5	14.167 (*S*) and 16.612 (*R*)
(±)-**3** + (±)-**4b**	0-16 min: 99.5:0.5<br>16-18 min: gradient<br>18-41 min: 90:10	0-20 min: 0.5<br>16-18 min: gradient<br>18-41 min: 0.8	(±)-**4b**: 13.998 (*S*) and 16.364 (*R*)<br>(±)-**3**: 29.341 (*R*) and 30.165 (*S*)
(±)-**4c**	99.5:0.5	0.5	9.557 (*S*) and 10.242 (*R*)
(±)-**3** + (±)-**4c**	0-16 min: 99.5:0.5<br>16-18 min: gradient<br>18-41 min: 90:10	0-20 min: 0.5<br>16-18 min: gradient<br>18-41 min: 0.8	(±)-**4c**: 9.559 (*S*) and 10.244 (*R*)<br>(±)-**3**: 29.296 (*R*) and 29.089 (*S*)
(±)-**8**	99.9:0.1<br>[99.9:0.1]	0.5<br>[0.3]	14.769 (*R*) and 16.736 (*S*)<br>[23.165 (*R*) and 24.654 (*S*)]
**Compound[a,c]**	**Ratio of *n*-hexane to abs. EtOH [v/v]**	**Elution velocity [mL/min]**	**Retention time (min)**
(±)-**9**	95:5.0	0.5	14.157 (*R*) and 17.476 (*S*)
**Compound[a,d]**	**Ratio of *n*-hexane to *t*-BuOH to Et_3N [v/v/v]**	**Elution velocity [mL/min]**	**Retention time (min)**
(±)-**9**	96.5:3.0:0.5	1.0	17.076 (*R*) and 22.468 (*S*)
(±)-**10**	96:3.5:0.5	0.9	17.785 (*S*) and 19.315 (*R*)

[a] The samples were carried out at 254 nm and at 30 °C.
[b] Chiralcel OD-H column was used.
[c] Chiralcel OJ column was used.
[c] Tandemic system composed of two serially coupled Chiralcel OJ-Chiralcel OJ columns was used.

<u>Analytical data for 1-(10*H*-phenothiazin-10-yl)propan-2-ol (±)-3</u>:

HPLC analytical separation for both enantiomers of (±)-3

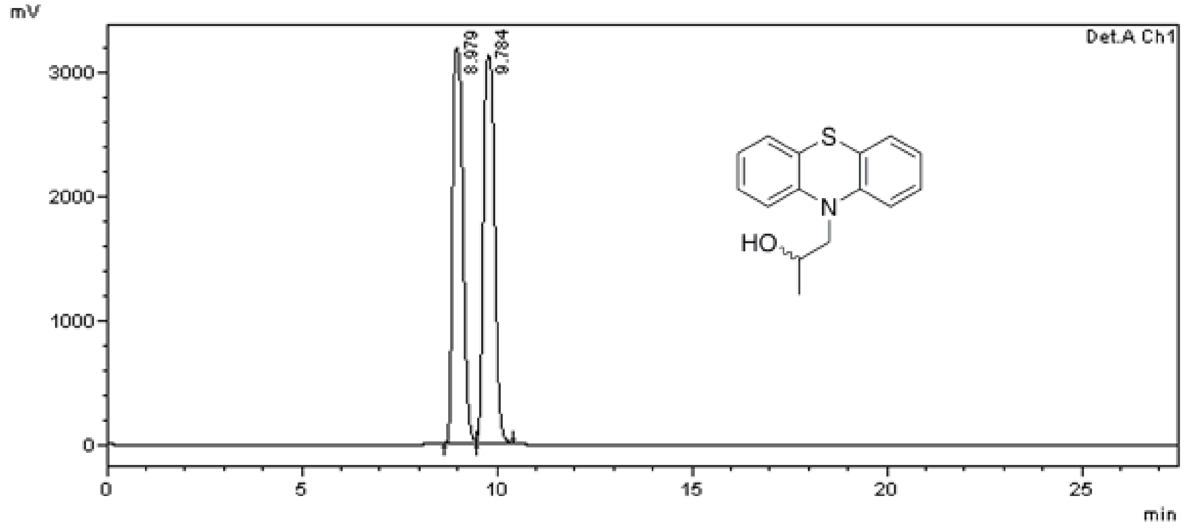

HPLC analytical separation for mixture of both enantiomers of (±)-3 and (±)-4a

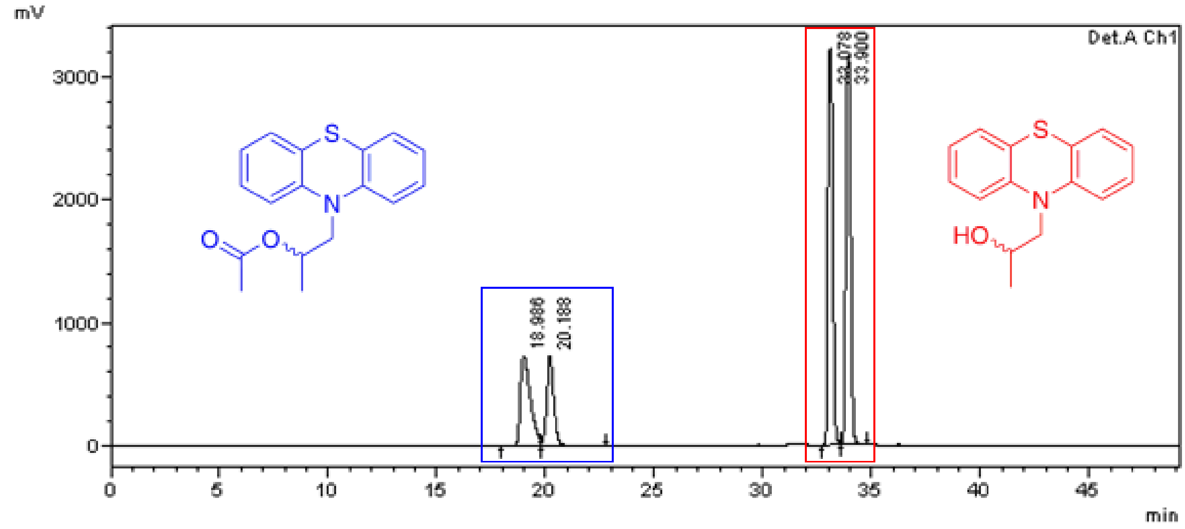

(*S*)-(+)-5 in >99% ee

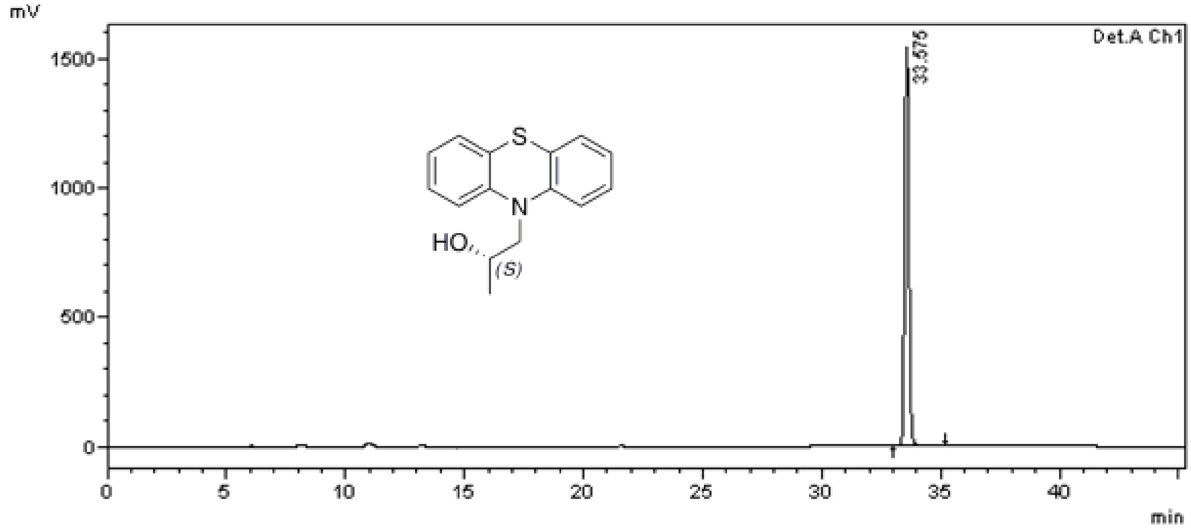

(*R*)-(-)-7 in 98% ee [after NaOH-mediated methanolysis of enantiopure (*R*)-(-)-6a]

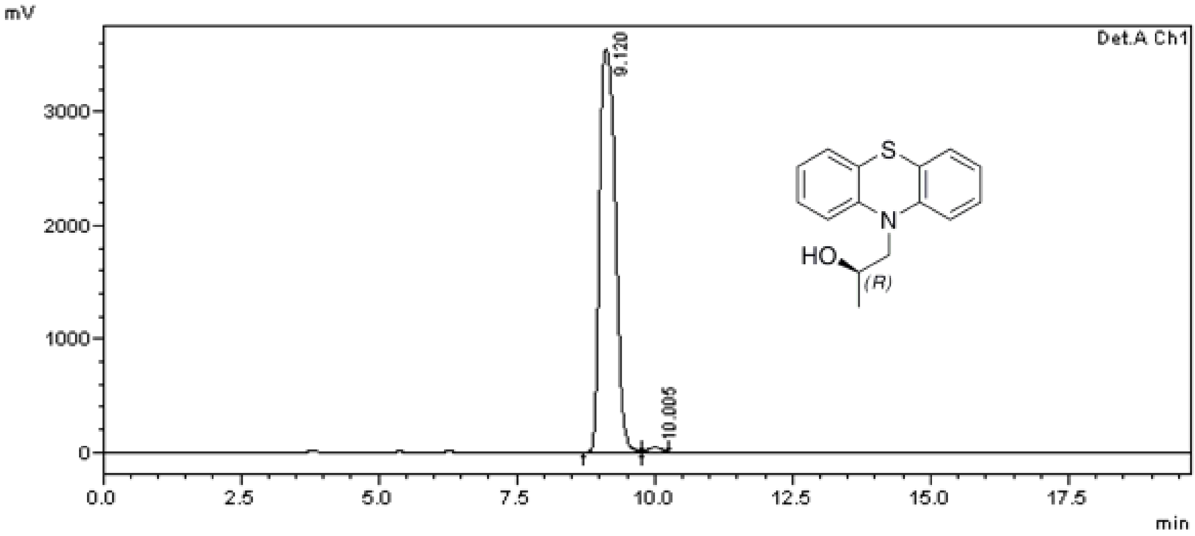

<u>Analytical data for 1-(10*H*-phenothiazin-10-yl)propan-2-yl acetate (±)-4a</u>:

HPLC analytical separation for both enantiomers of (±)-4a

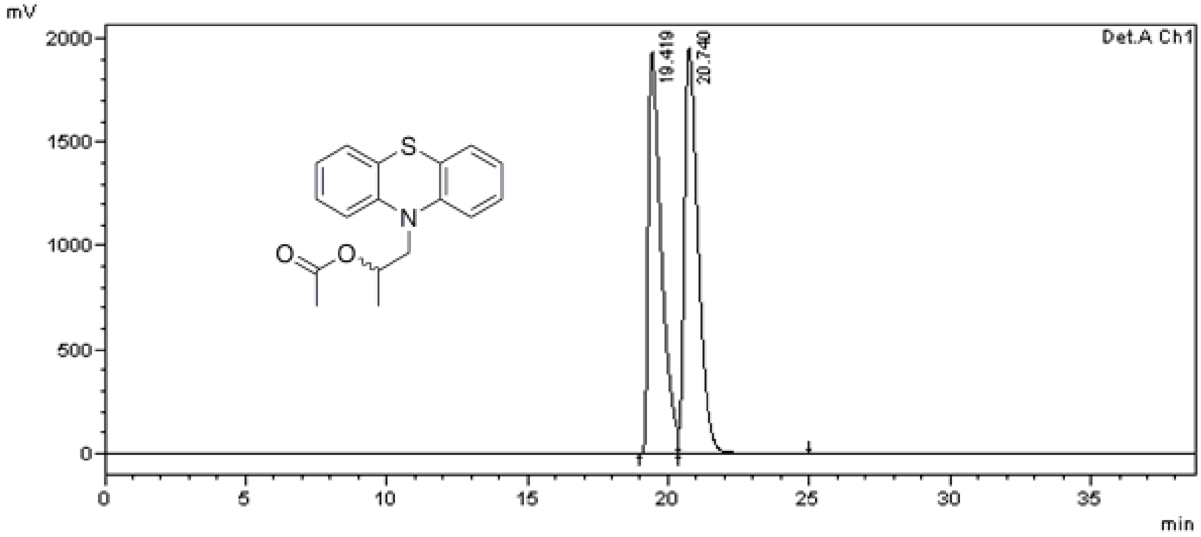

(*R*)-(-)-6a in >99% ee

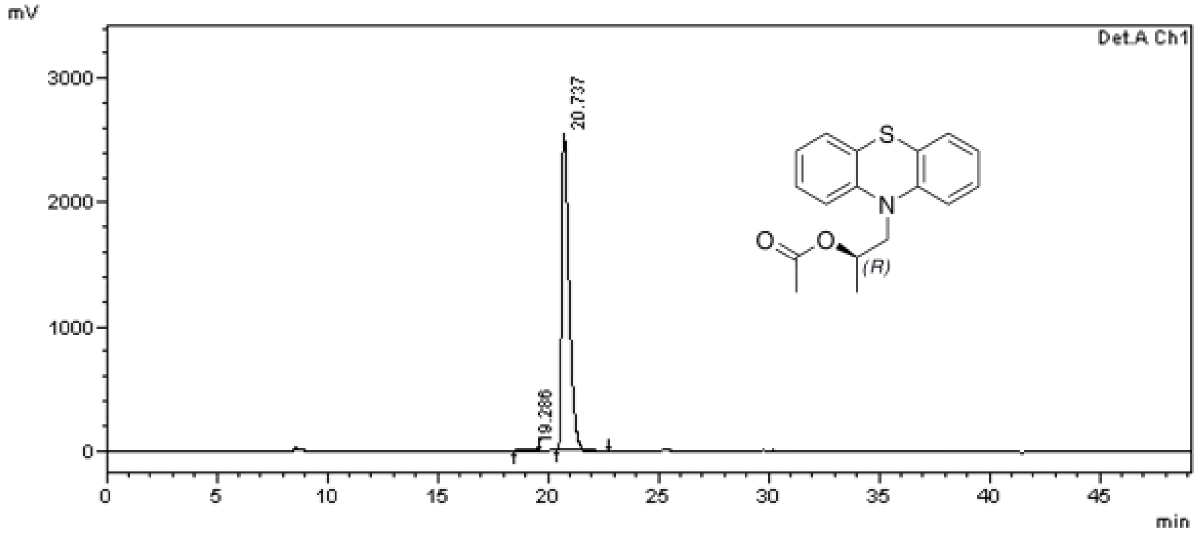

Analytical data for 1-(10*H*-phenothiazin-10-yl)propan-2-yl butanoate (±)-4b:

HPLC analytical separation for both enantiomers of (±)-4b

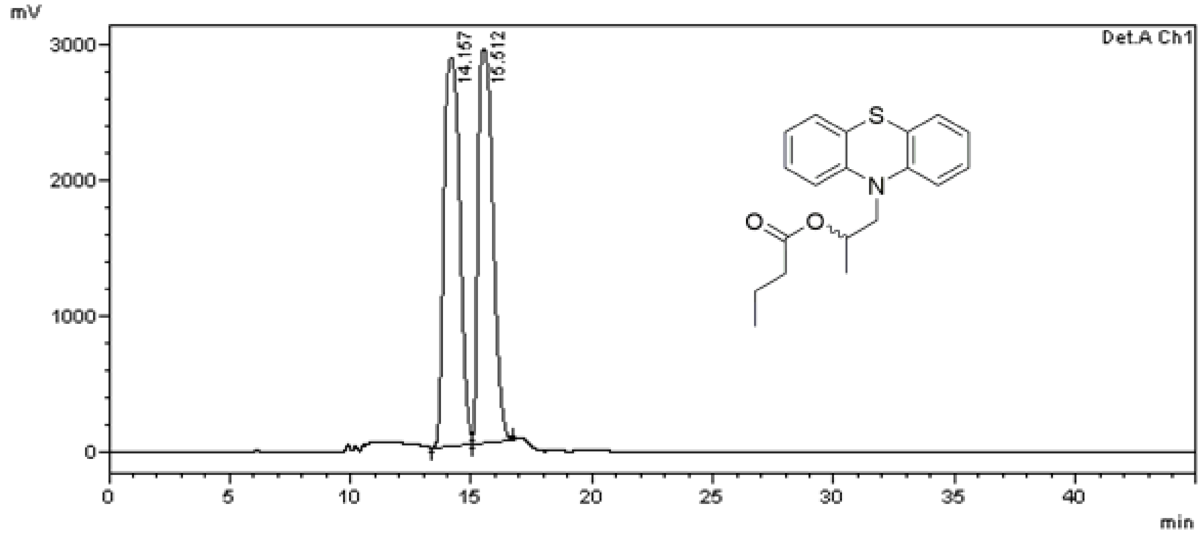

HPLC analytical separation for mixture of both enantiomers of (±)-3 and (±)-4b

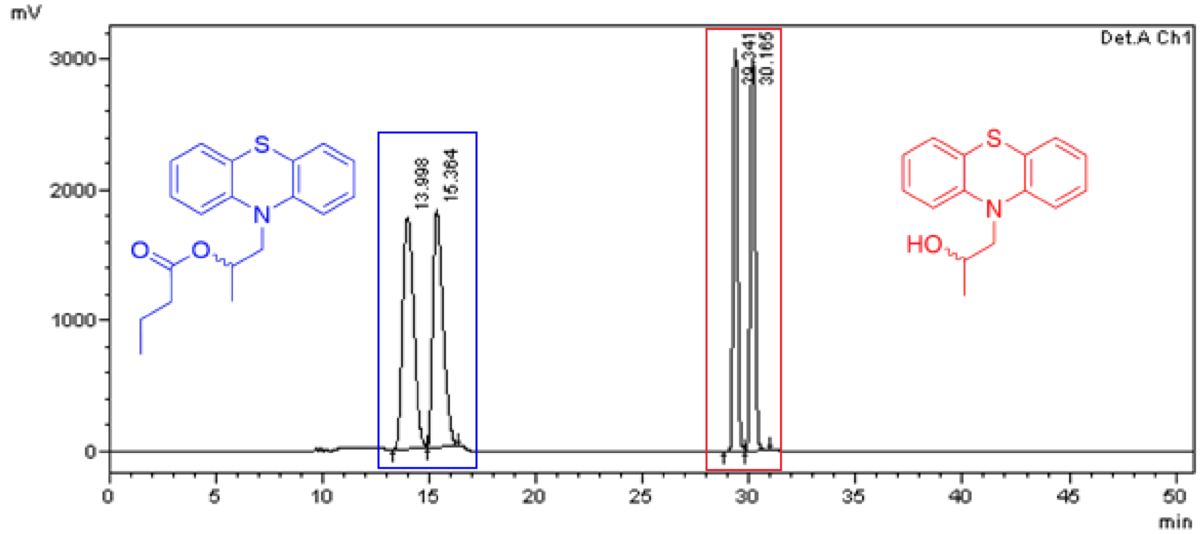

(*R*)-(-)-6b in >99% ee

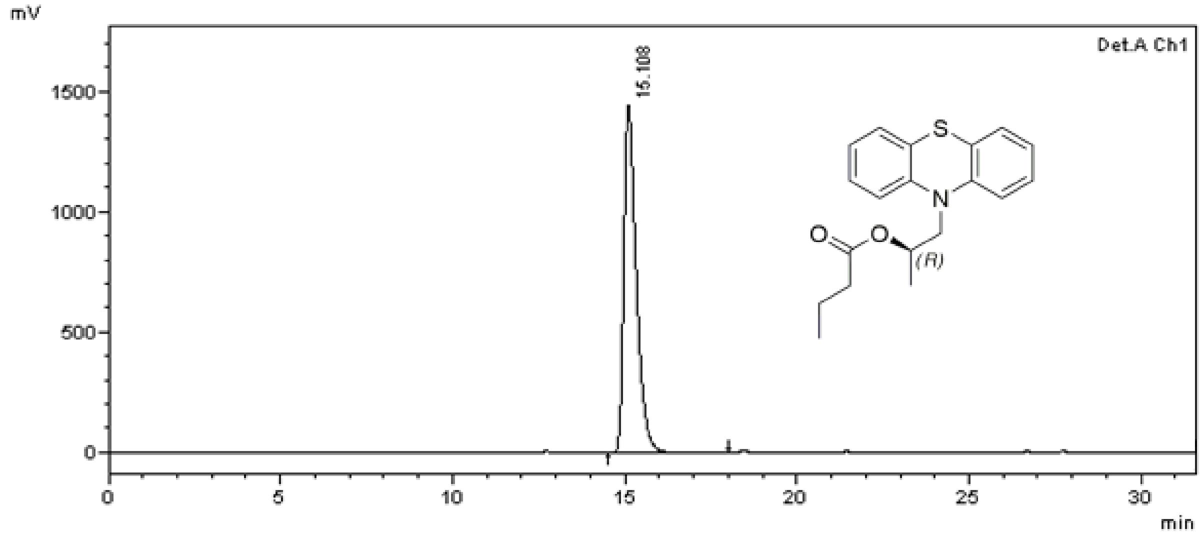

<u>Analytical data for 1-(10*H*-phenothiazin-10-yl)propan-2-yl decanoate (±)-4c</u>:

HPLC analytical separation for both enantiomers of (±)-4c

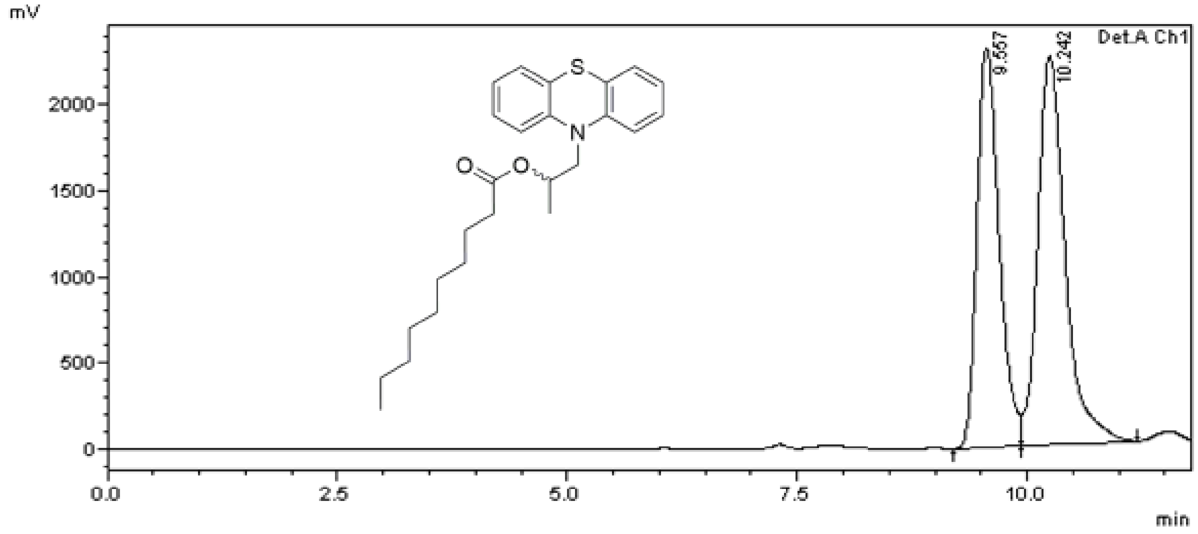

HPLC analytical separation for mixture of both enantiomers of (±)-3 and (±)-4c

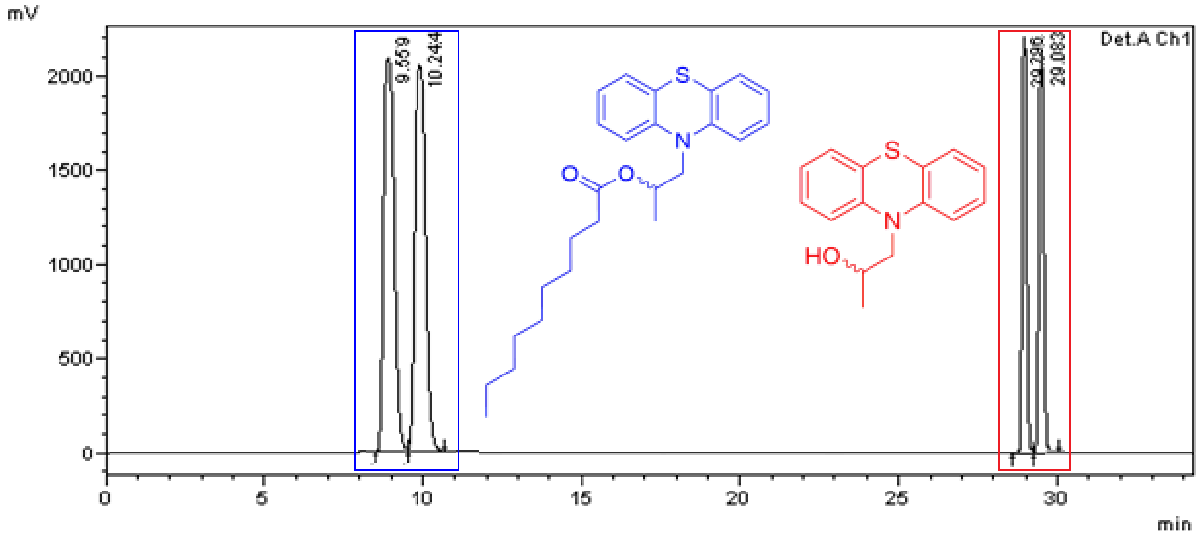

(*R*)-(-)-6c in >99% ee

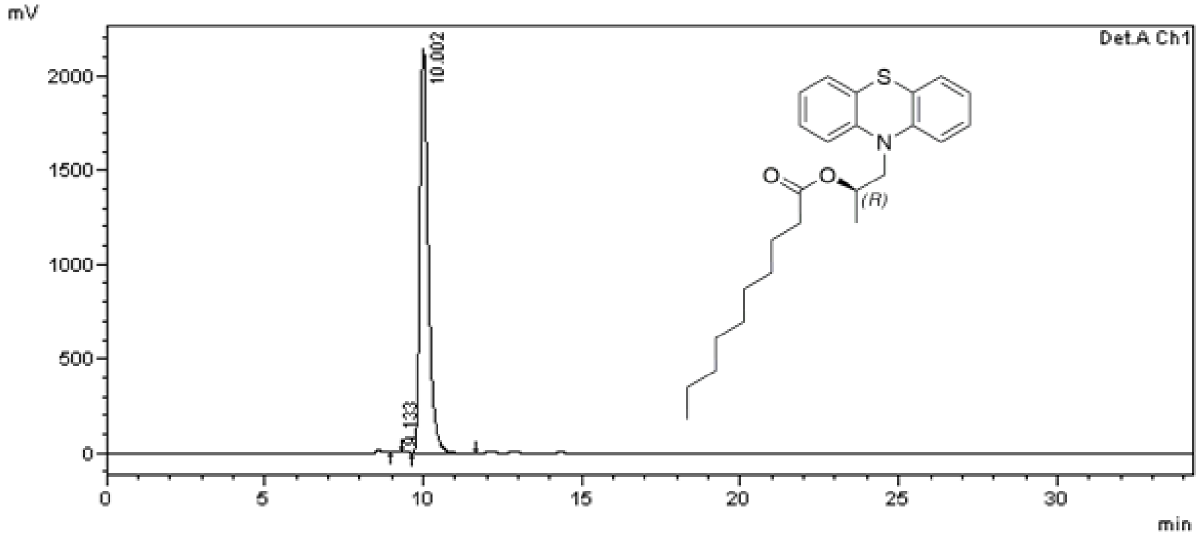

<u>Analytical data for 10-(2-bromopropyl)-10*H*-phenothiazine (±)-8</u>:

HPLC analytical separation for both enantiomers of (±)-8

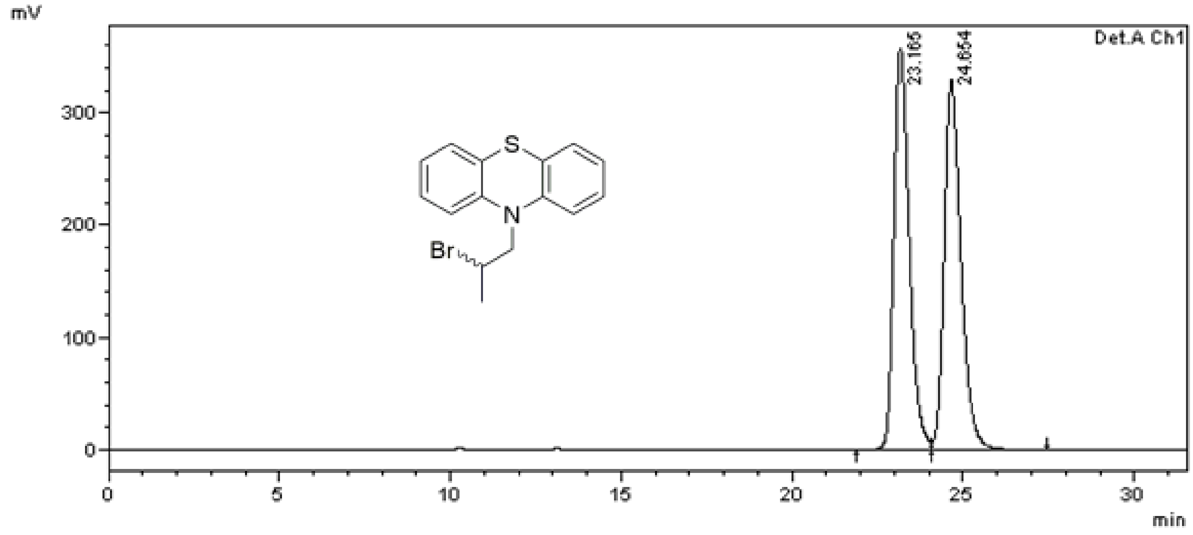

or

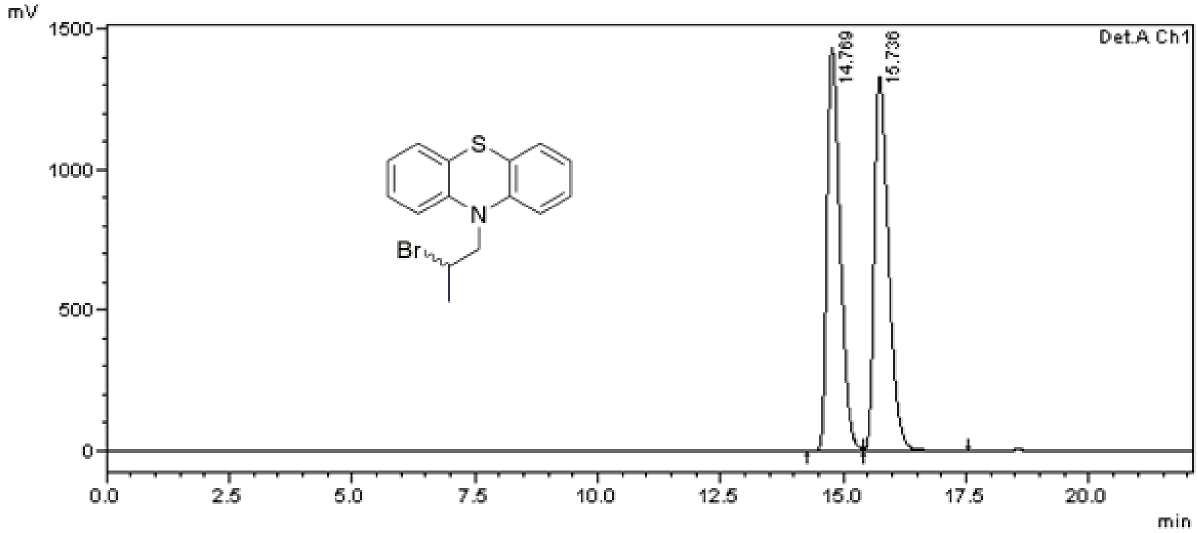

(*R*)-(+)-8 in >99% ee

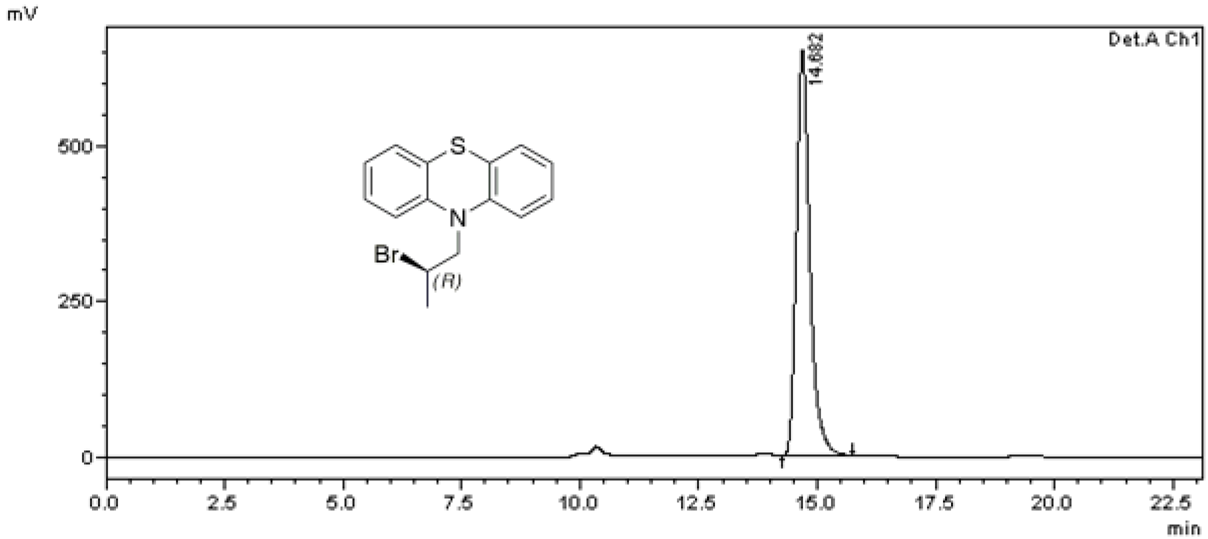

(*S*)-(-)-8 in 96% ee

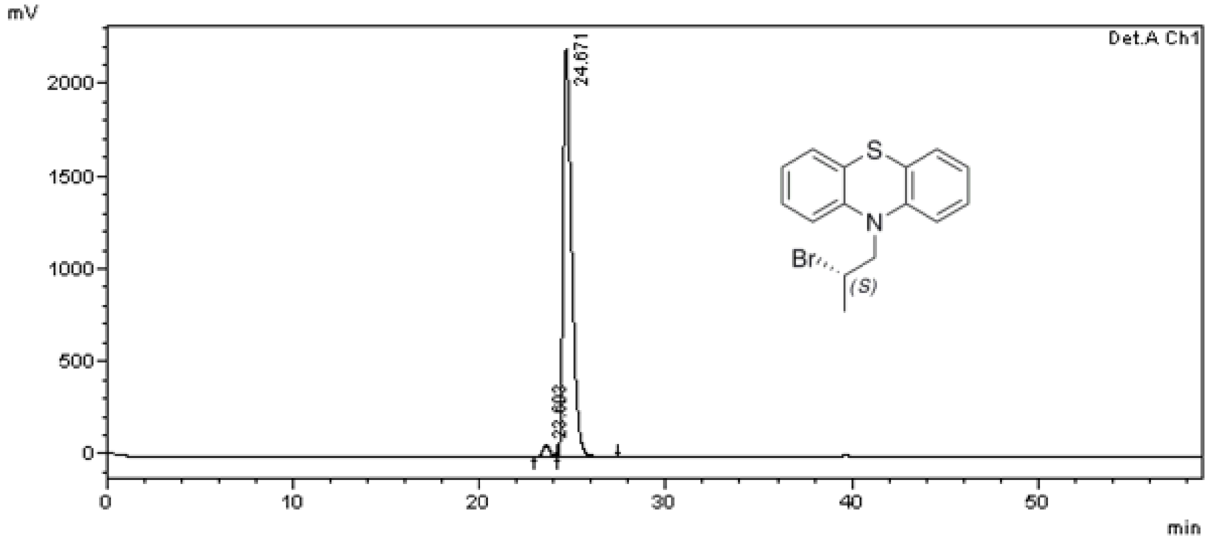

mV
Det.A Ch1
24.671
23.603
Br
(S)
S
N
min

or

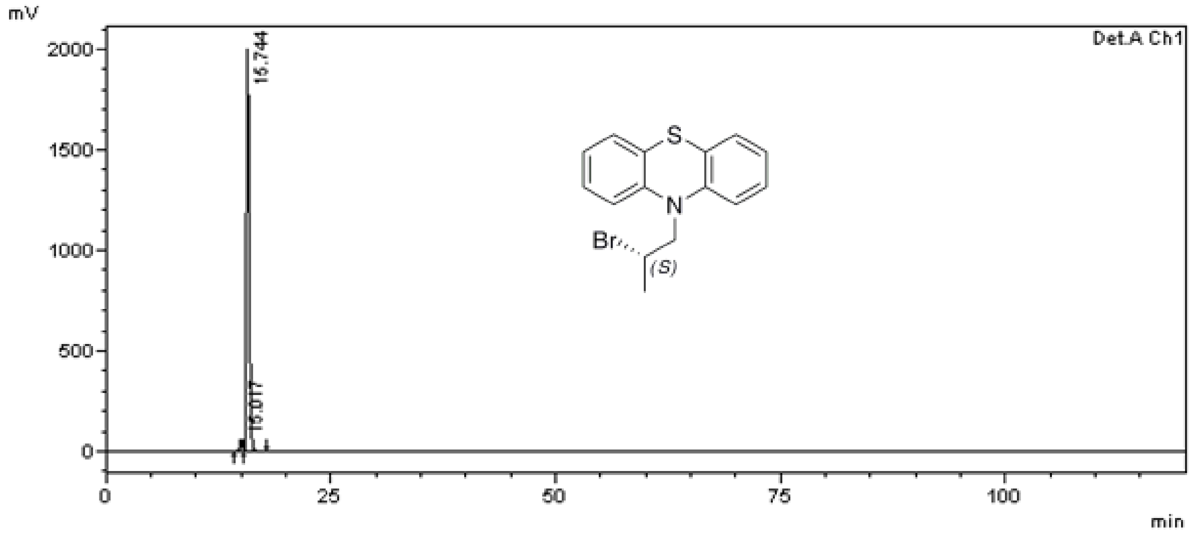

mV
Det.A Ch1
15.744
15.017
Br
(S)
S
N
min

Analytical data for promethazine (±)-9:

HPLC analytical separation for both enantiomers of (±)-9

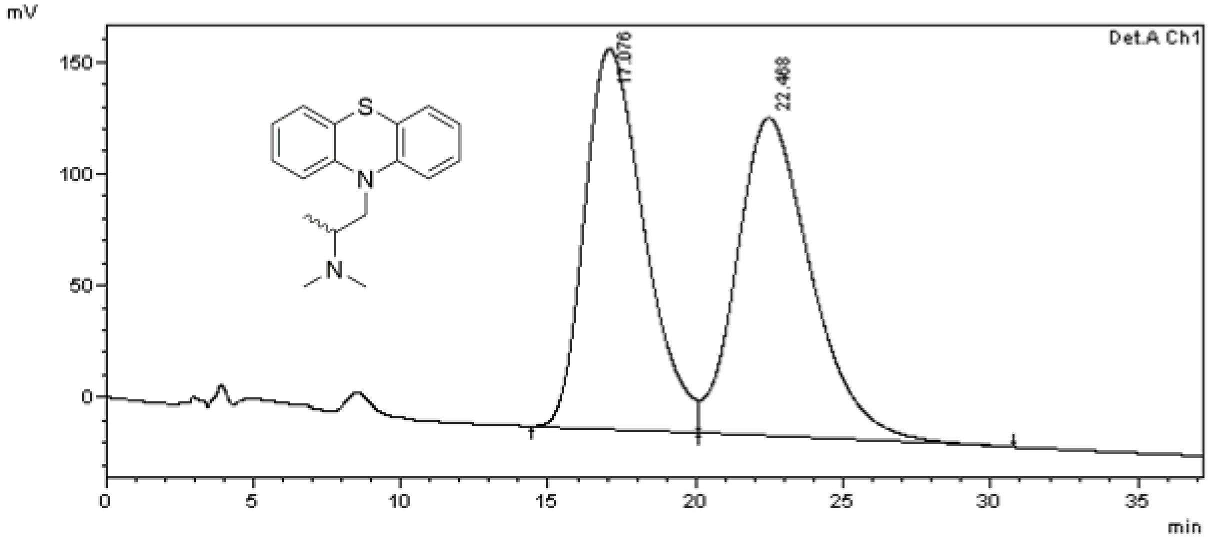

(*S*)-(-)-9 in 84% ee [product of Method A ($PhCH_3$) obtained from (*R*)-(+)-8 (>99% ee)]

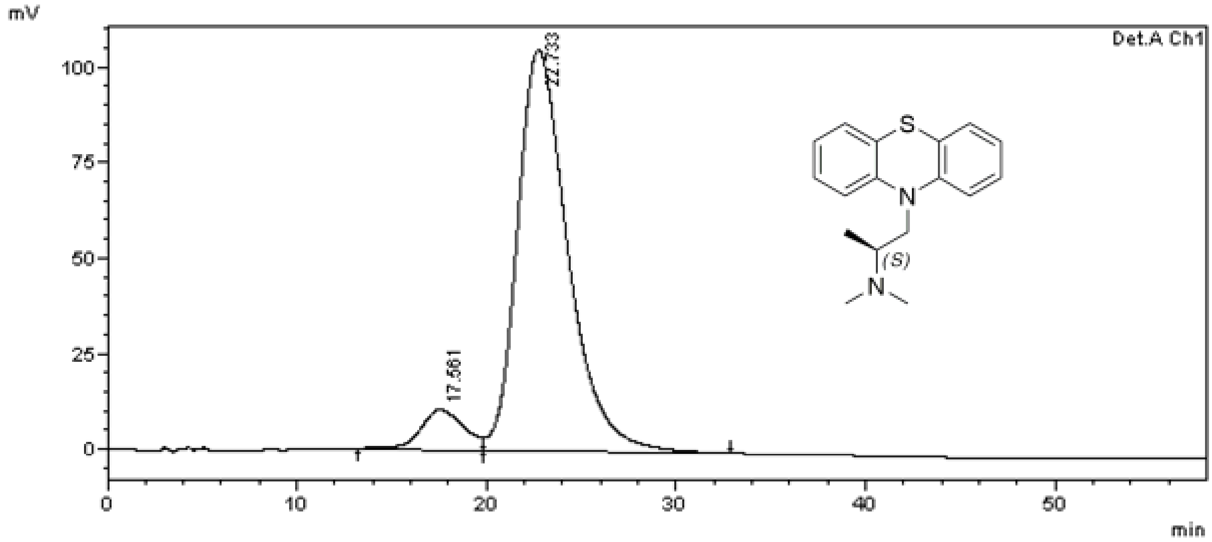

(*R*)-(+)-9 in 92% ee [product of Method A ($PhCH_3$) obtained from (*S*)-(-)-8 (96% ee)]

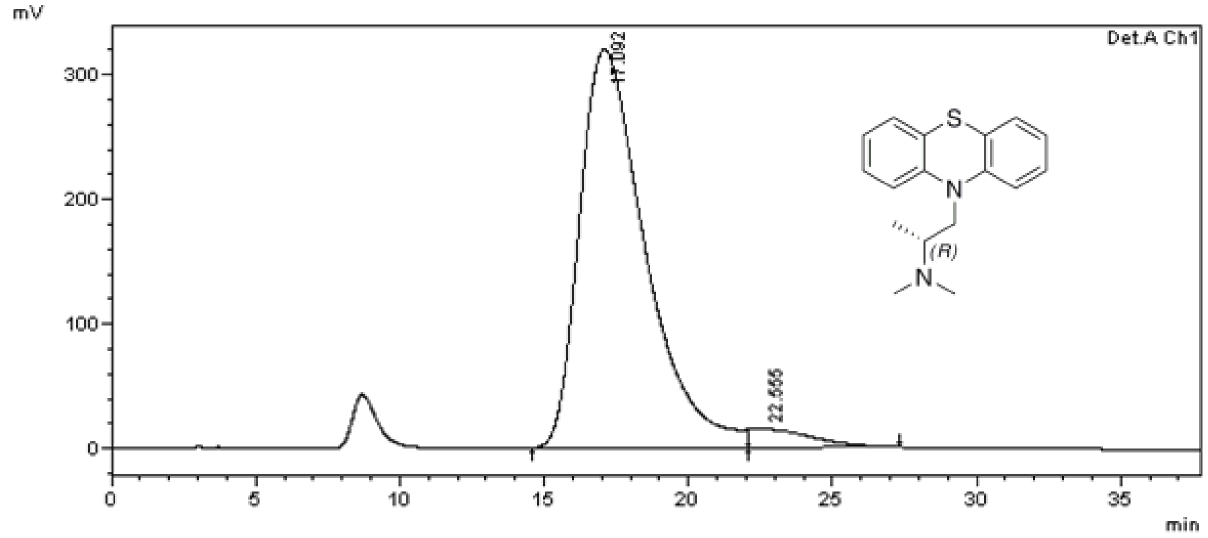

HPLC analytical separation for both enantiomers of (±)-9

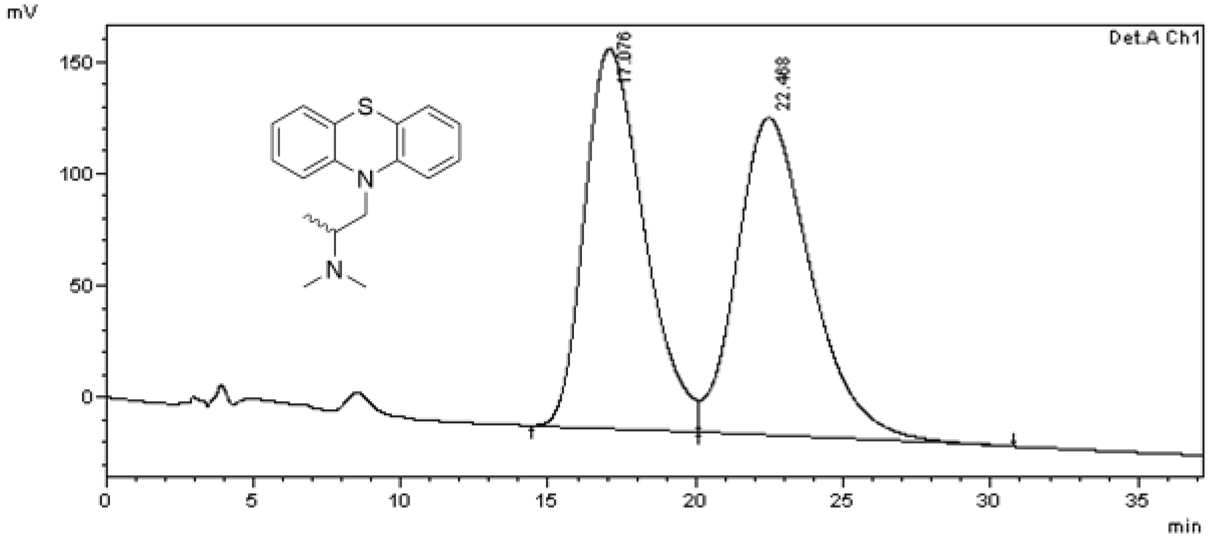

(*R*)-(+)-9 in 97% ee [product of Method B (MeOH) obtained from (*R*)-(+)-8 (>99% ee)]

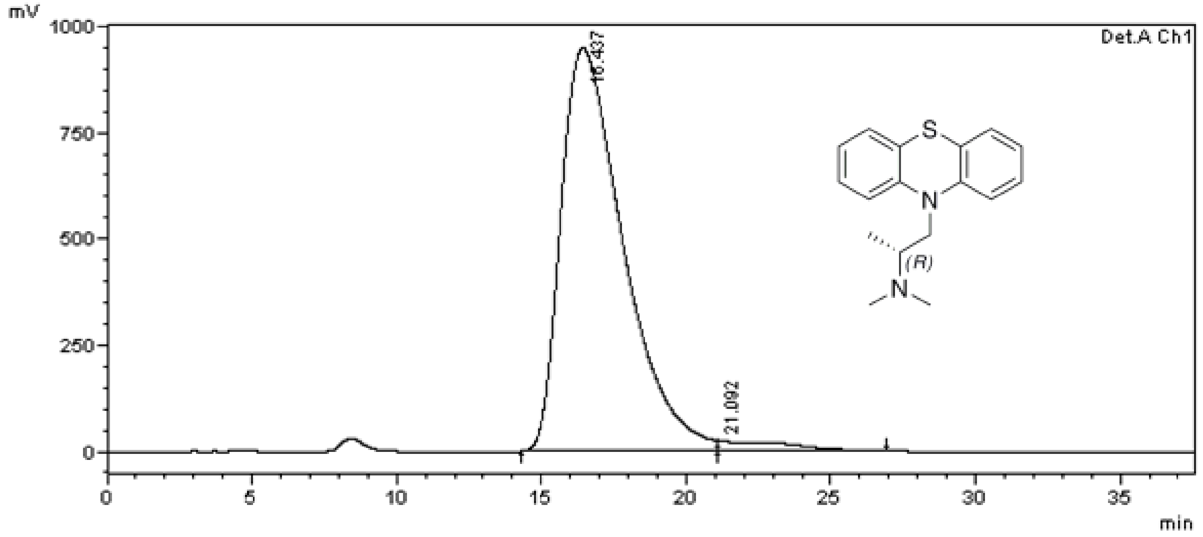

(*S*)-(-)-9 in 94% ee [product of Method B (MeOH) obtained from (*S*)-(-)-8 (96% ee)]

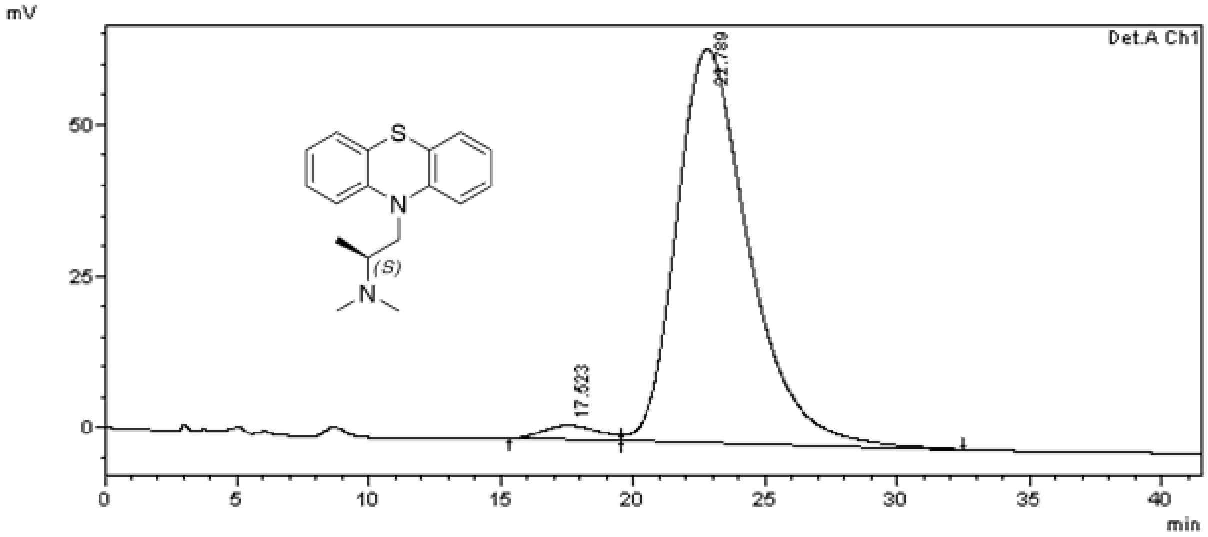

<u>**Analytical data for ethopropazine (±)-10**</u>**:**

HPLC analytical separation for both enantiomers of (±)-10

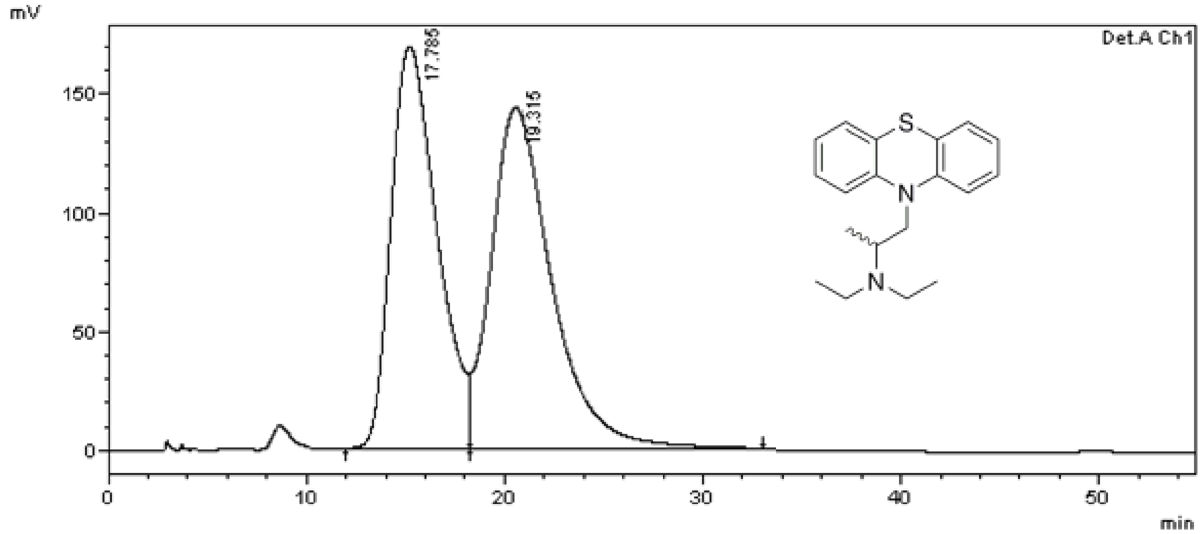

(*S*)-(-)-10 in 90% ee [product of Method A ($PhCH_3$) obtained from (*R*)-(+)-8 (>99% ee)]

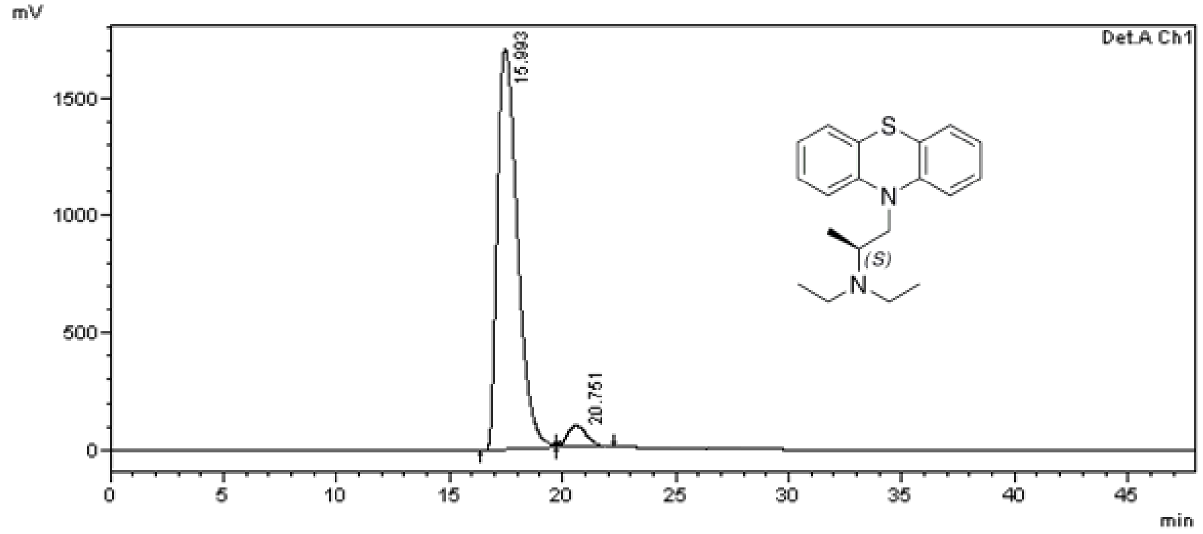

(*R*)-(+)-10 in 93% ee [product of Method A ($PhCH_3$) obtained from (*S*)-(-)-8 (96% ee)]

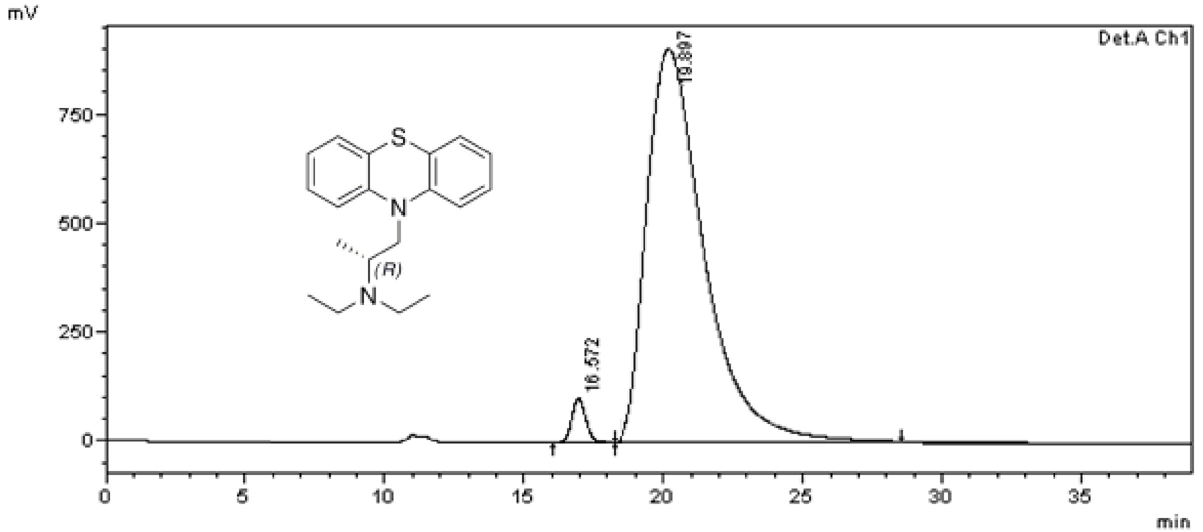

HPLC analytical separation for both enantiomers of (±)-10

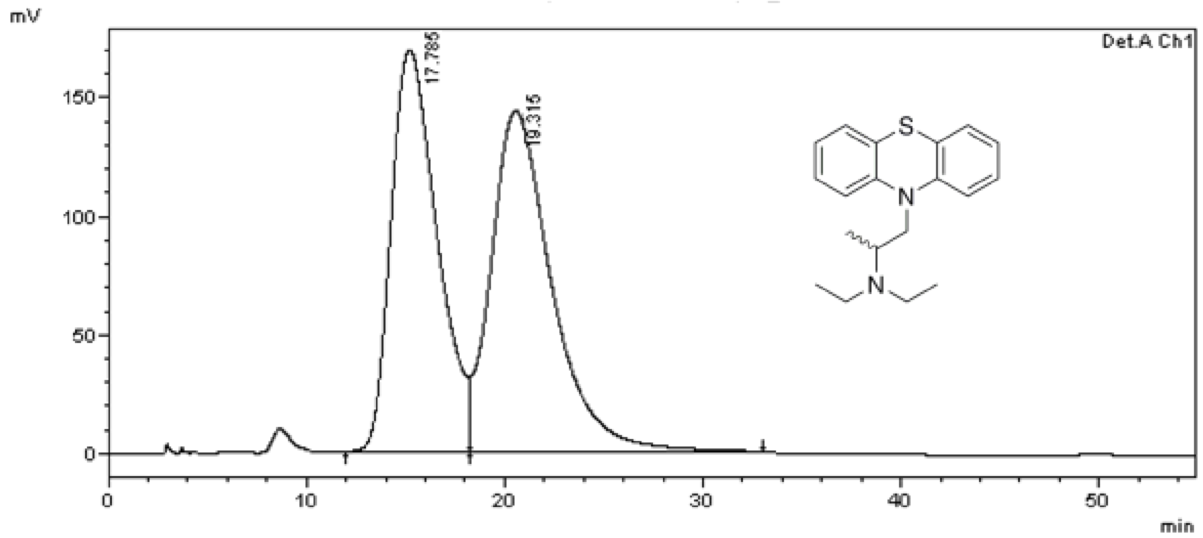

(*R*)-(+)-10 in 98% ee [product of Method B (MeOH) obtained from (*R*)-(+)-8 (>99% ee)]

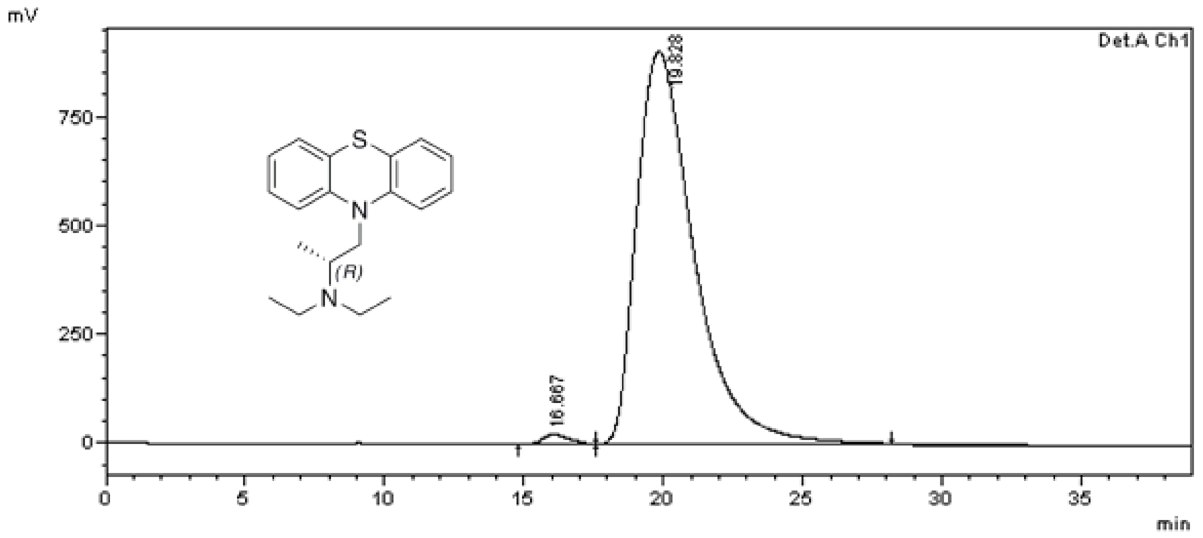

(*S*)-(-)-10 in 96% ee [product of Method B (MeOH) obtained from (*S*)-(-)-8 (96% ee)]

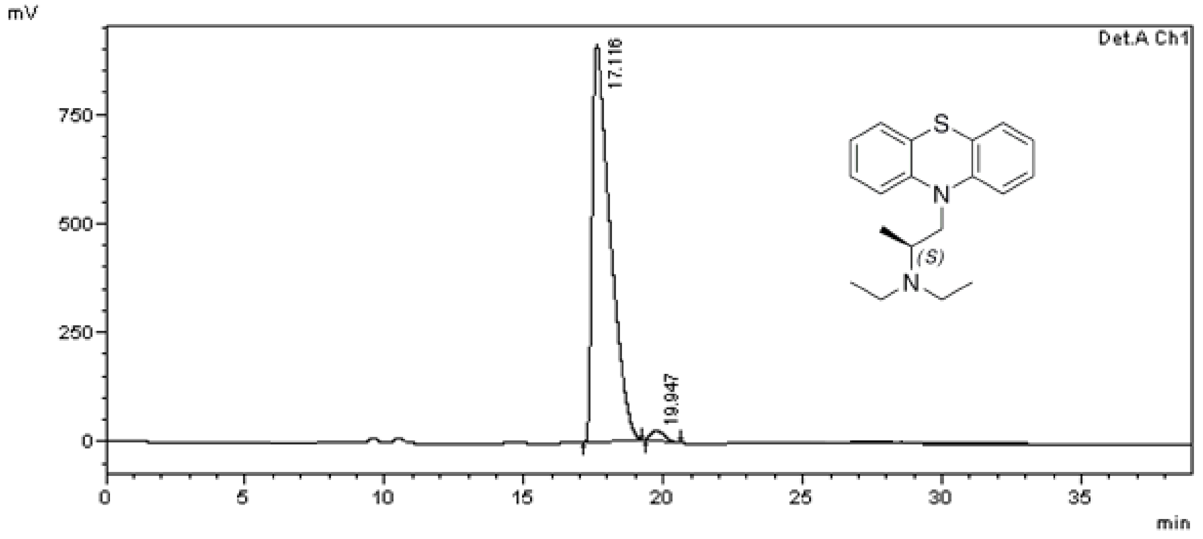

1-(10*H*-Phenothiazin-10-yl)propan-2-ol (±)-3:

Dissolved in $CDCl_3$ and recorded on Varian Mercury 400BB spectrometer 400 MHz

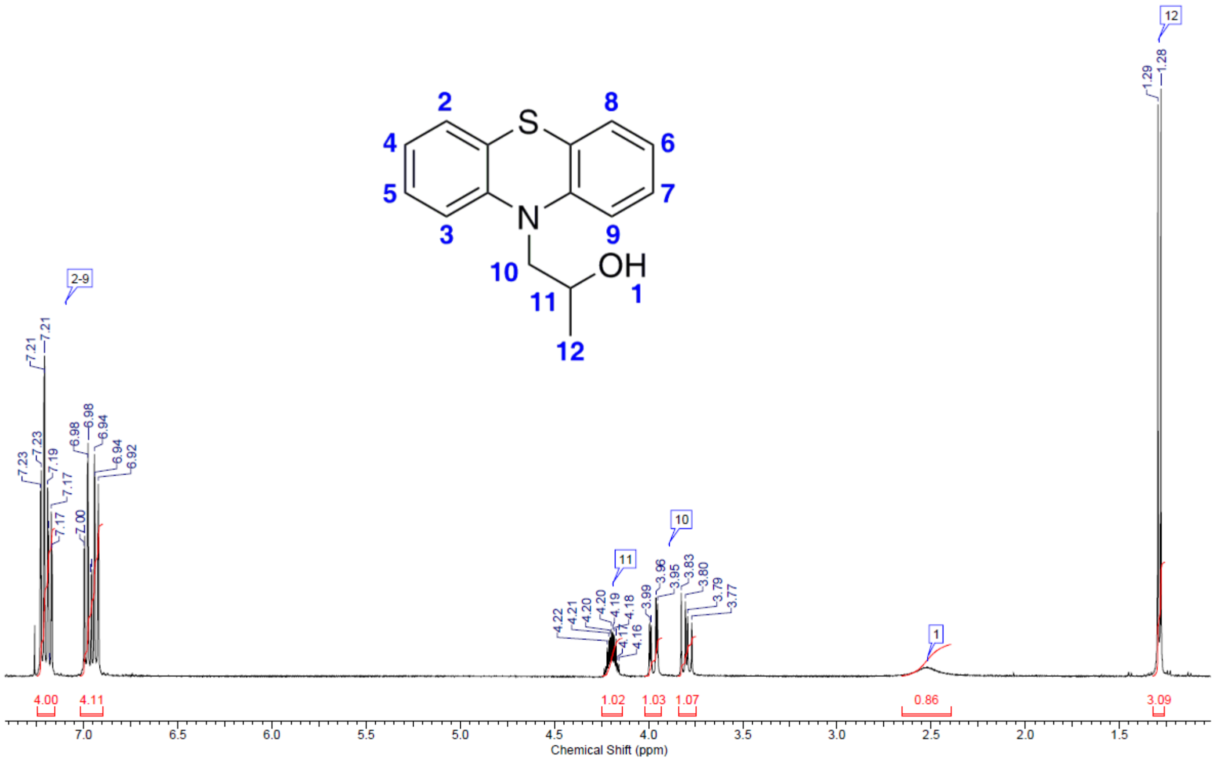

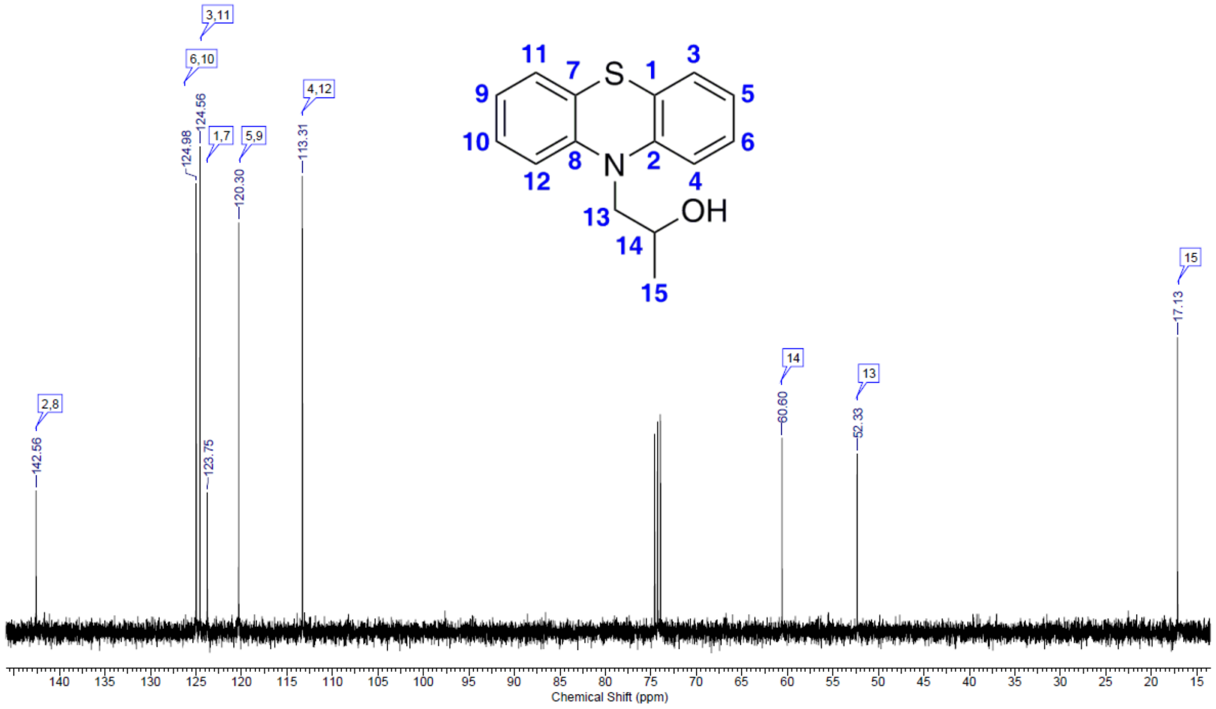

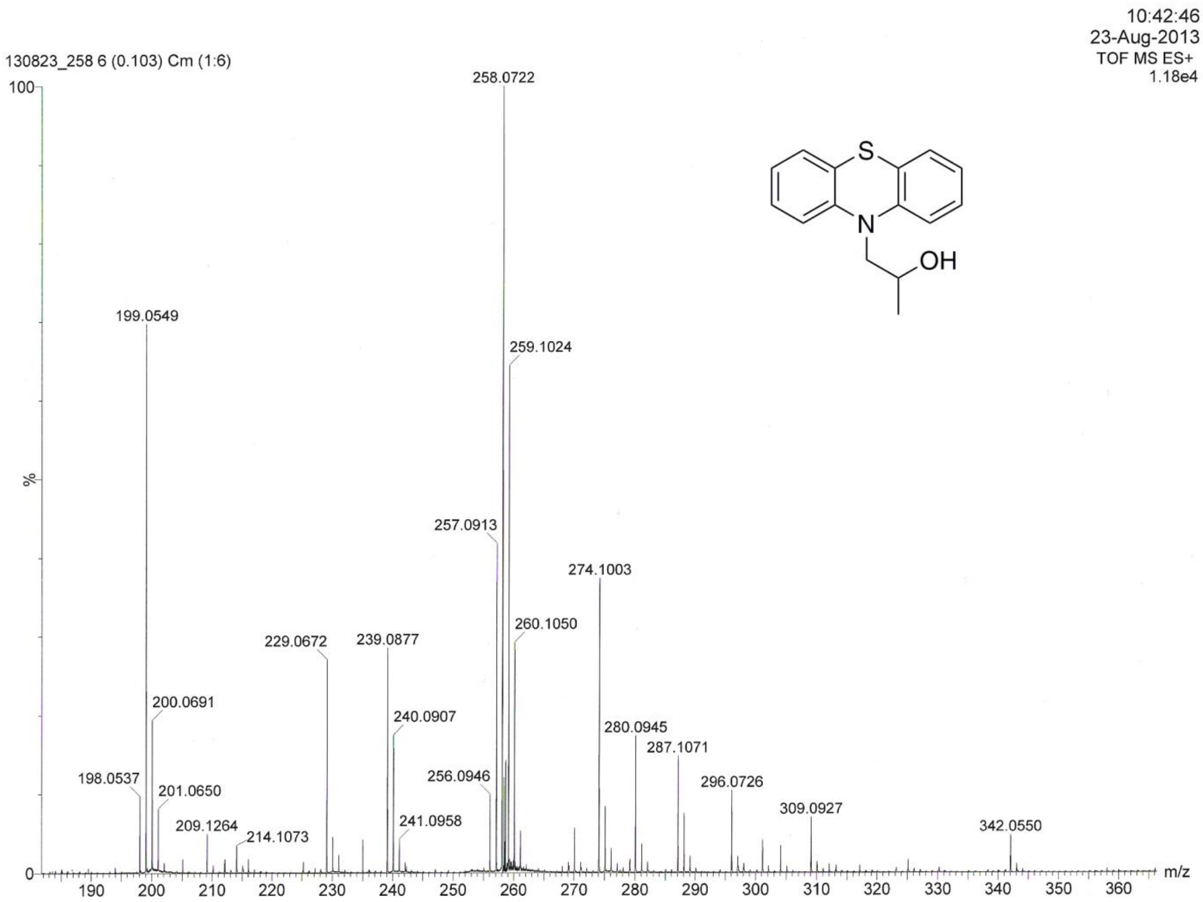
10:42:46
23-Aug-2013
TOF MS ES+
1.18e4
130823_258 6 (0.103) Cm (1:6)
258.0722
199.0549
259.1024
257.0913
274.1003
260.1050
229.0672
239.0877
200.0691
240.0907
280.0945
287.1071
198.0537
201.0650
256.0946
296.0726
309.0927
209.1264
214.1073
241.0958
342.0550
S
N
OH
%
m/z

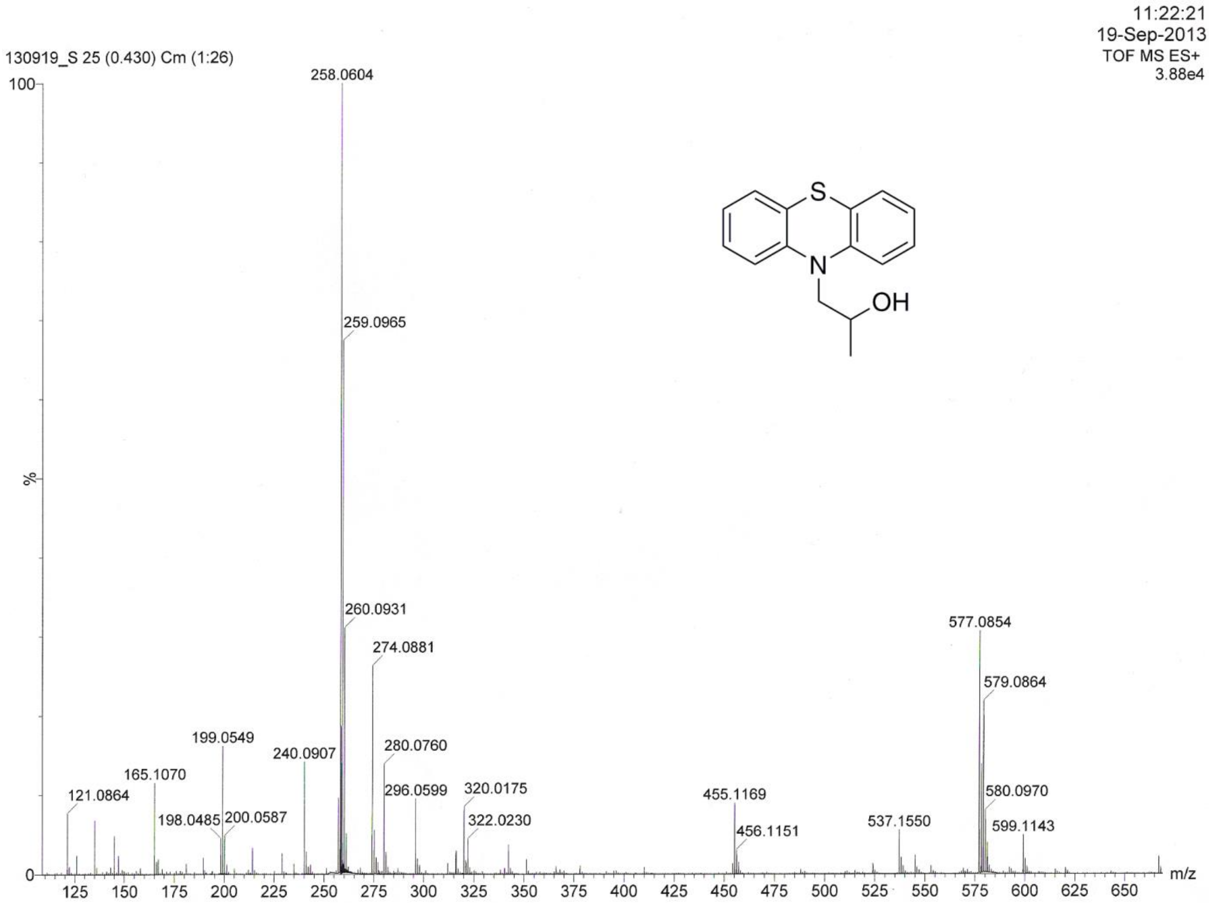
11:22:21
19-Sep-2013
TOF MS ES+
3.88e4
130919_S 25 (0.430) Cm (1:26)
258.0604
259.0965
260.0931
274.0881
577.0854
579.0864
199.0549
280.0760
240.0907
165.1070
320.0175
296.0599
121.0864
455.1169
580.0970
198.0485
200.0587
322.0230
537.1550
599.1143
456.1151
S
N
OH
%
m/z

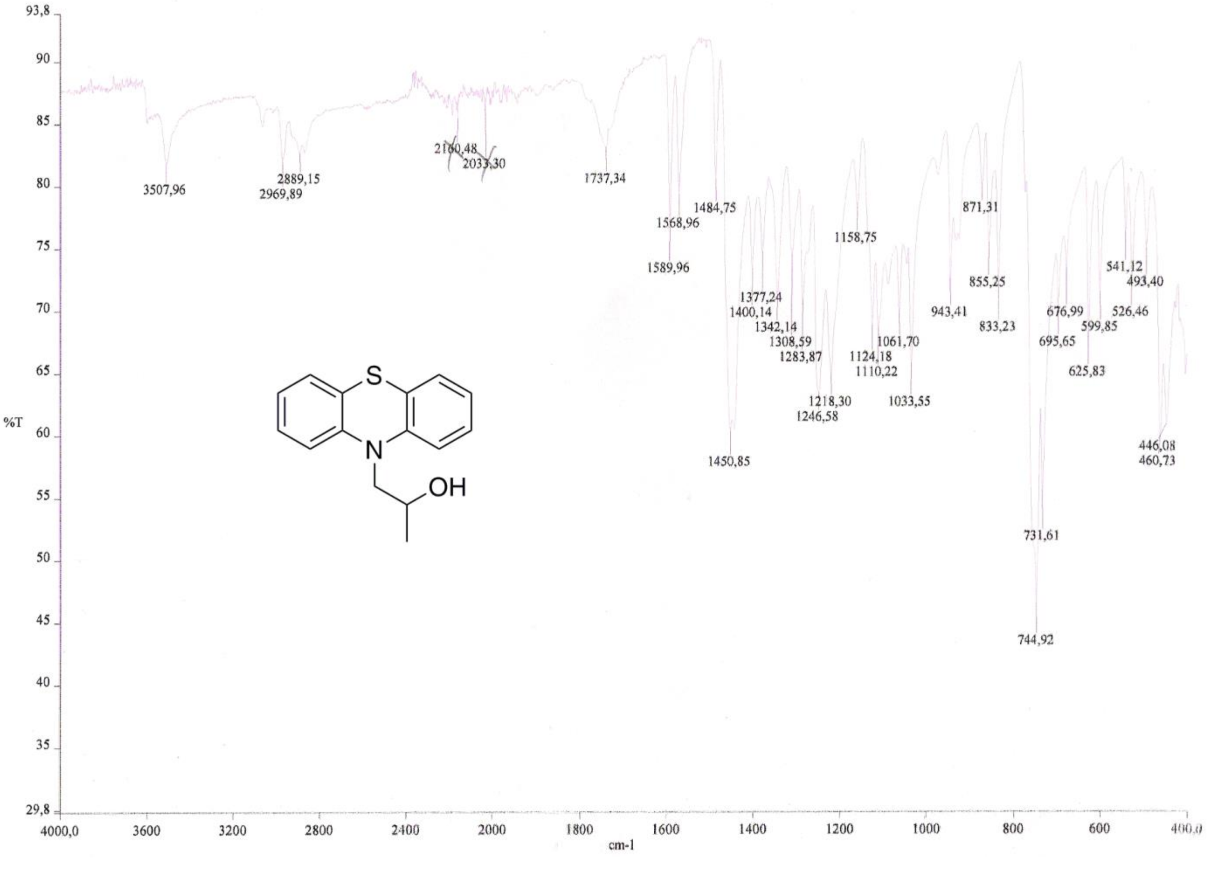
3507,96
2969,89
2889,15
2160,48
2033,30
1737,34
1589,96
1568,96
1484,75
1450,85
1400,14
1377,24
1342,14
1308,59
1283,87
1246,58
1218,30
1158,75
1124,18
1110,22
1061,70
1033,55
943,41
871,31
855,25
833,23
744,92
731,61
695,65
676,99
625,83
599,85
541,12
526,46
493,40
460,73
446,08
%T
cm-1
S
N
OH

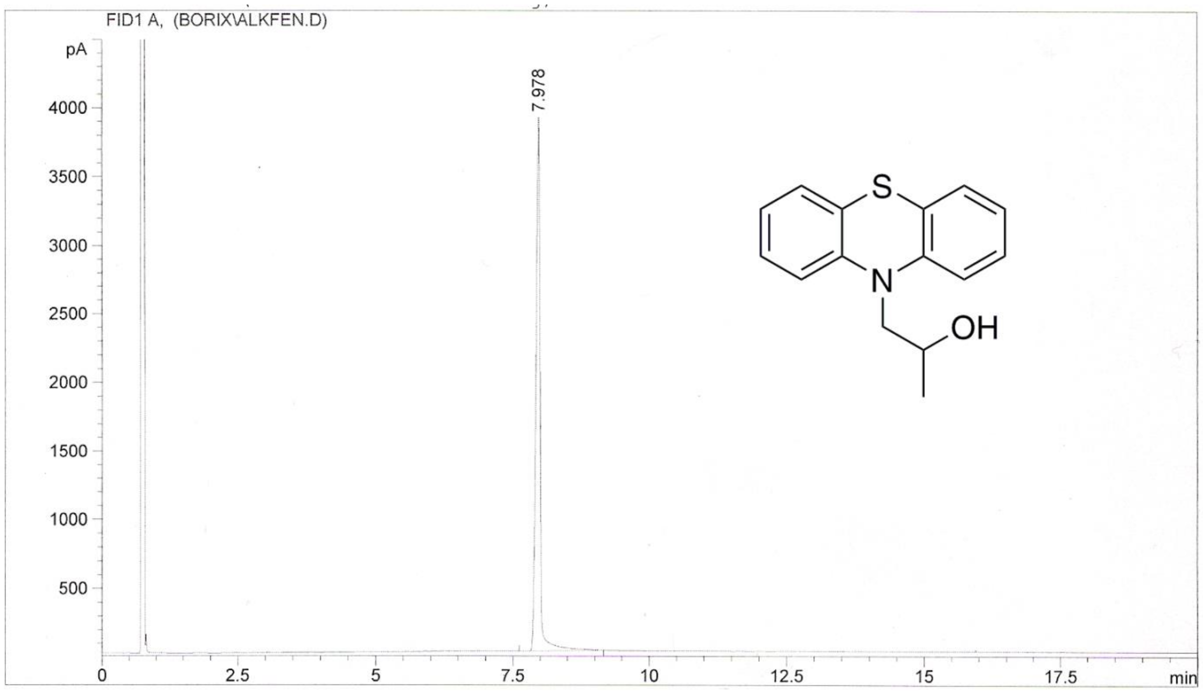
FID1 A, (BORIX\ALKFEN.D)
pA
7.978
min
S
N
OH

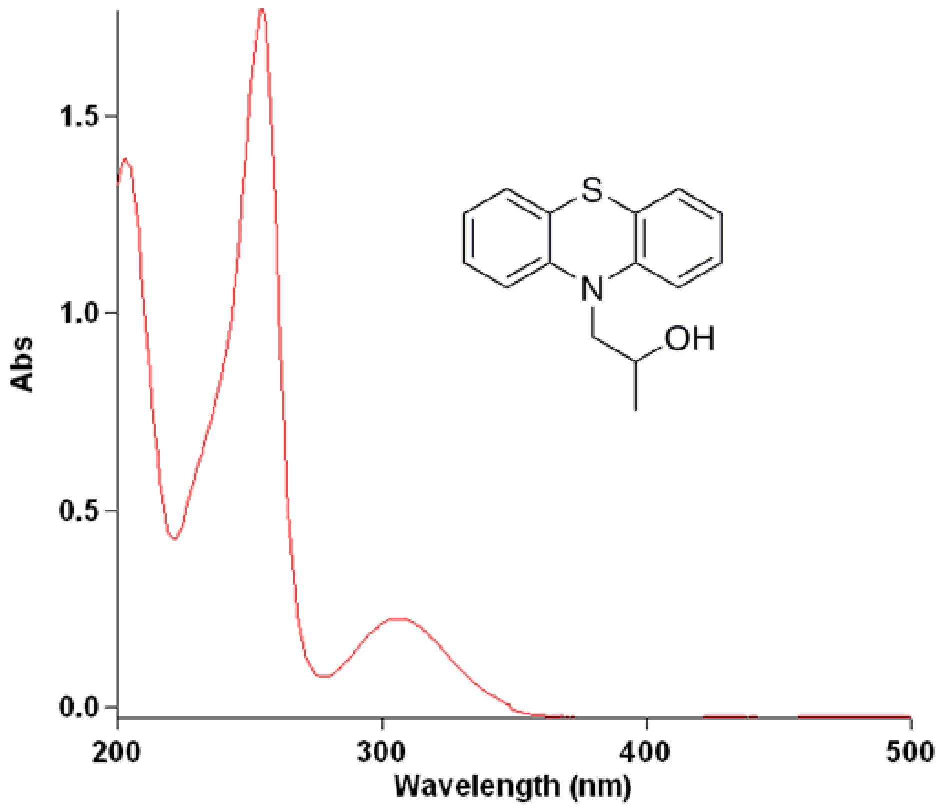

Wavelength (nm)	Abs
305.00	0.226
255.00	1.770
203.00	1.395

1-(10*H*-Phenothiazin-10-yl)propan-2-yl acetate (±)-4a

Dissolved in CD_3OD and recorded on Varian Mercury 400BB spectrometer 400 MHz

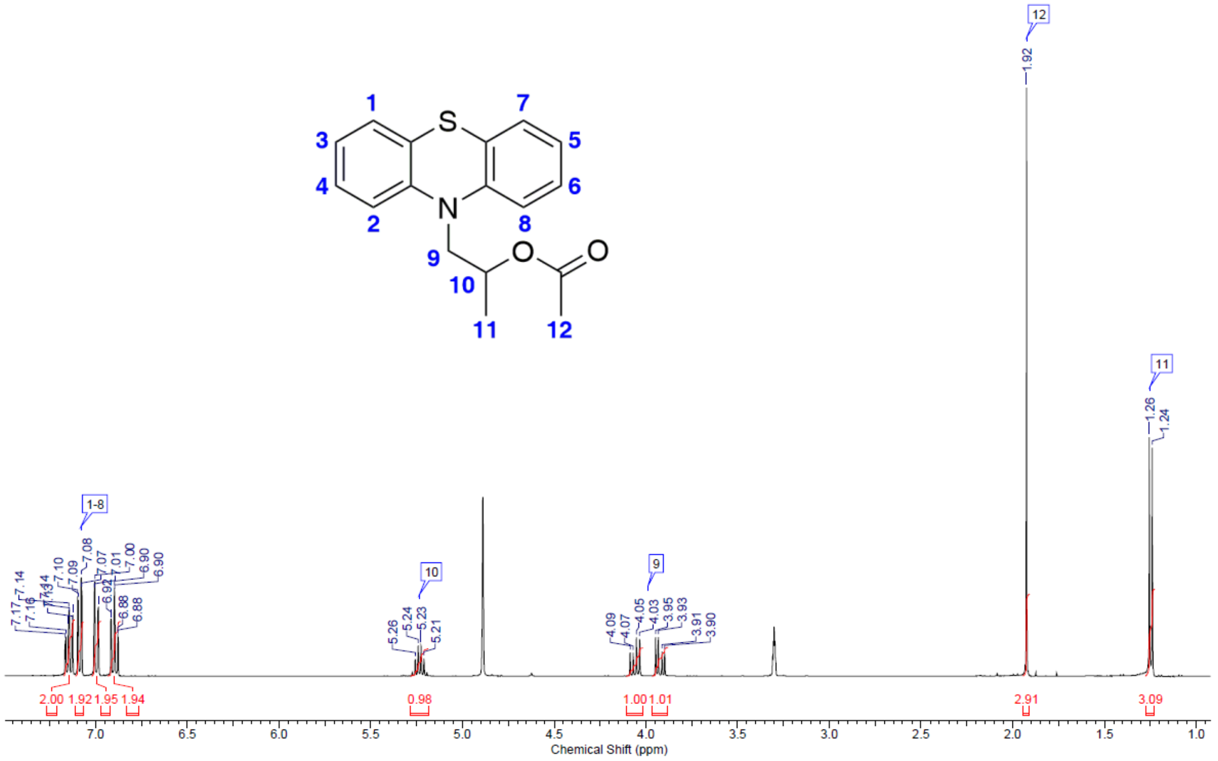

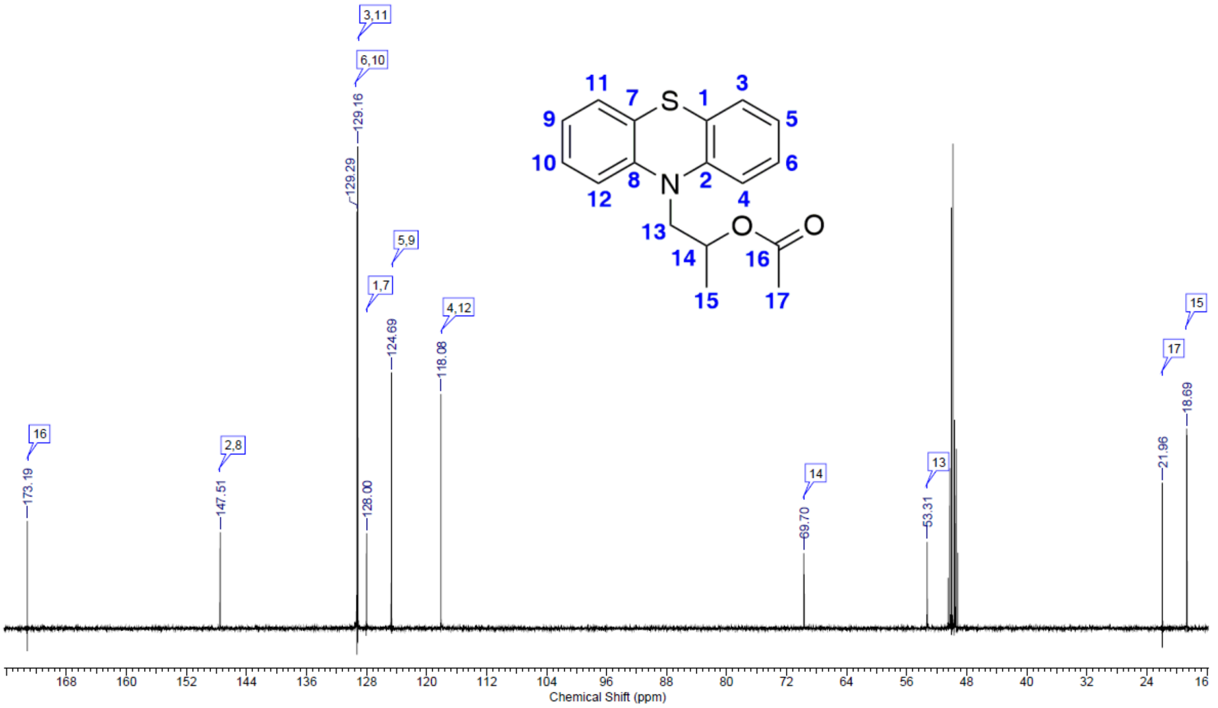

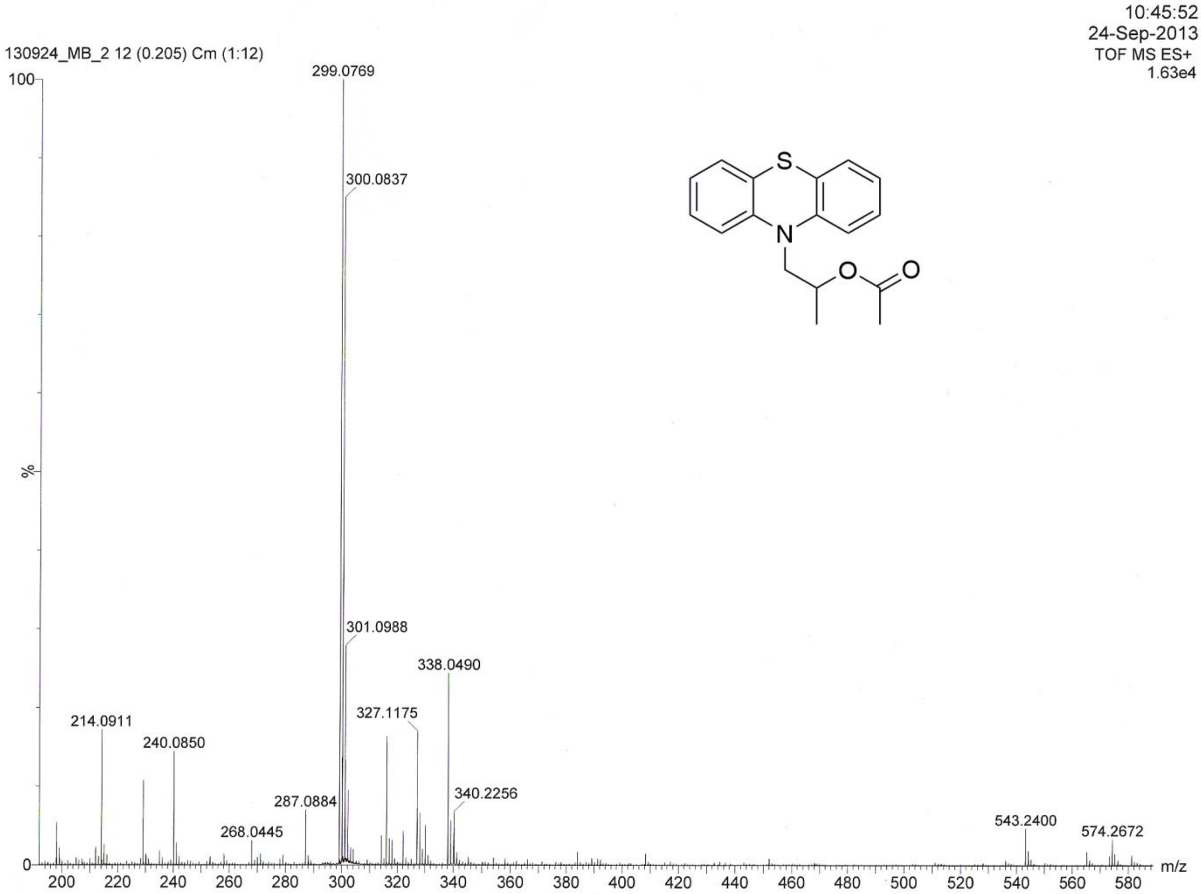

10:45:52
24-Sep-2013
TOF MS ES+
1.63e4
130924_MB_2 12 (0.205) Cm (1:12)
299.0769
300.0837
301.0988
338.0490
327.1175
214.0911
240.0850
340.2256
287.0884
268.0445
543.2400
574.2672
%
m/z

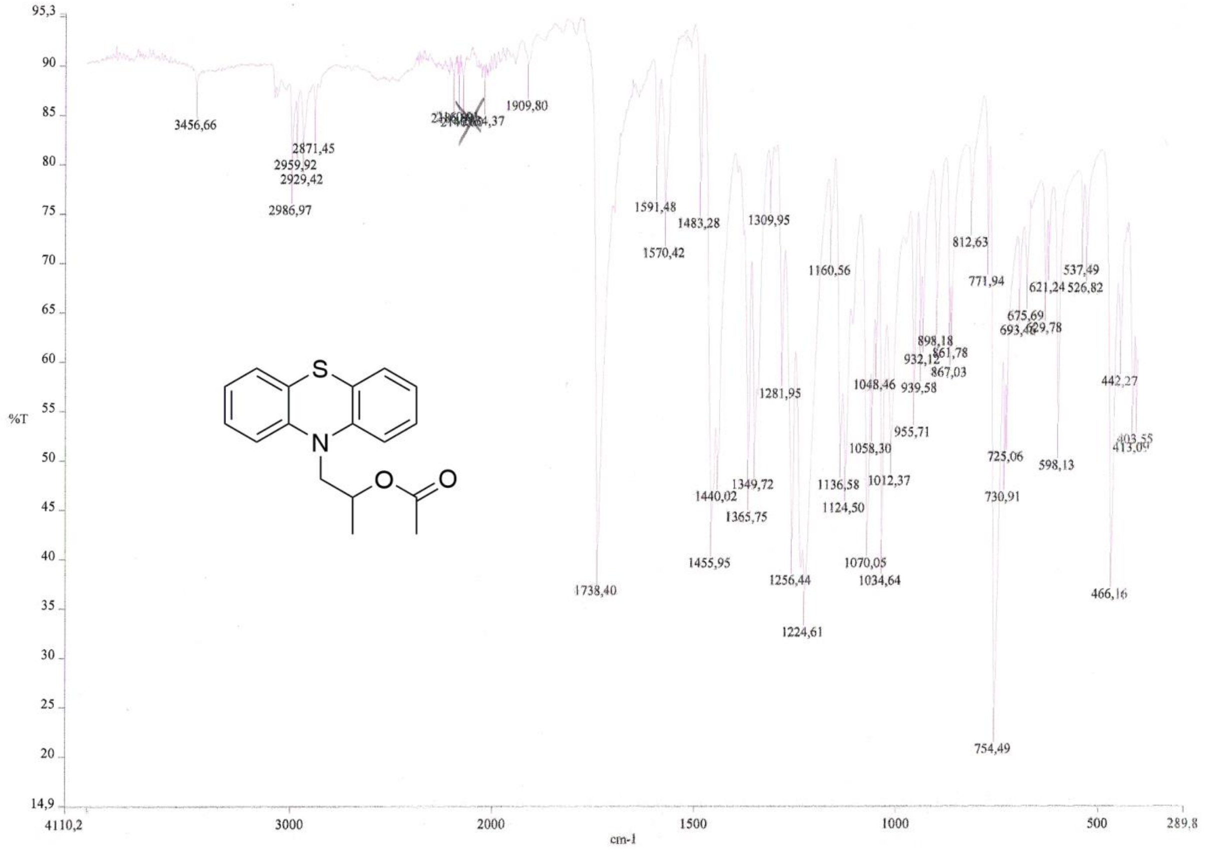

%T
cm-1
3456,66
2871,45
2959,92
2929,42
2986,97
1909,80
1738,40
1591,48
1570,42
1483,28
1455,95
1440,02
1365,75
1349,72
1309,95
1281,95
1256,44
1224,61
1160,56
1136,58
1124,50
1070,05
1058,30
1048,46
1034,64
1012,37
955,71
939,58
932,12
898,18
867,03
861,78
812,63
771,94
754,49
730,91
725,06
693,46
675,69
629,78
621,24
598,13
537,49
526,82
466,16
442,27
413,09
403,55

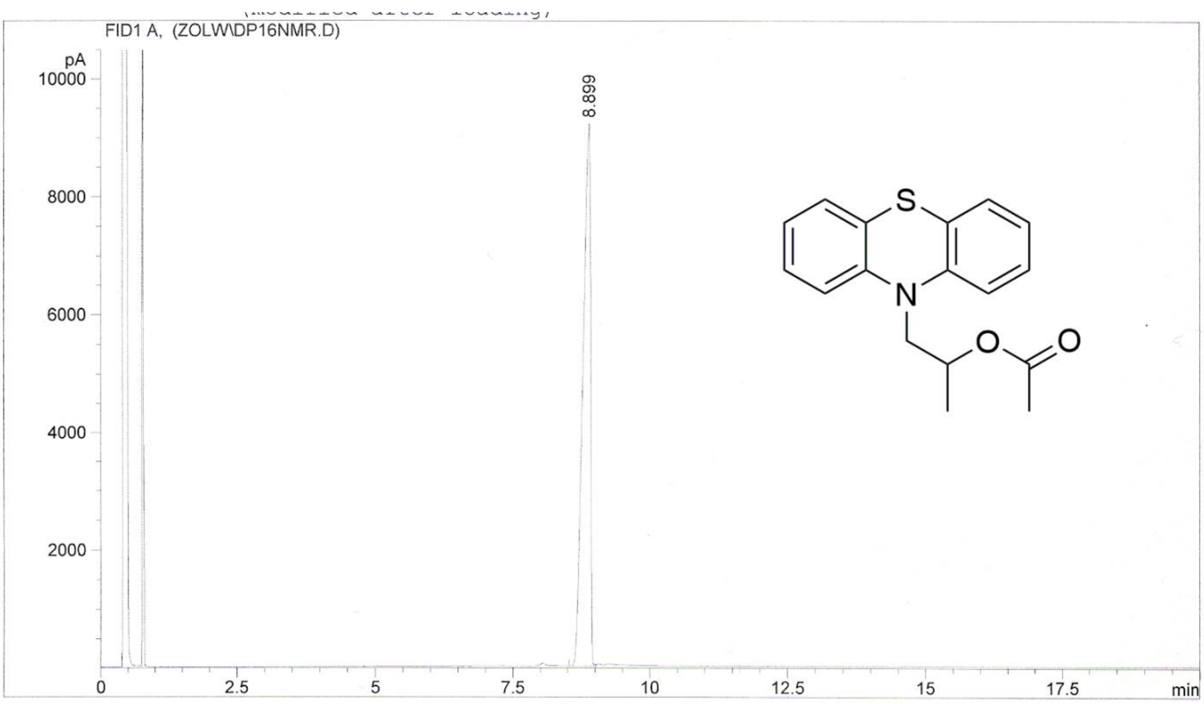
FID1 A, (ZOLW\DP16NMR.D)
pA
10000
8000
6000
4000
2000
8.899
0
2.5
5
7.5
10
12.5
15
17.5
min
S
N
O
O

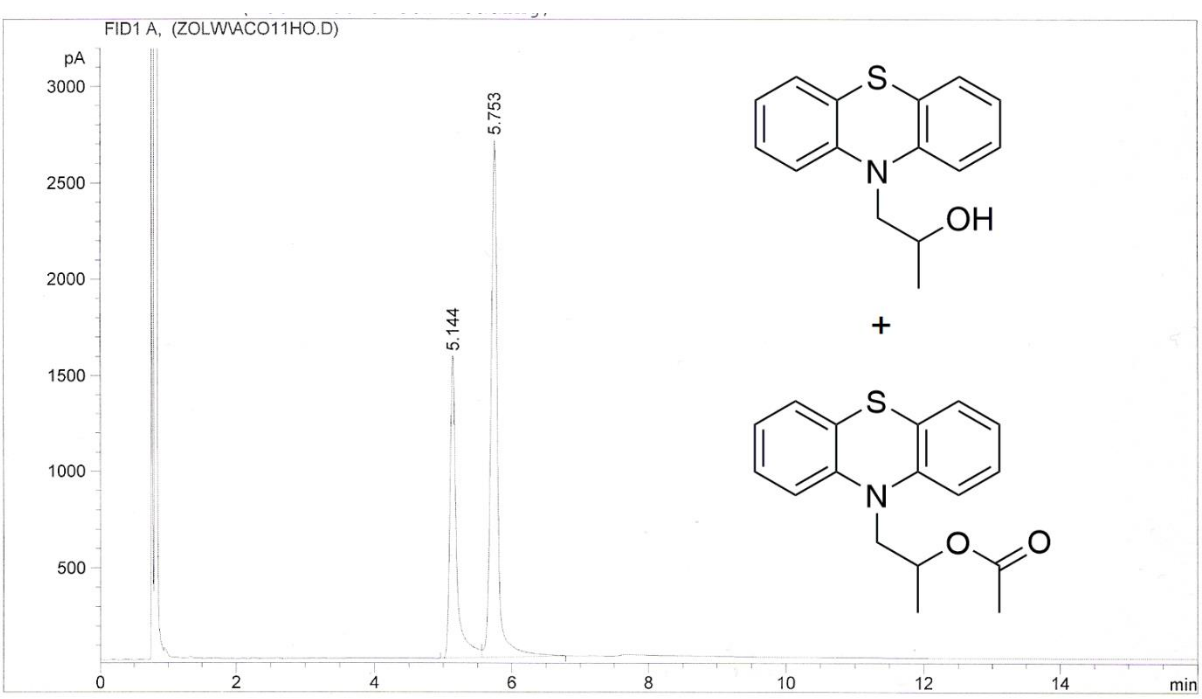
FID1 A, (ZOLW\ACO11HO.D)
pA
3000
2500
2000
1500
1000
500
5.144
5.753
0
2
4
6
8
10
12
14
min
S
N
OH
+
S
N
O
O

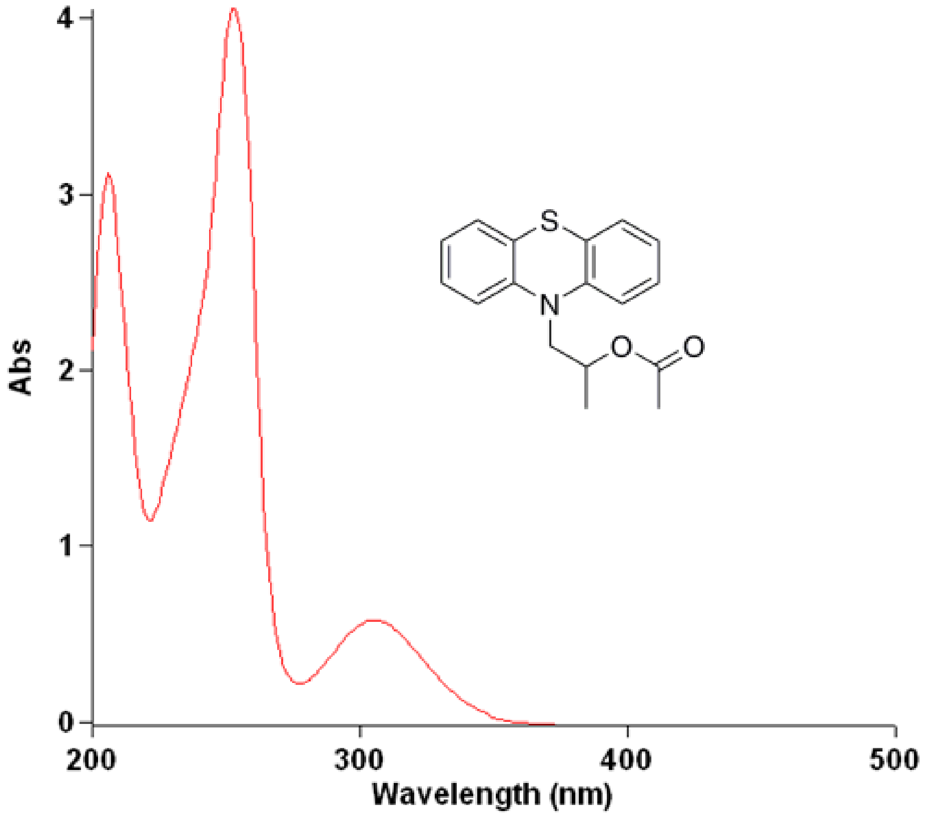

Wavelength (nm)	Abs
305.00	0.577
253.00	4.056
206.00	3.126

1-(10*H*-Phenothiazin-10-yl)propan-2-yl butanoate (±)-4b

Dissolved in CD_3OD and recorded on Varian Mercury 400BB spectrometer 400 MHz

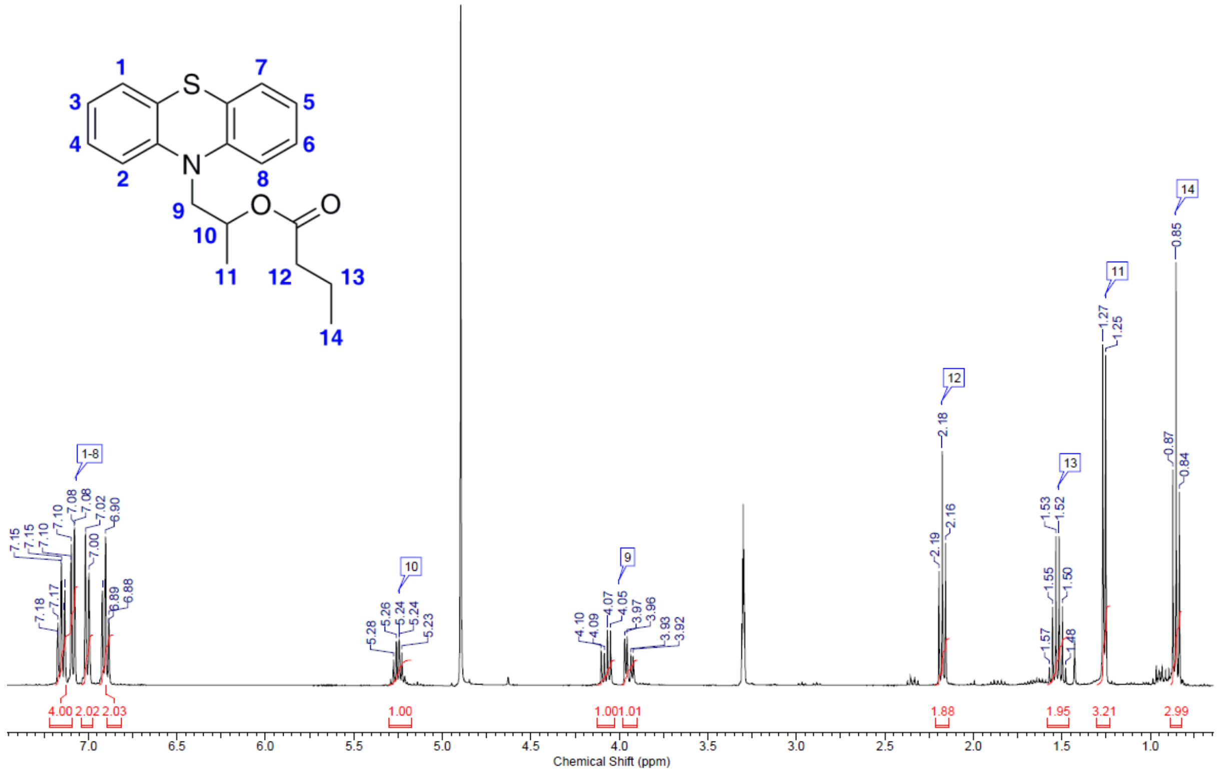

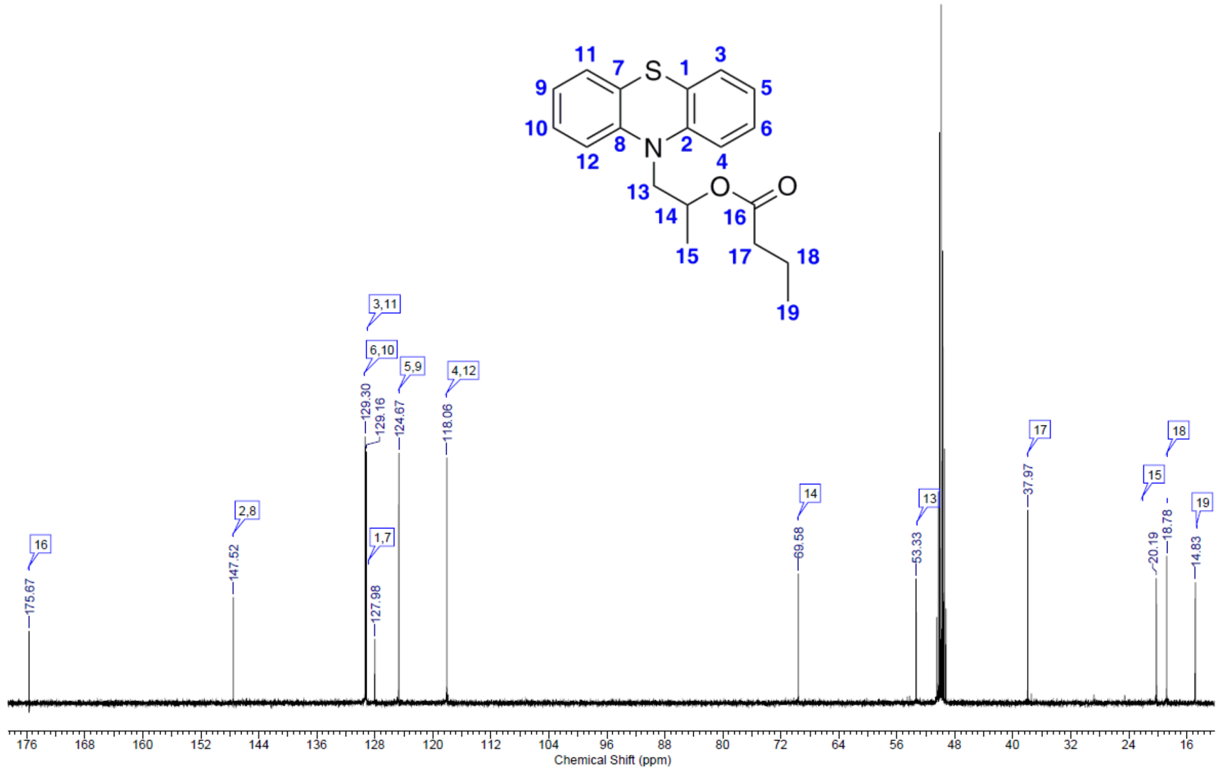

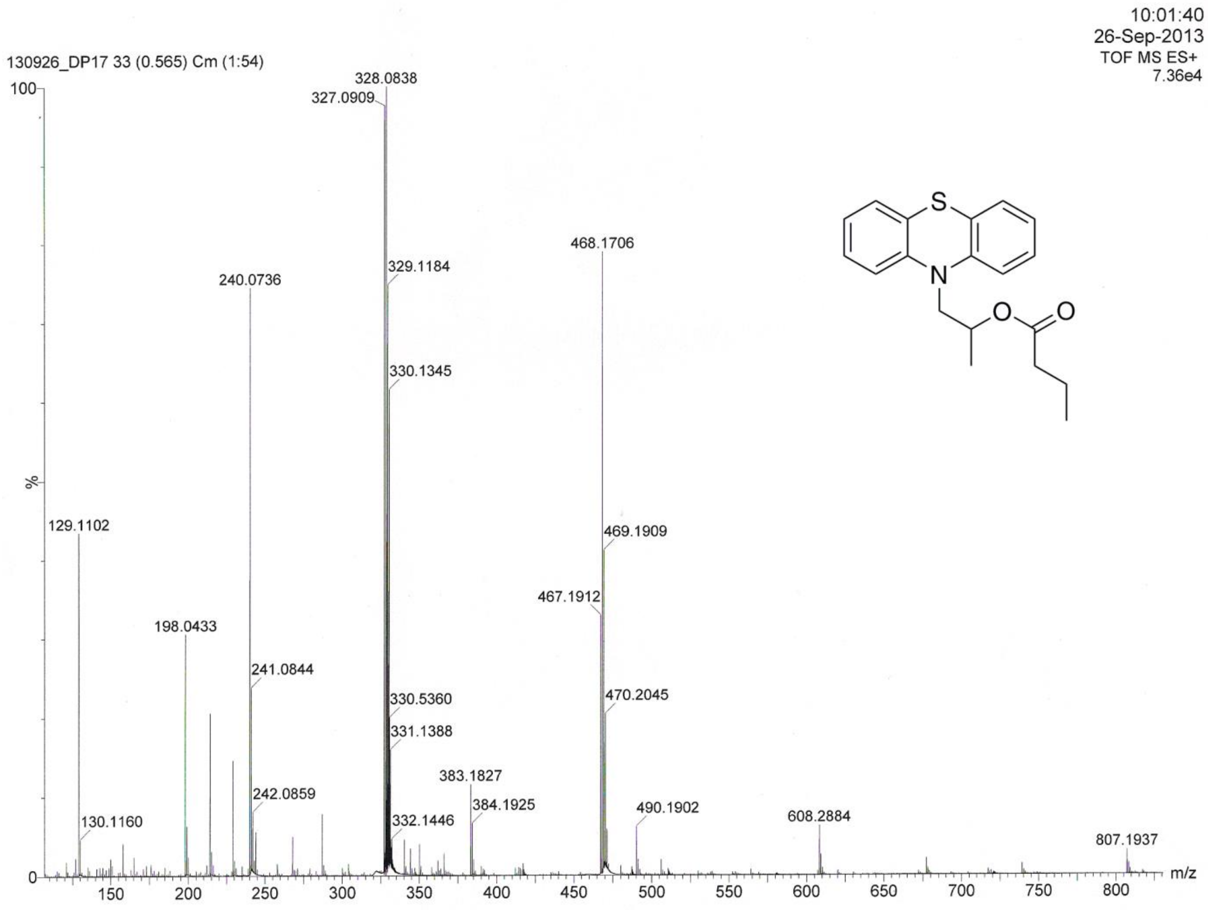
10:01:40
26-Sep-2013
TOF MS ES+
7.36e4
130926_DP17 33 (0.565) Cm (1:54)
328.0838
327.0909
468.1706
329.1184
240.0736
330.1345
129.1102
469.1909
467.1912
198.0433
241.0844
470.2045
330.5360
331.1388
383.1827
242.0859
384.1925
490.1902
608.2884
130.1160
332.1446
807.1937
m/z
%
S
N
O
O

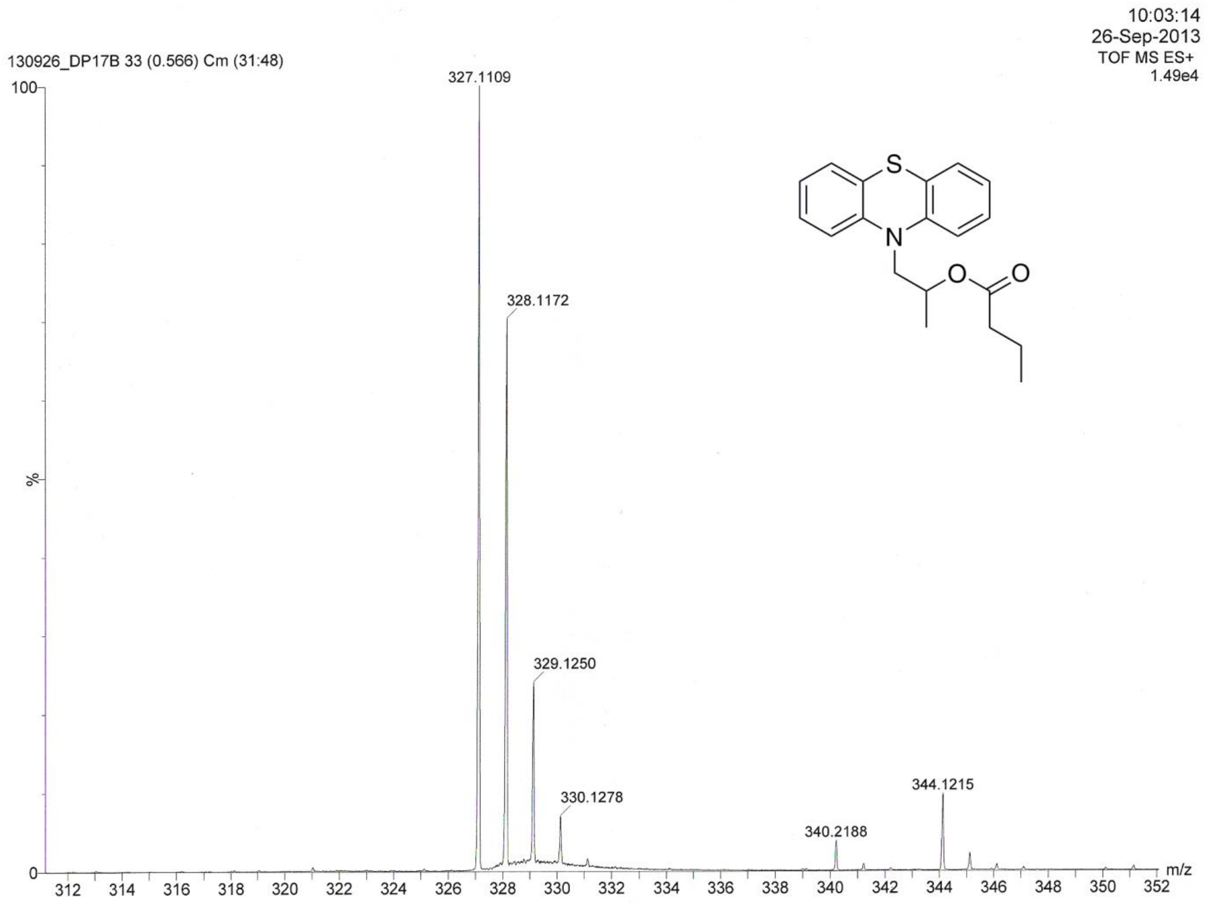
10:03:14
26-Sep-2013
TOF MS ES+
1.49e4
130926_DP17B 33 (0.566) Cm (31:48)
327.1109
328.1172
329.1250
330.1278
344.1215
340.2188
m/z
%
S
N
O
O

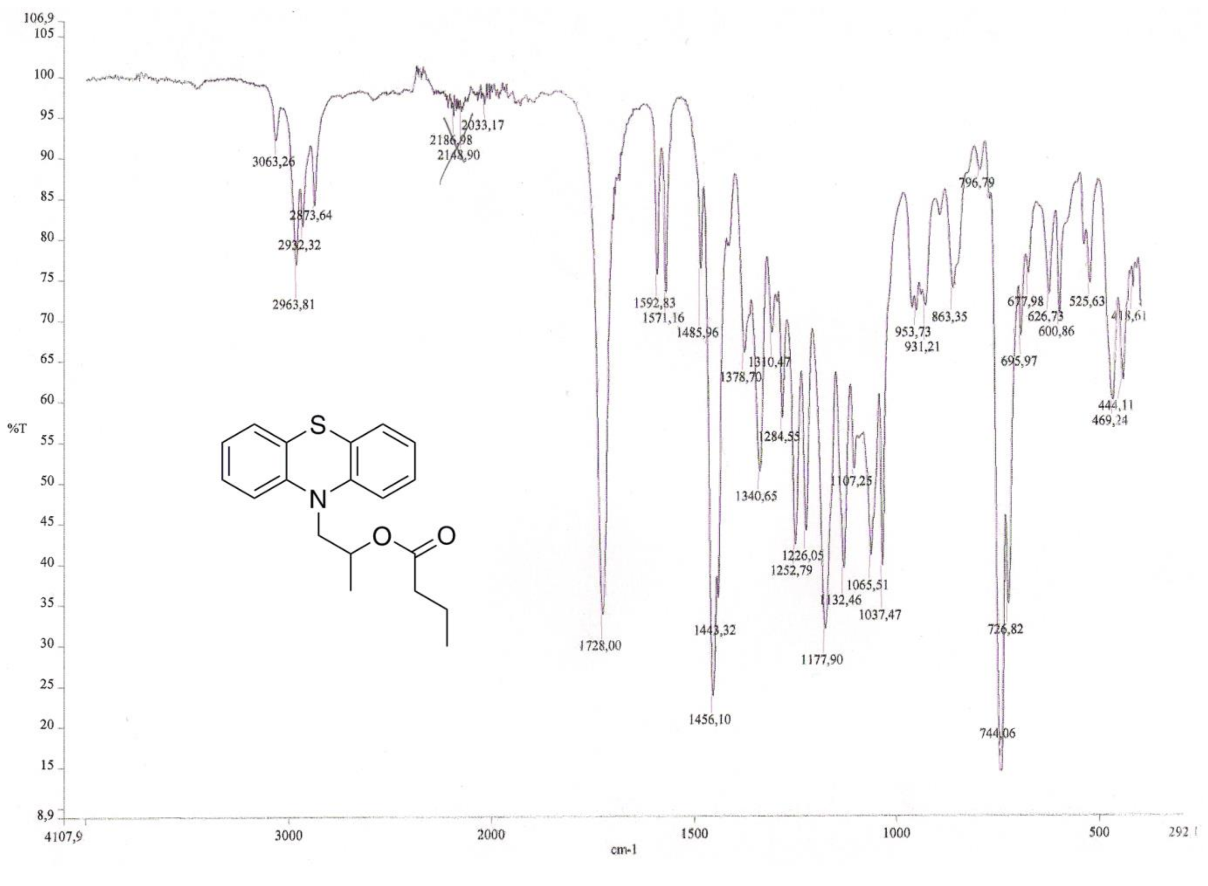

%T
cm-1
3063,26
2963,81
2932,32
2873,64
2186,98
2148,90
2033,17
1728,00
1592,83
1571,16
1485,96
1456,10
1443,32
1378,70
1340,65
1310,47
1284,55
1252,79
1226,05
1177,90
1132,46
1107,25
1065,51
1037,47
953,73
931,21
863,35
796,79
744,06
726,82
695,97
677,98
626,73
600,86
525,63
469,24
444,11
S
N
O
O

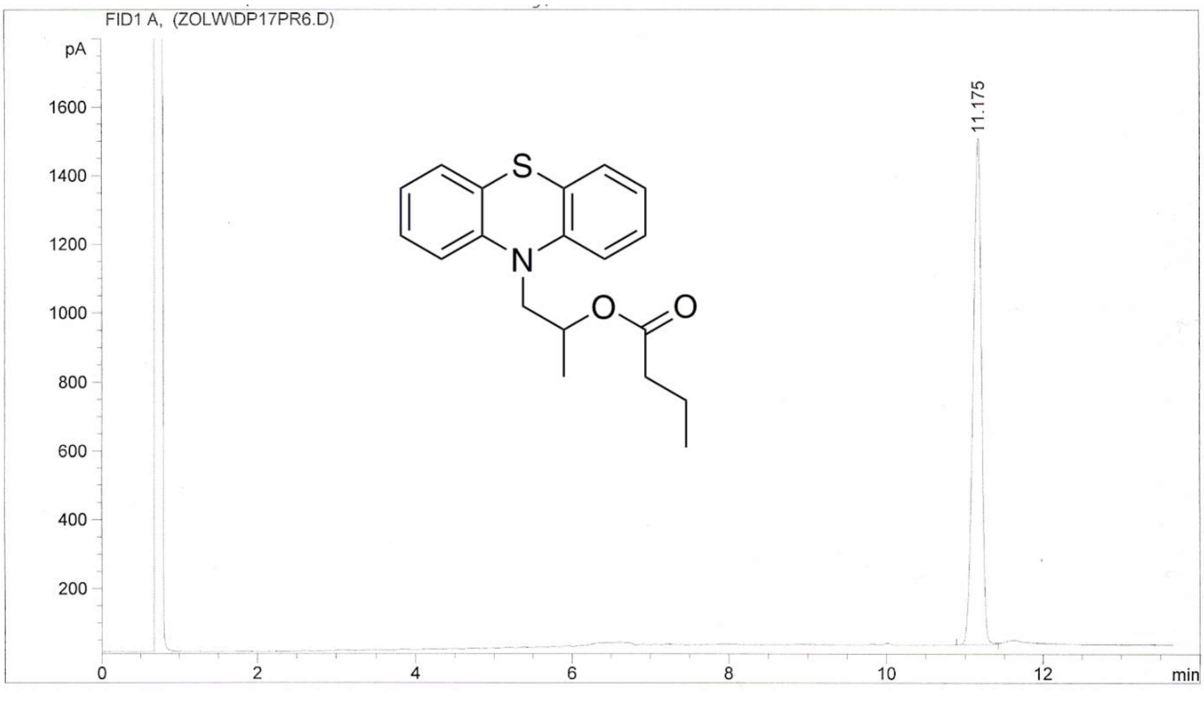

FID1 A, (ZOLW\DP17PR6.D)
pA
11.175
min
S
N
O
O

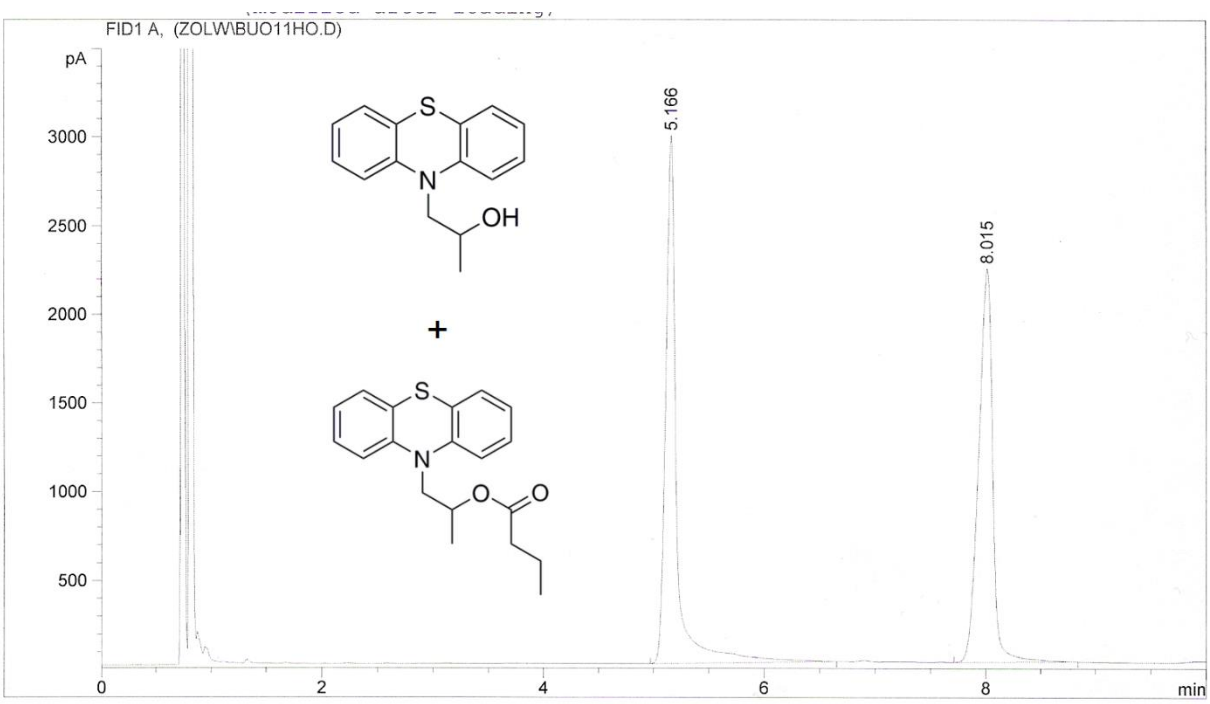

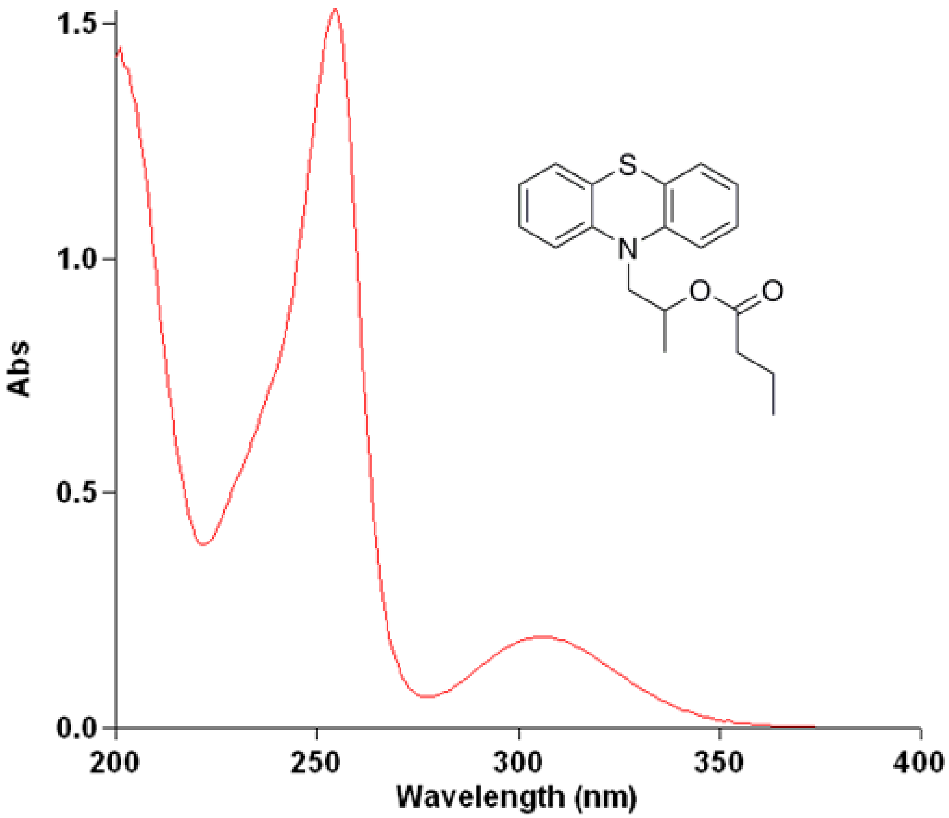

Wavelength (nm)	Abs
305.00	0.193
254.00	1.531
201.00	1.449

1-(10*H*-Phenothiazin-10-yl)propan-2-yl decanoate (±)-4c

Dissolved in CD_3OD and recorded on Varian Mercury 400BB spectrometer 400 MHz

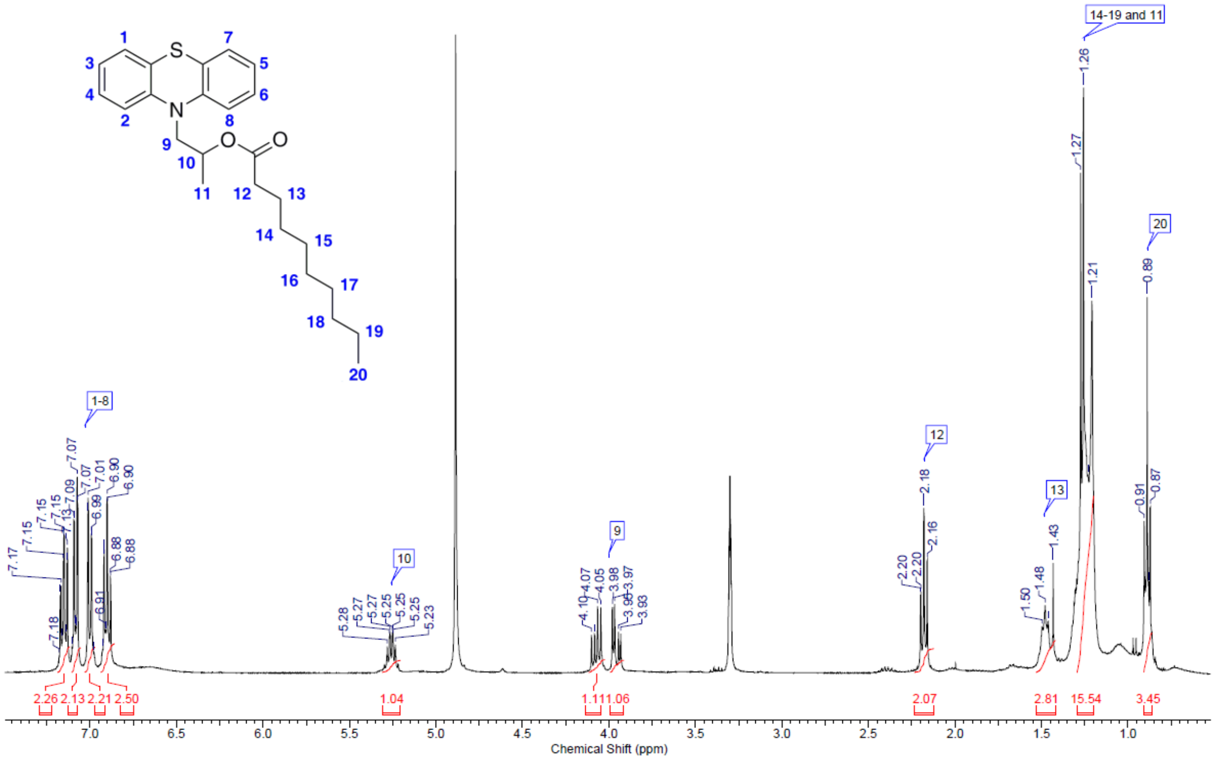

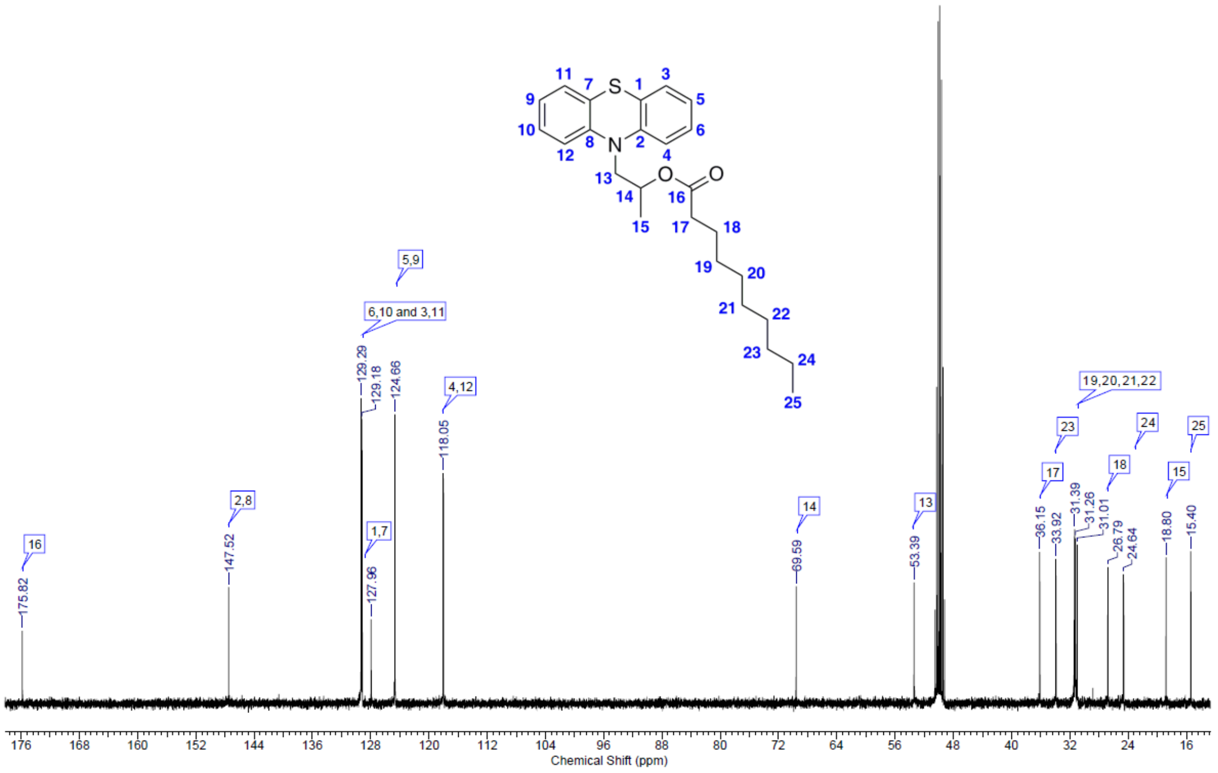

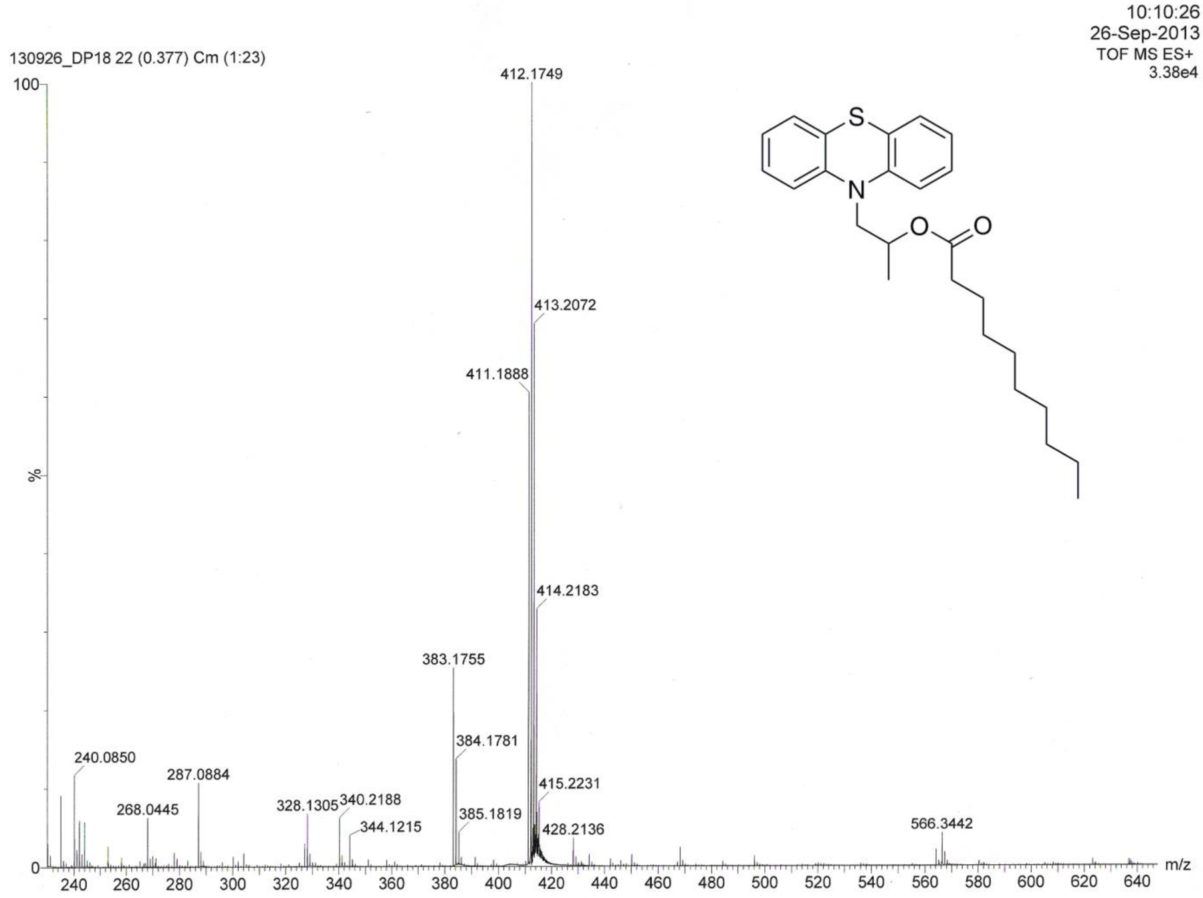
10:10:26
26-Sep-2013
TOF MS ES+
3.38e4
130926_DP18 22 (0.377) Cm (1:23)
412.1749
413.2072
411.1888
414.2183
383.1755
384.1781
240.0850
287.0884
268.0445
328.1305
340.2188
344.1215
385.1819
415.2231
428.2136
566.3442
%
m/z
S
N
O
O

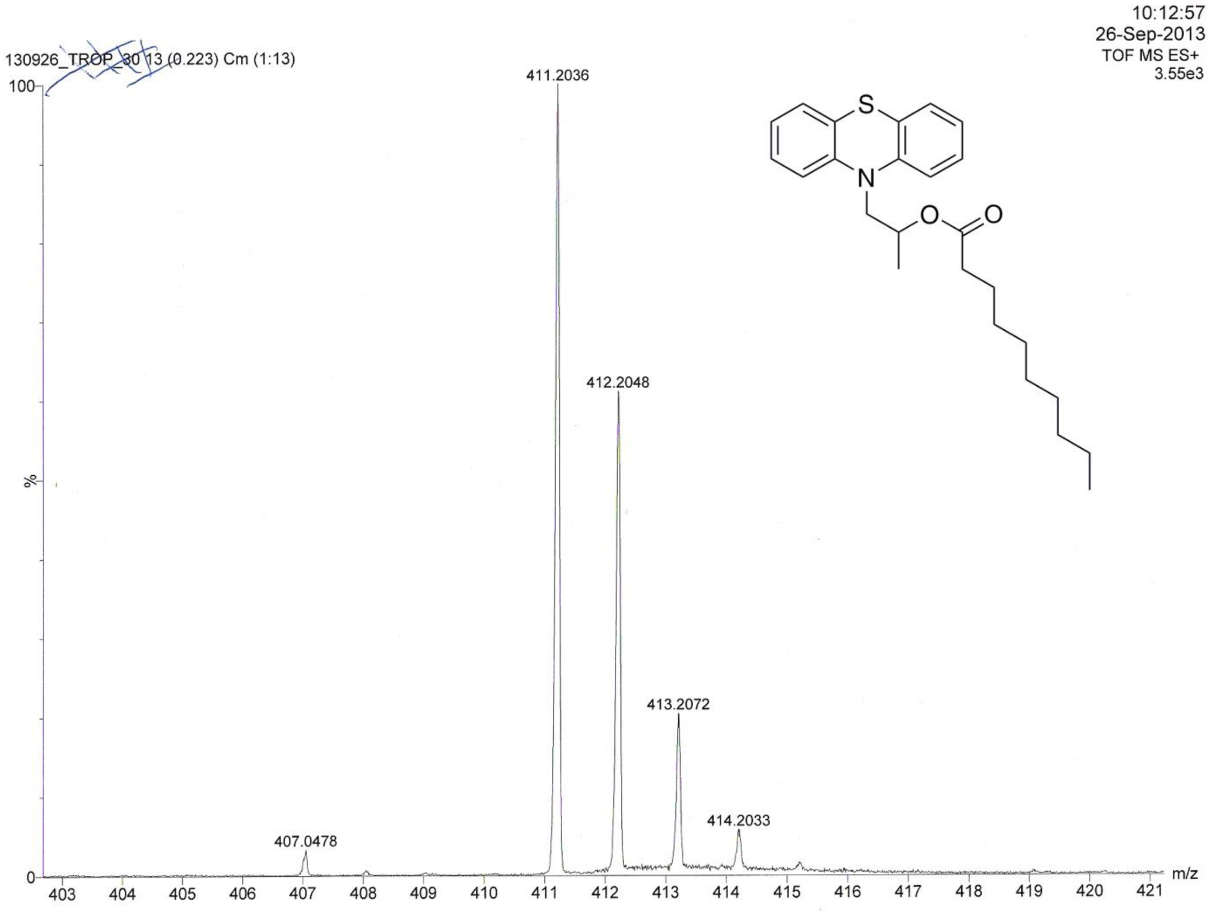
10:12:57
26-Sep-2013
TOF MS ES+
3.55e3
130926_TROP_30 13 (0.223) Cm (1:13)
411.2036
412.2048
413.2072
414.2033
407.0478
%
m/z
S
N
O
O

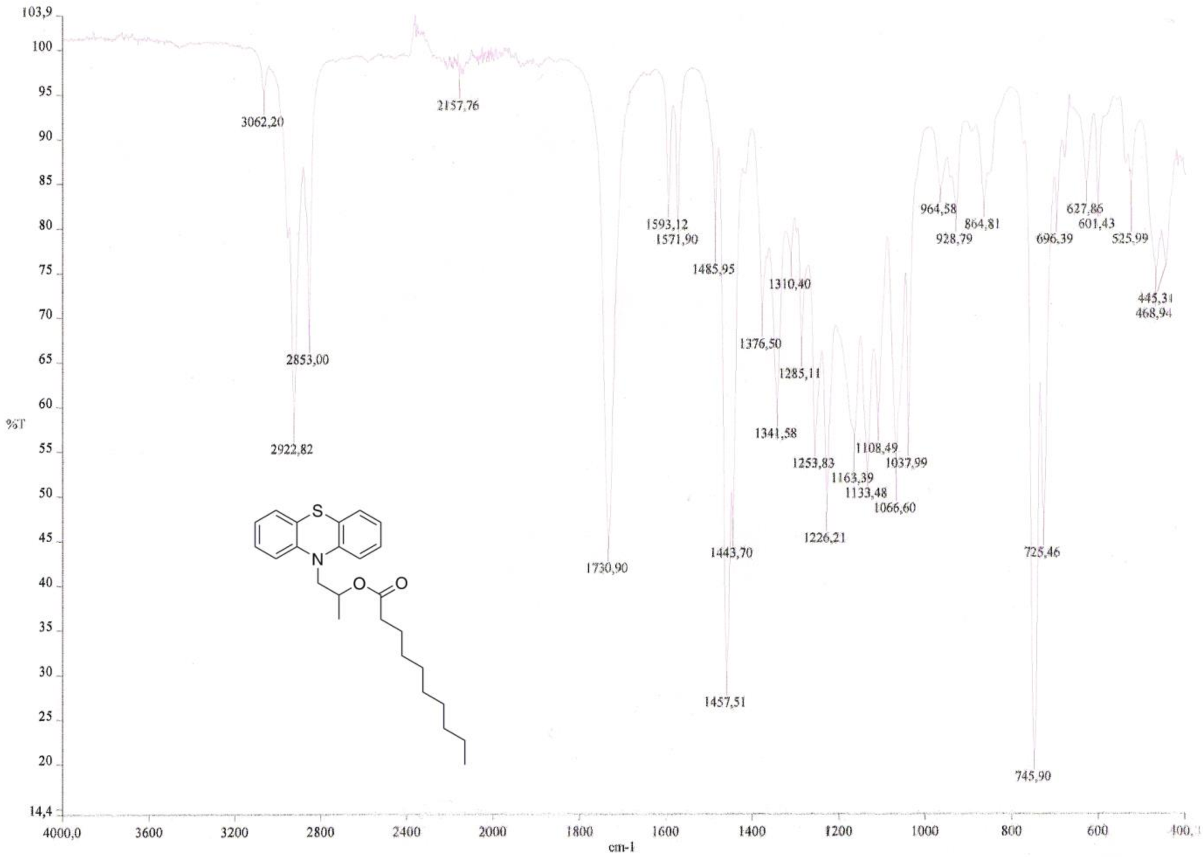

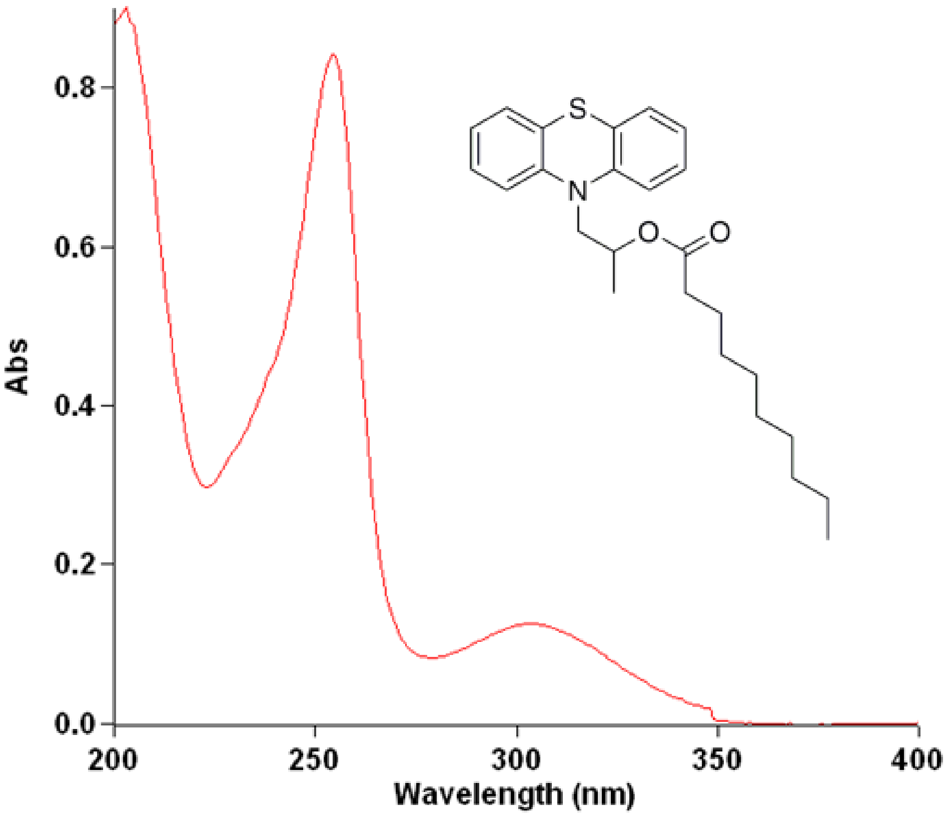

Wavelength (nm)	Abs
304.00	0.126
254.00	0.842
203.00	0.901

(*R*)-MPA ester 11

Dissolved in C_6D_6 and recorded on Varian Mercury 400BB spectrometer 400 MHz

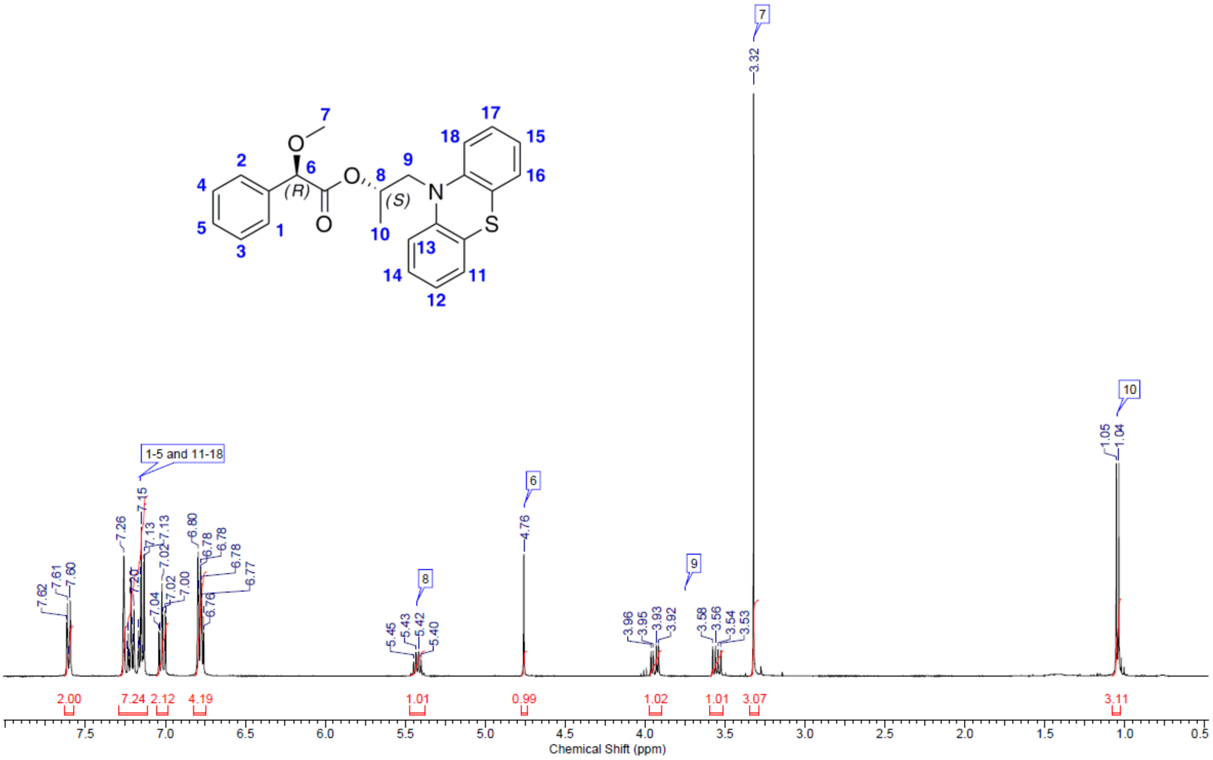

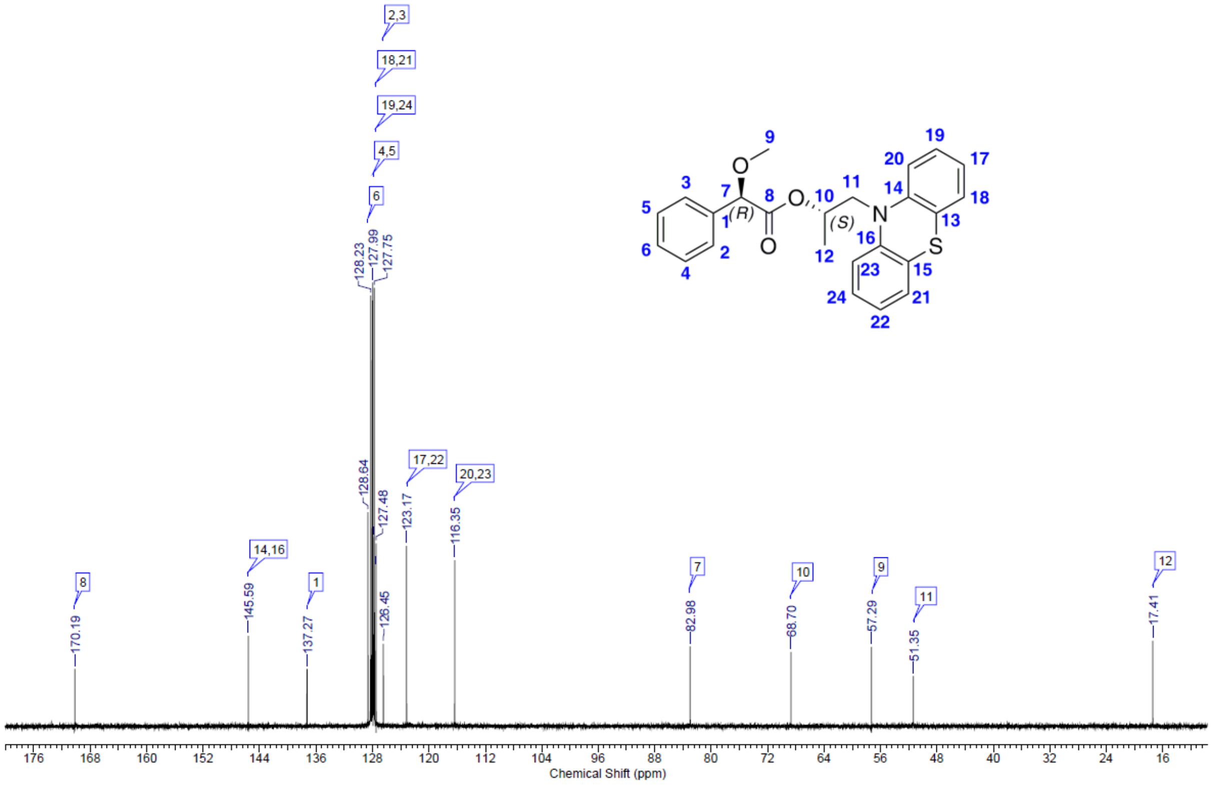

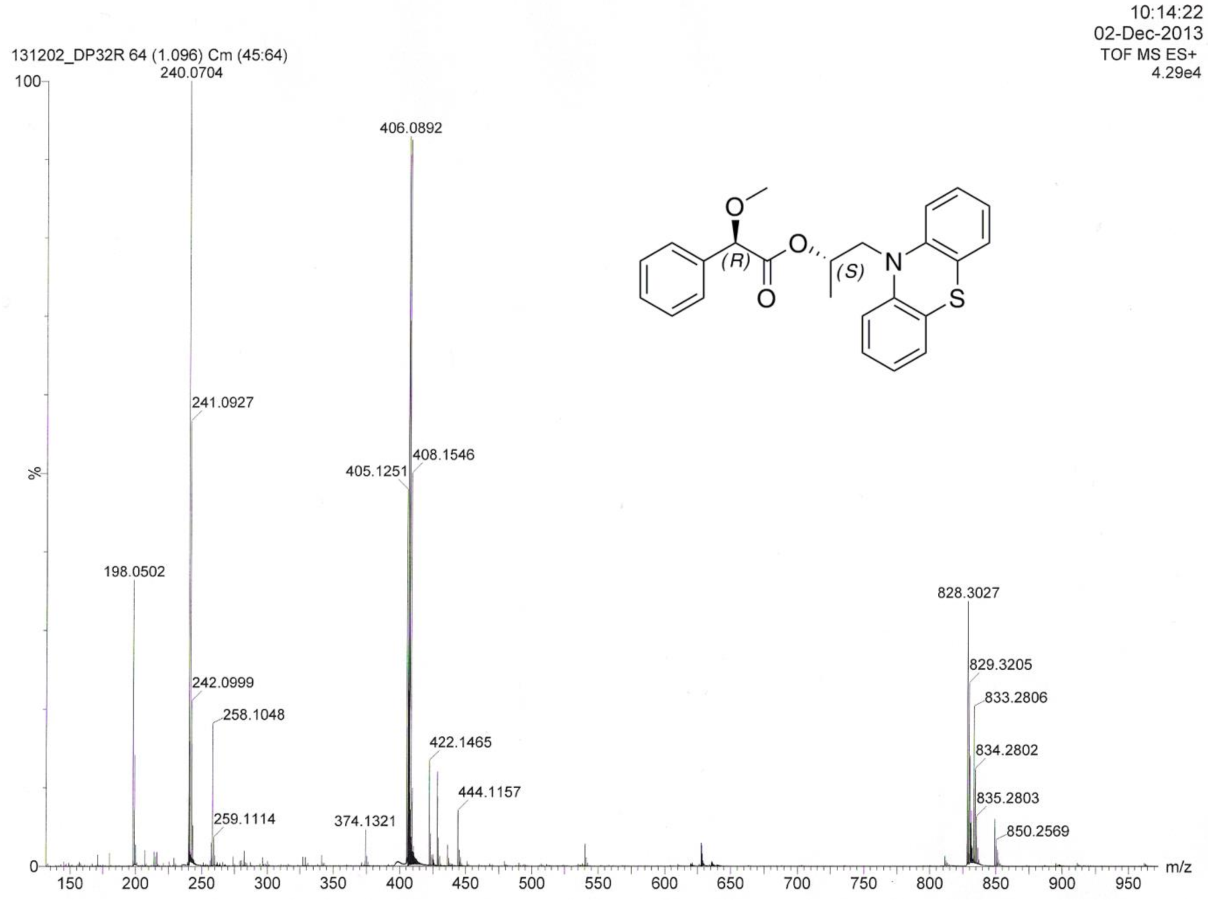

10:14:22
02-Dec-2013
TOF MS ES+
4.29e4
131202_DP32R 64 (1.096) Cm (45:64)
240.0704
406.0892
241.0927
405.1251
408.1546
198.0502
828.3027
829.3205
833.2806
834.2802
835.2803
850.2569
242.0999
258.1048
422.1465
444.1157
374.1321
259.1114
(R)
(S)
O
N
S
m/z
%

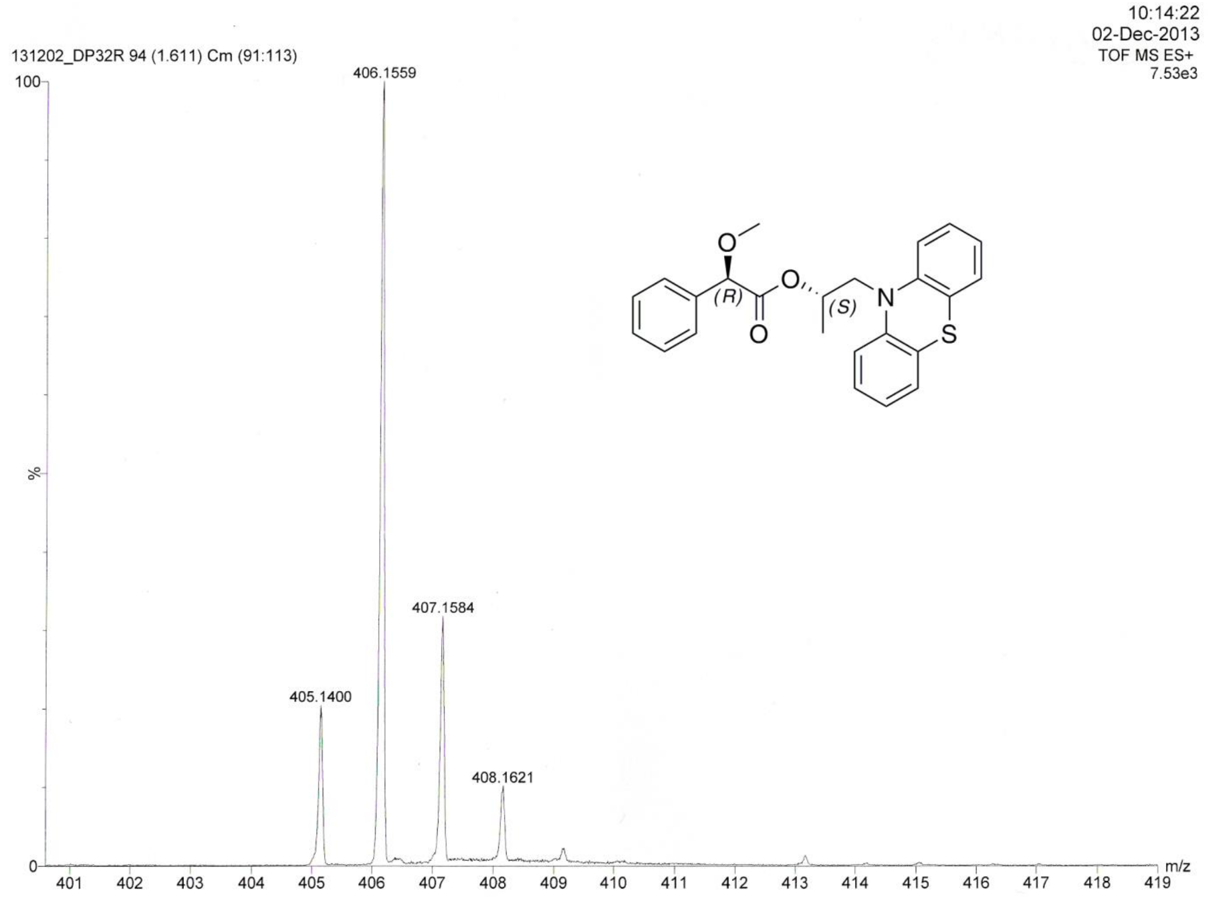

10:14:22
02-Dec-2013
TOF MS ES+
7.53e3
131202_DP32R 94 (1.611) Cm (91:113)
406.1559
407.1584
405.1400
408.1621
(R)
(S)
O
N
S
m/z
%

(*S*)-MPA ester 12

Dissolved in C_6D_6 and recorded on Varian Mercury 400BB spectrometer 400 MHz

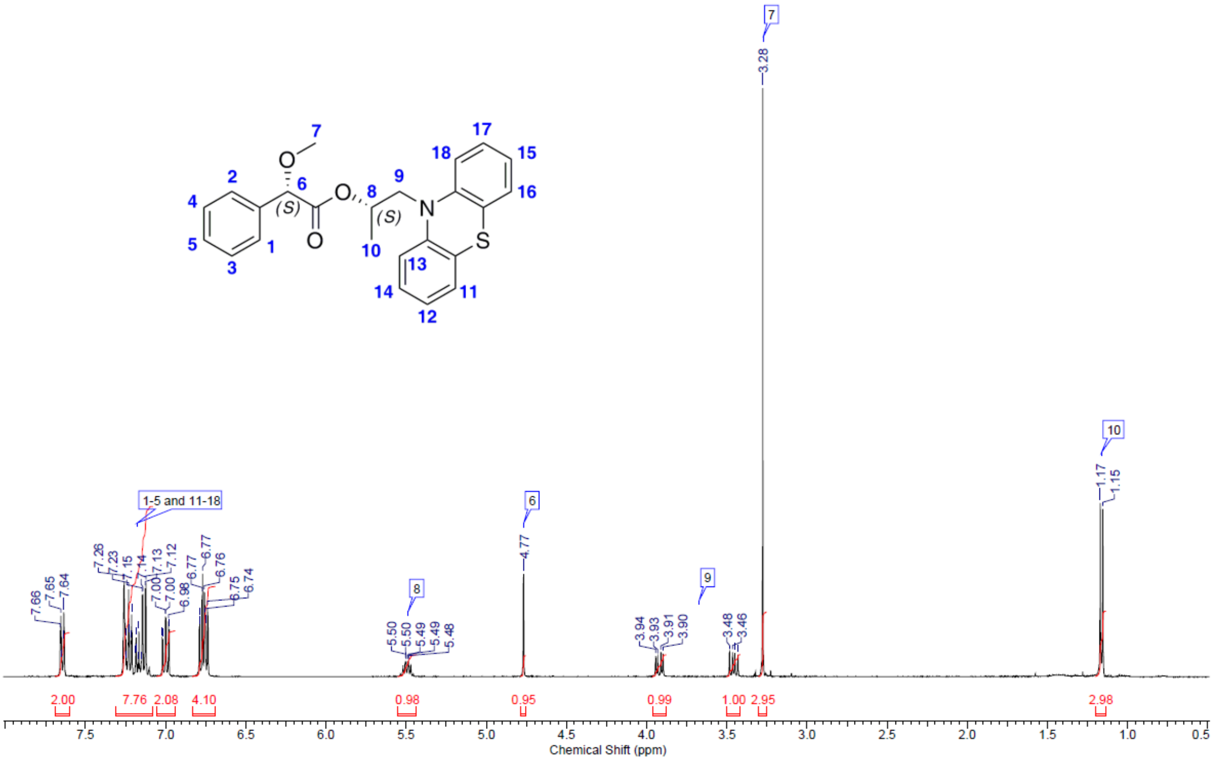

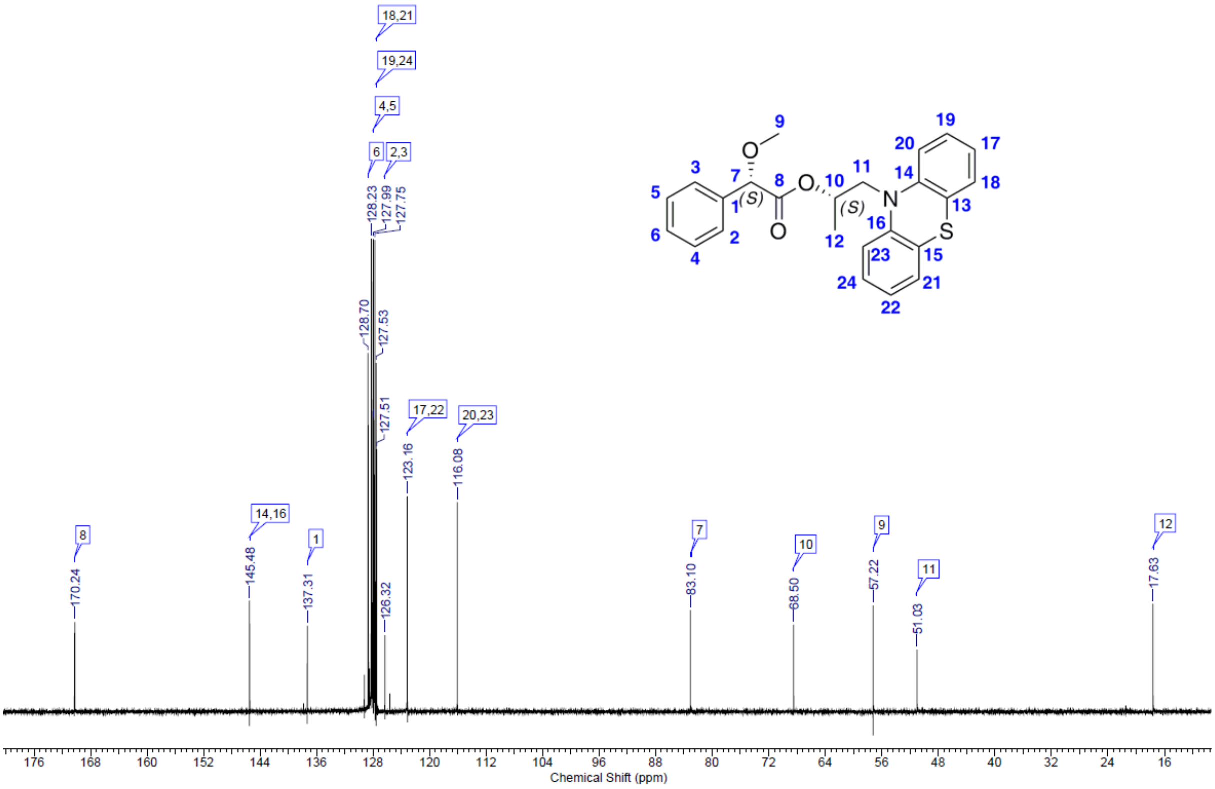

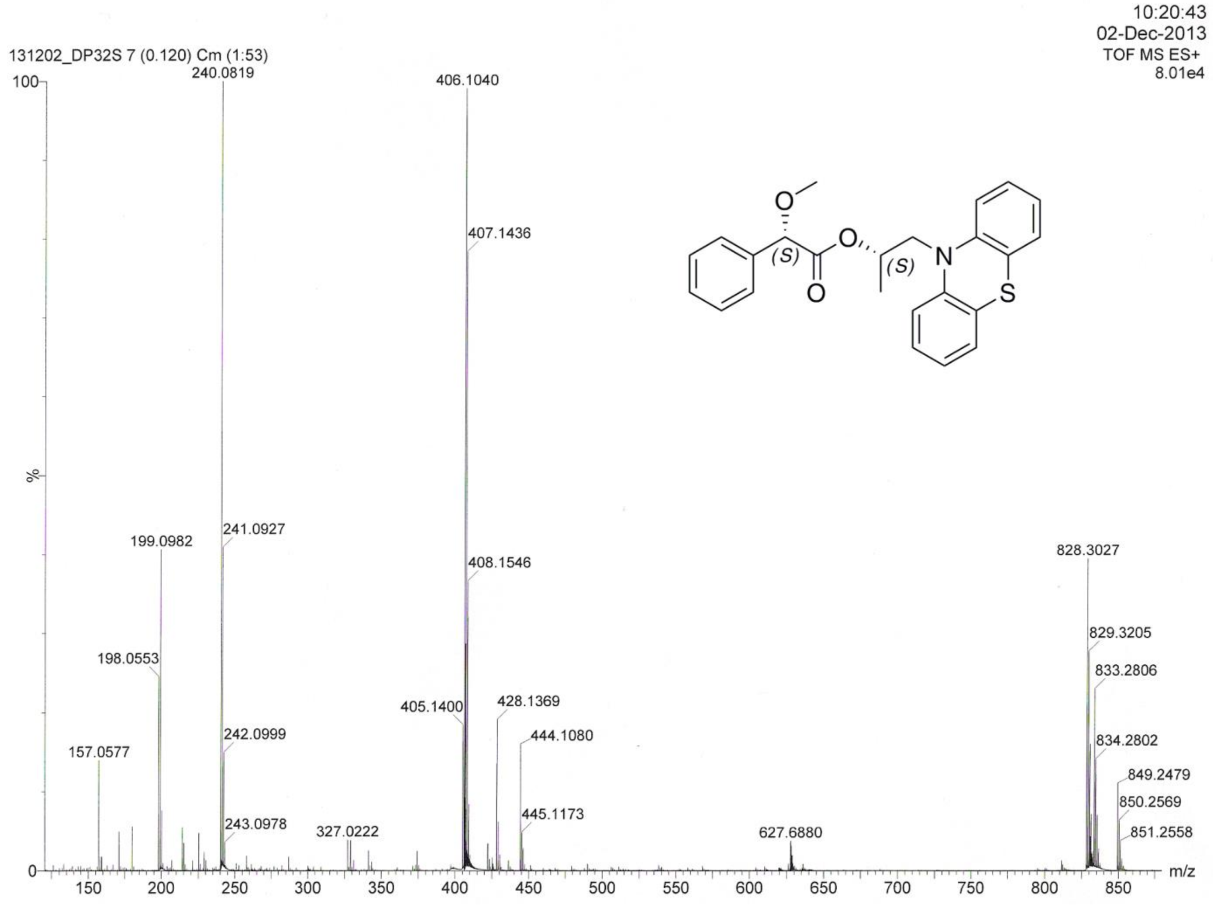
10:20:43
02-Dec-2013
TOF MS ES+
8.01e4
131202_DP32S 7 (0.120) Cm (1:53)
240.0819
406.1040
407.1436
199.0982
241.0927
408.1546
828.3027
829.3205
833.2806
198.0553
405.1400
428.1369
444.1080
834.2802
157.0577
242.0999
849.2479
850.2569
243.0978
327.0222
445.1173
627.6880
851.2558
100
%
0
m/z
(S)
(S)
O
N
S

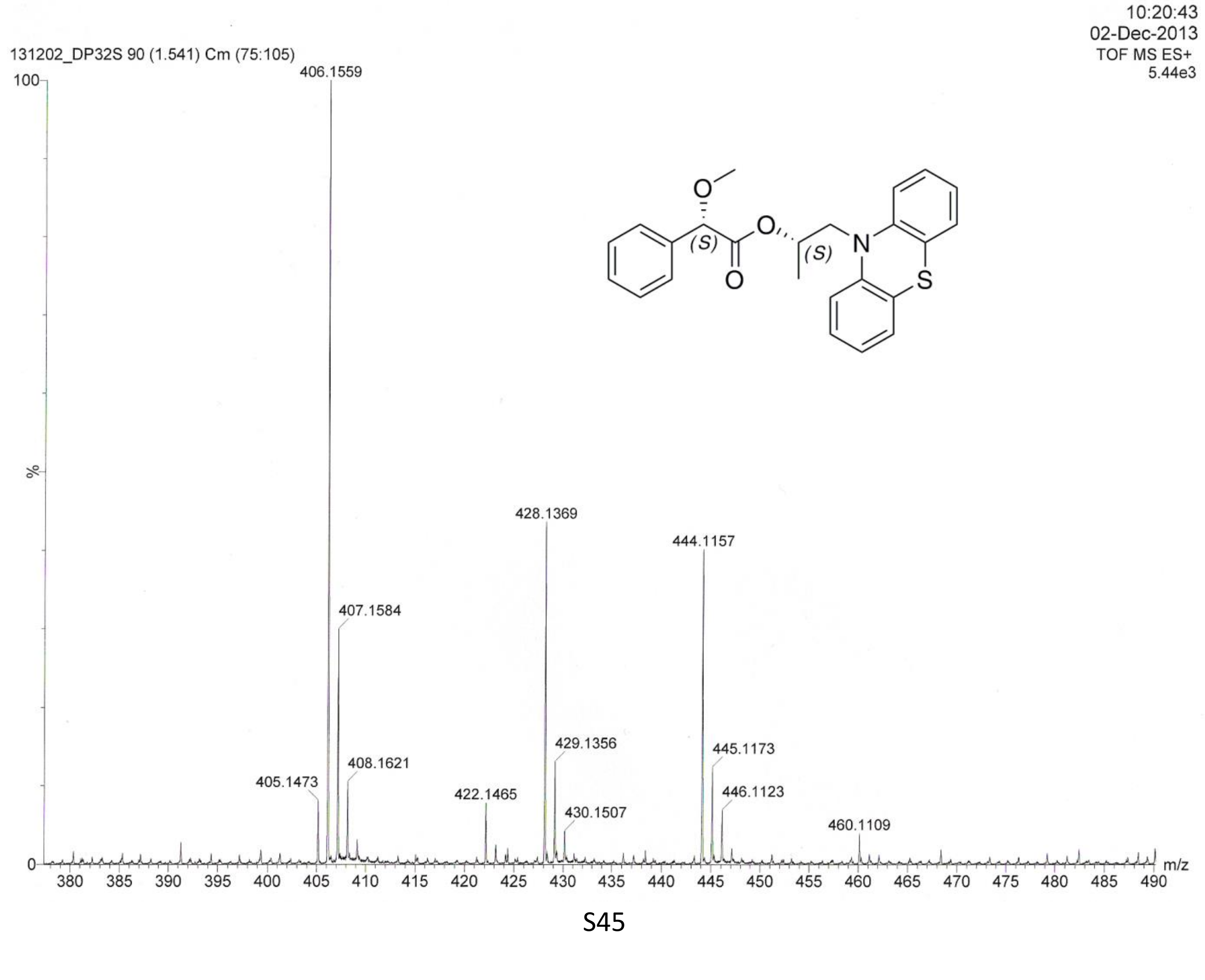
10:20:43
02-Dec-2013
TOF MS ES+
5.44e3
131202_DP32S 90 (1.541) Cm (75:105)
406.1559
428.1369
444.1157
407.1584
429.1356
445.1173
408.1621
405.1473
422.1465
446.1123
430.1507
460.1109
100
%
0
m/z
(S)
(S)
O
N
S

10-[2-Bromopropyl]-10*H*-phenothiazine (±)-8

Dissolved in C_6D_6 and recorded on Varian Mercury 400BB spectrometer 400 MHz

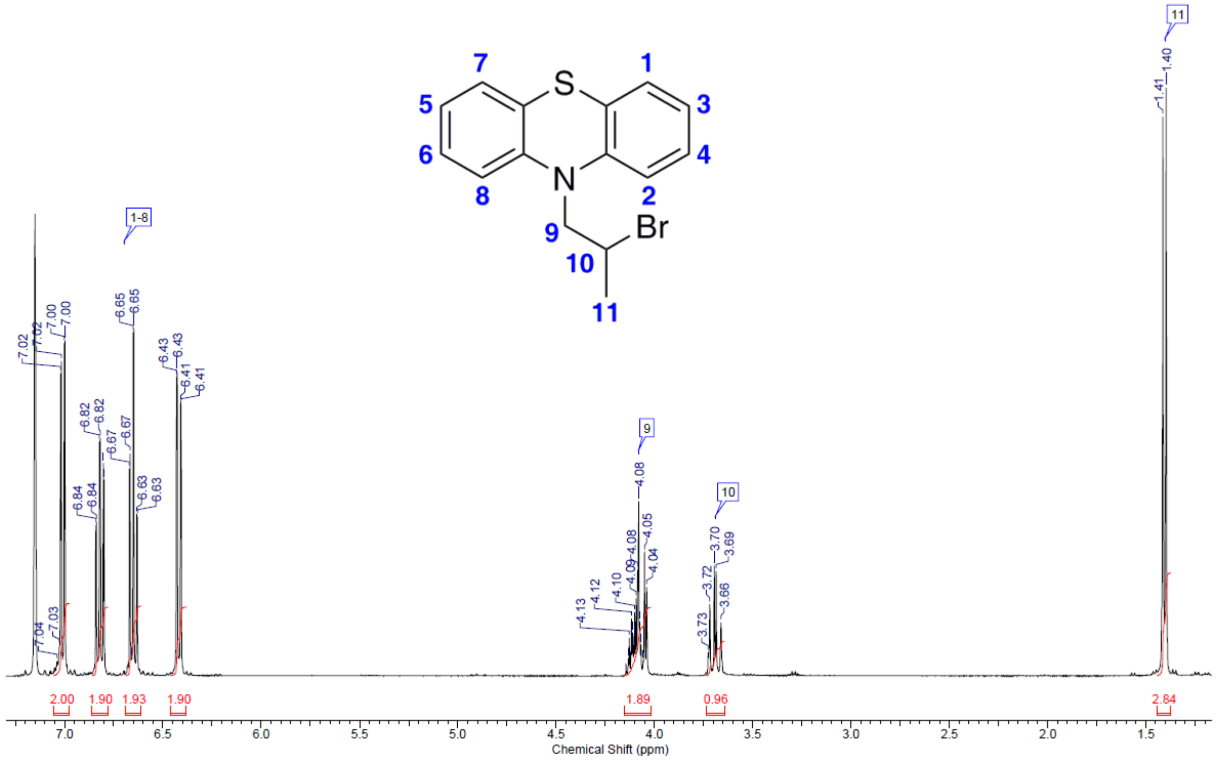

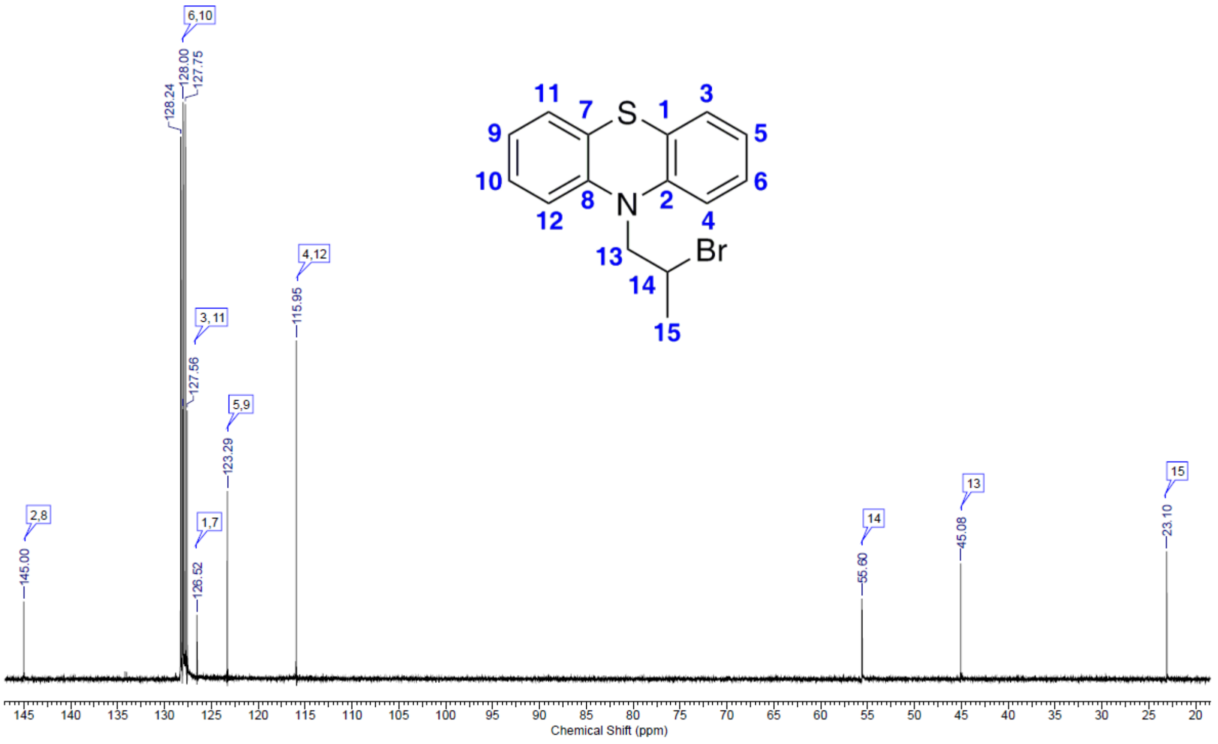

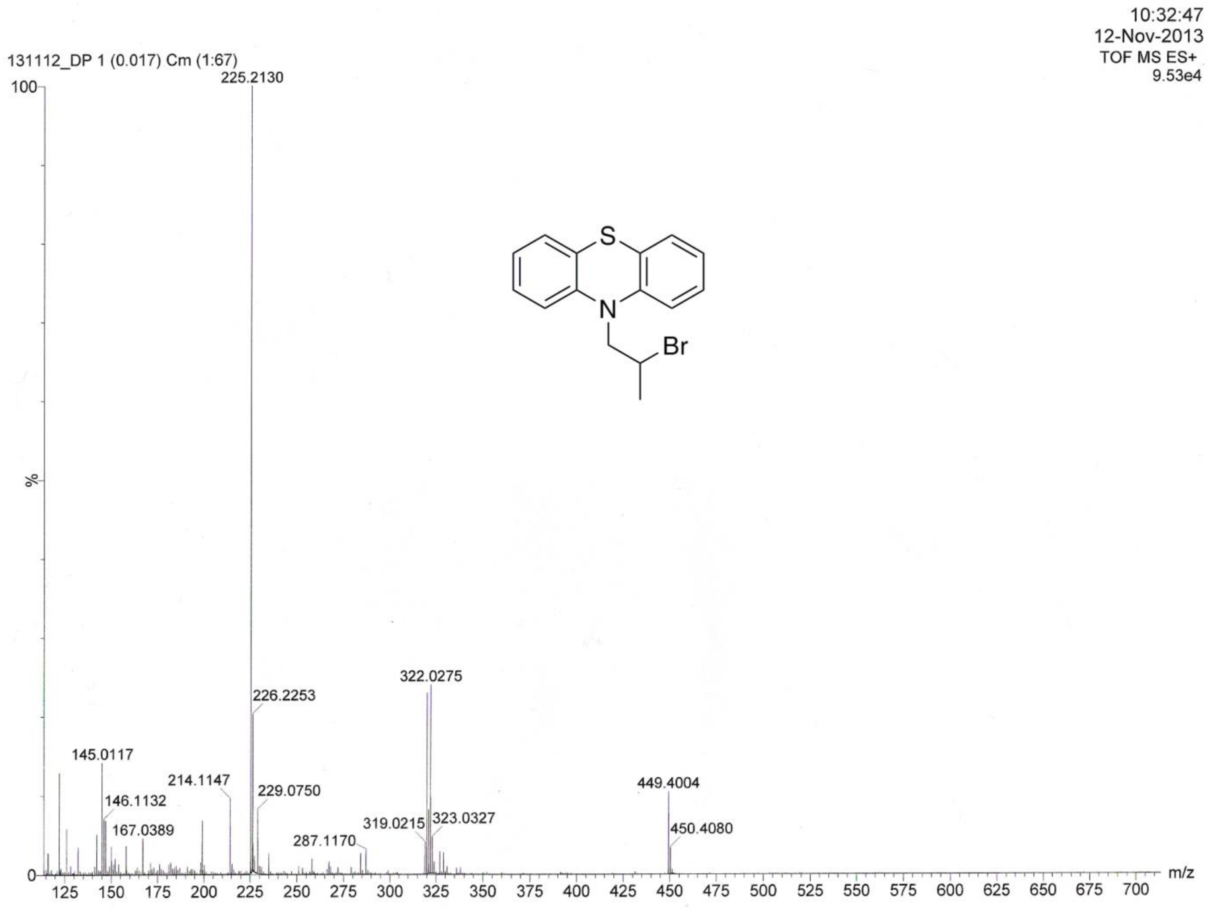

10:32:47
12-Nov-2013
TOF MS ES+
9.53e4
131112_DP 1 (0.017) Cm (1:67)
225.2130
226.2253
322.0275
145.0117
214.1147
229.0750
146.1132
167.0389
287.1170
319.0215
323.0327
449.4004
450.4080
S
N
Br
%
m/z

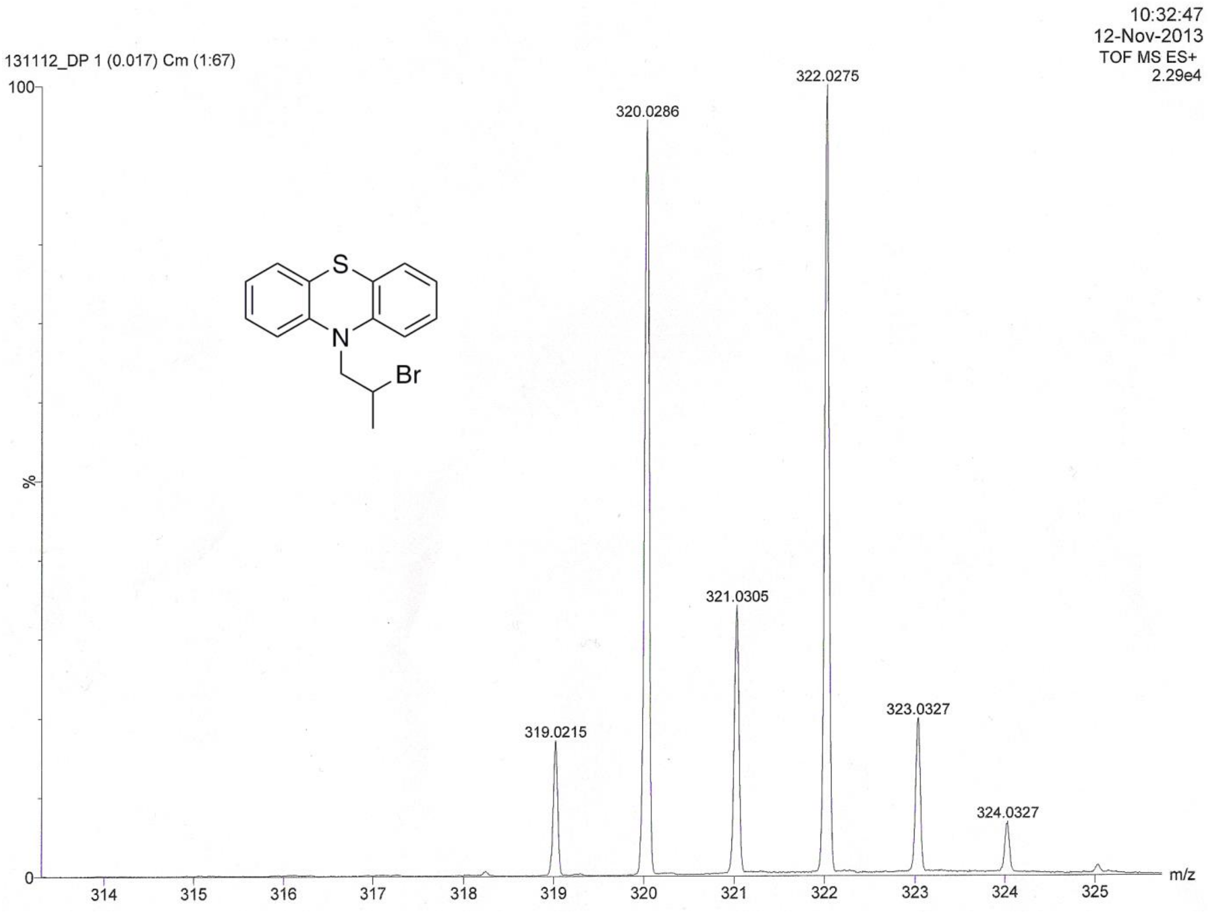

10:32:47
12-Nov-2013
TOF MS ES+
2.29e4
131112_DP 1 (0.017) Cm (1:67)
322.0275
320.0286
321.0305
323.0327
319.0215
324.0327
S
N
Br
%
m/z

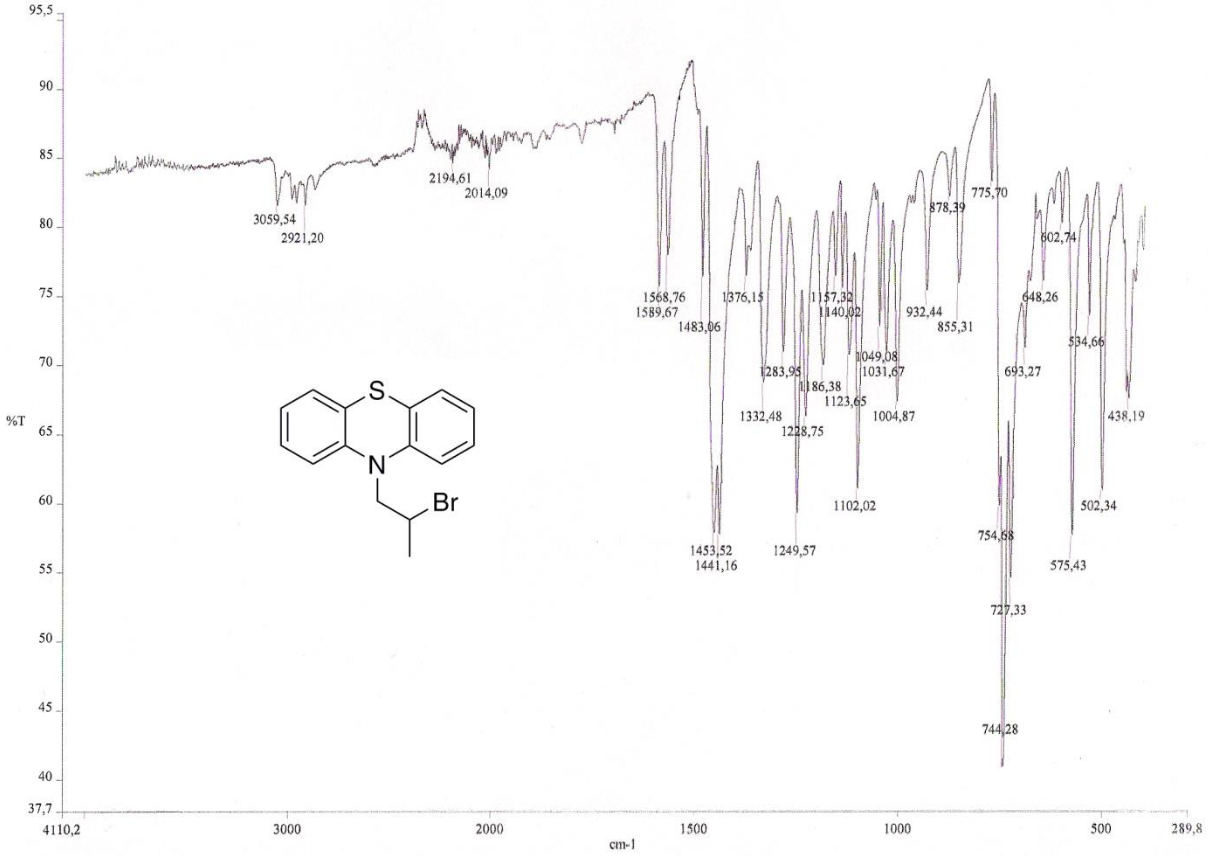

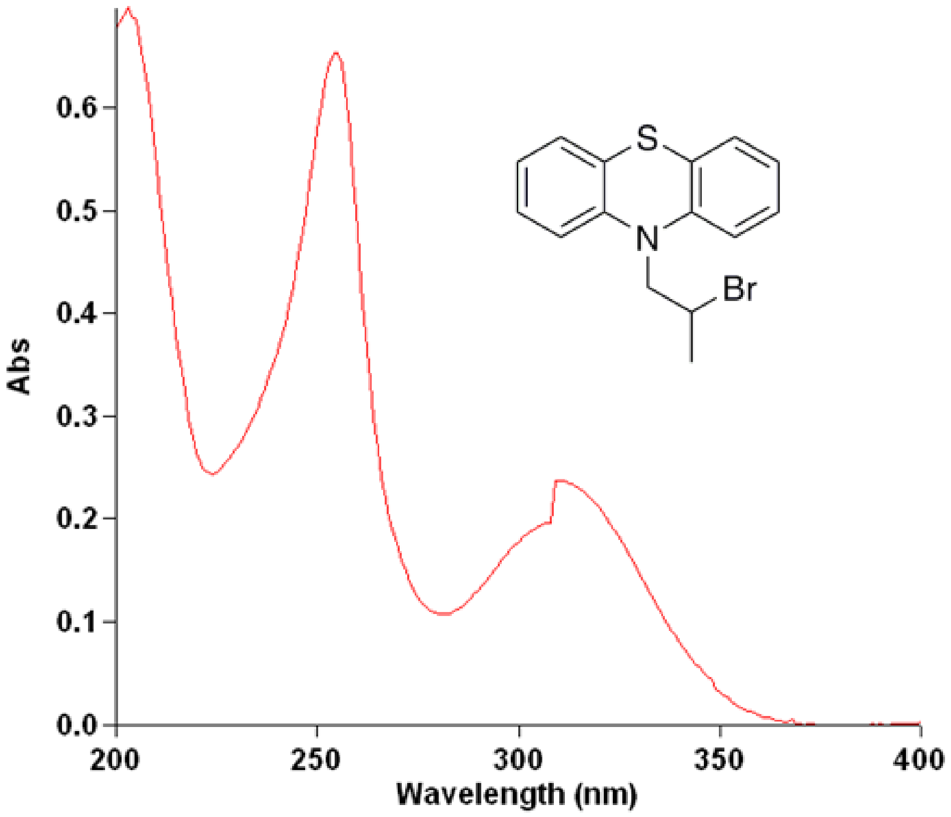

Wavelength (nm)	Abs
309.00	0.237
255.00	0.654
203.00	0.697

***N*,*N*-Dimethyl-1-(10*H*-phenothiazin-10-yl)propan-2-amine (promethazine) (±)-9**

Dissolved in C_6D_6 and recorded on Varian Mercury 400BB spectrometer 400 MHz

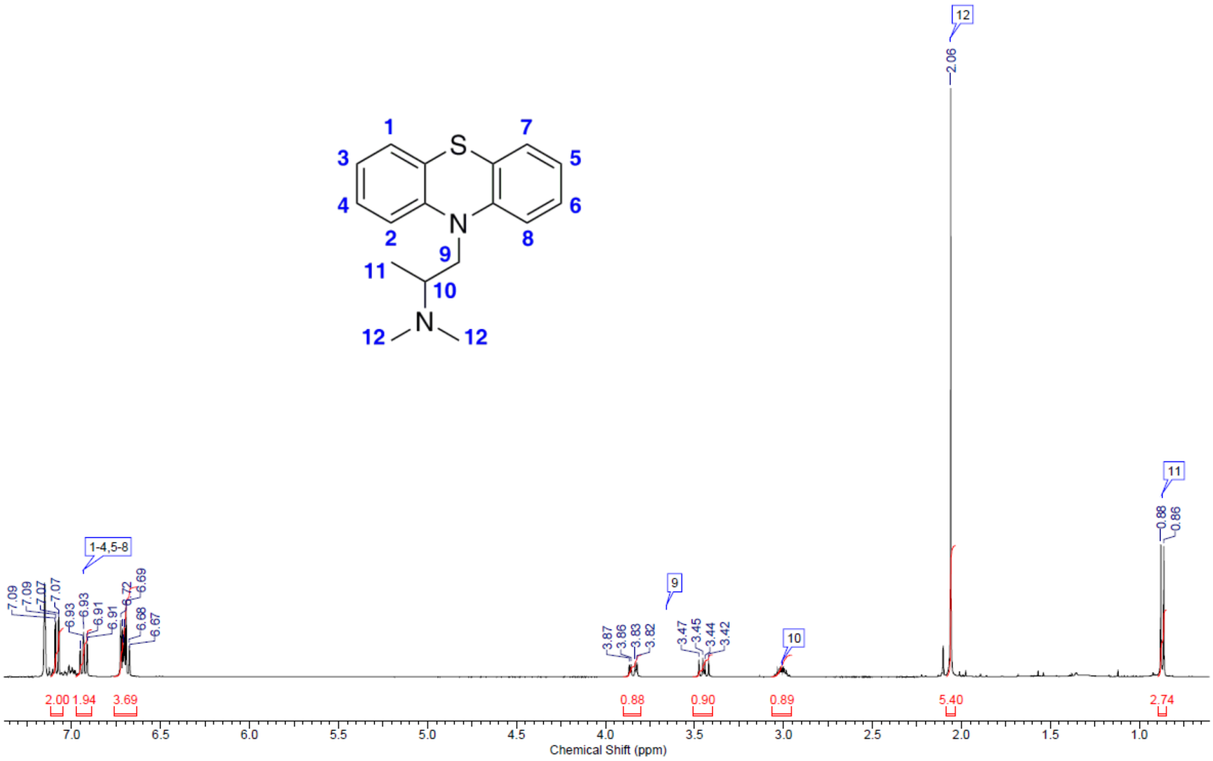

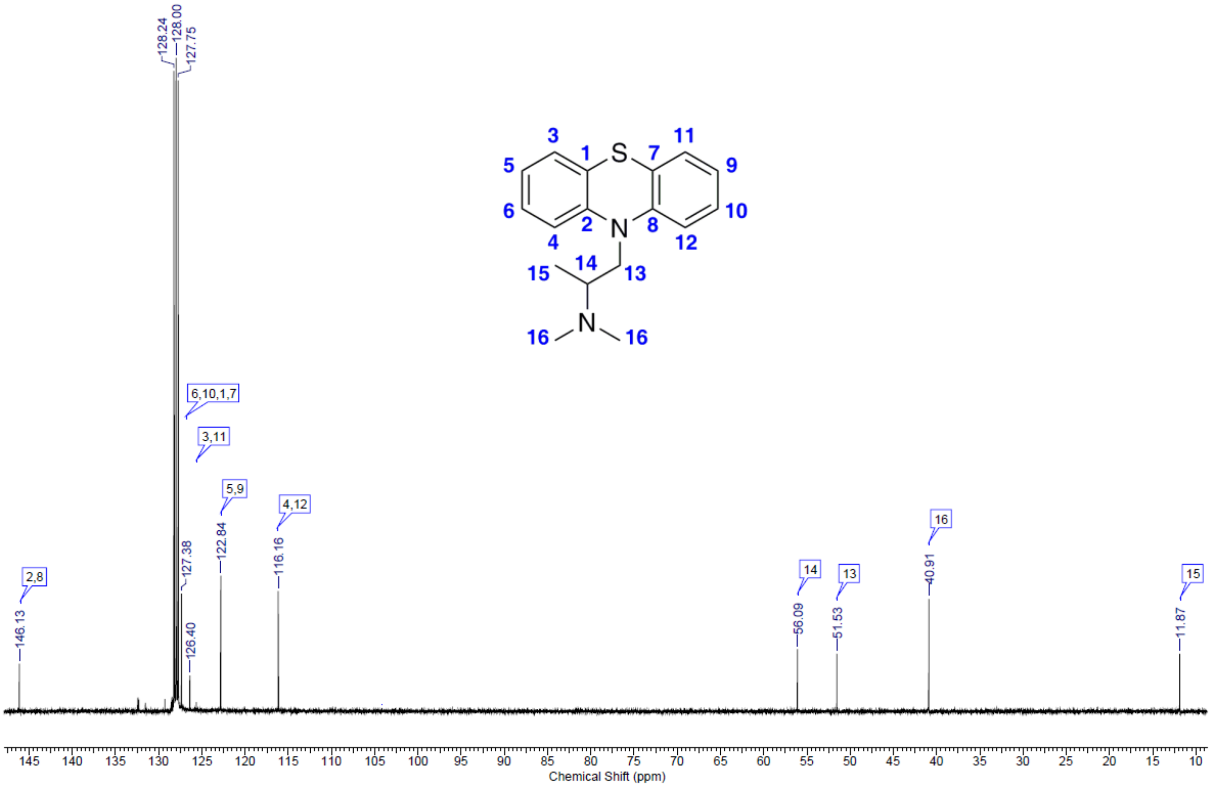

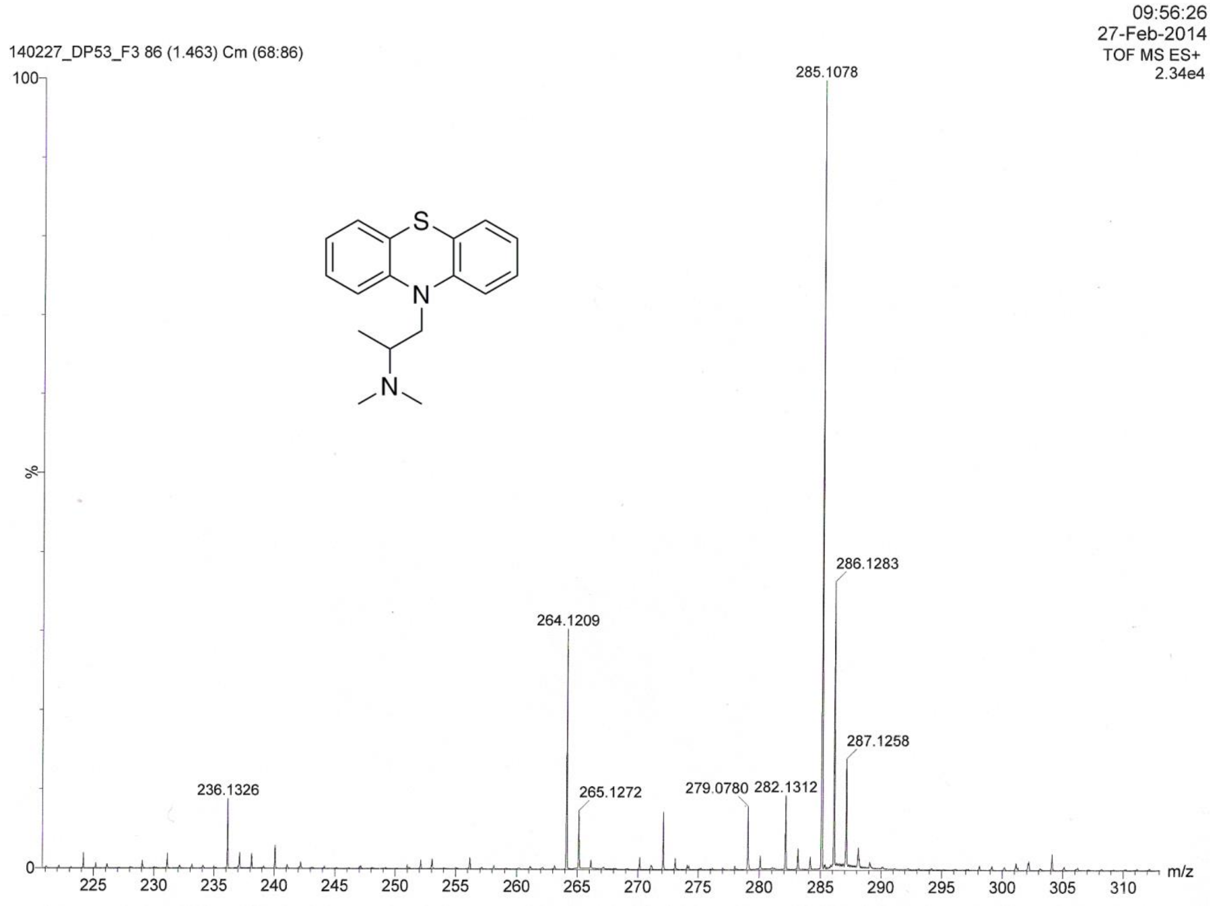
09:56:26
27-Feb-2014
TOF MS ES+
2.34e4
140227_DP53_F3 86 (1.463) Cm (68:86)
285.1078
286.1283
264.1209
287.1258
236.1326
265.1272
279.0780
282.1312
%
m/z
S
N

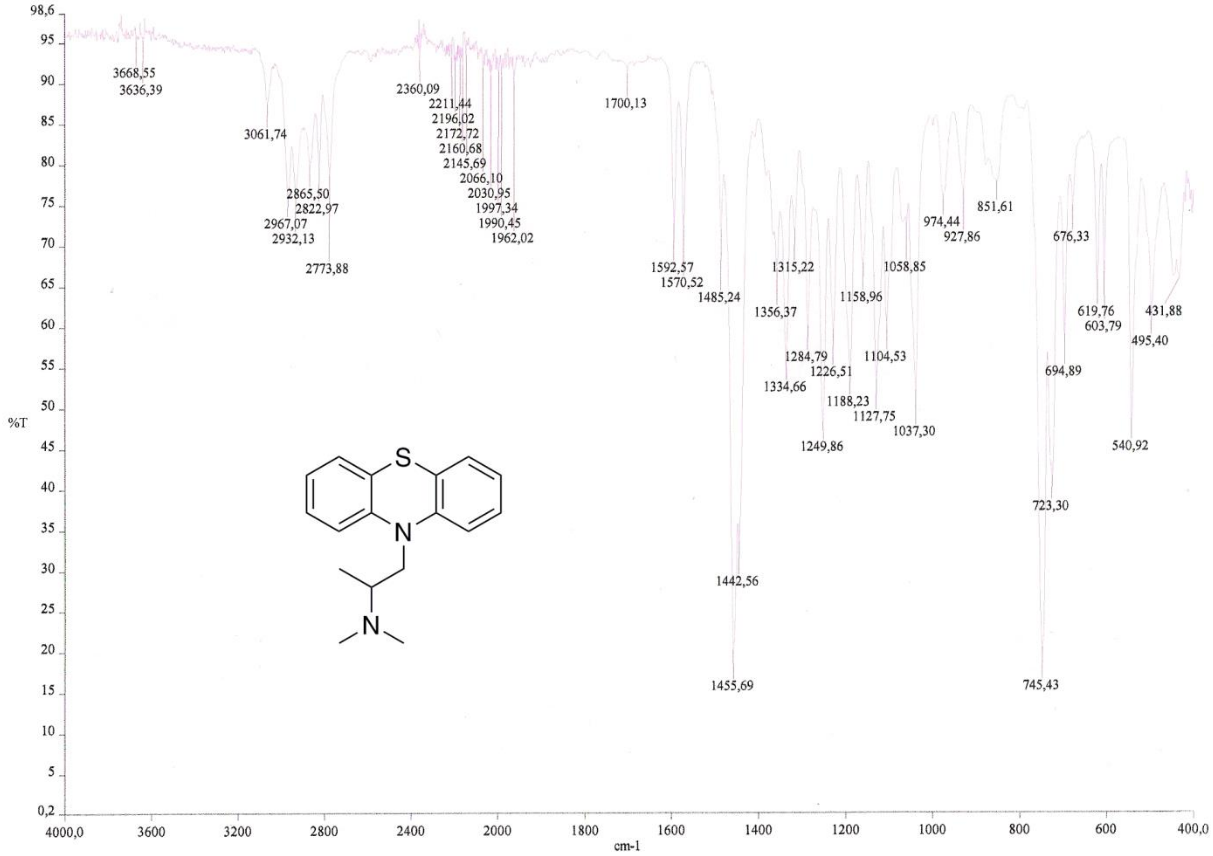
%T
cm-1
3668,55
3636,39
3061,74
2865,50
2822,97
2967,07
2932,13
2773,88
2360,09
2211,44
2196,02
2172,72
2160,68
2145,69
2066,10
2030,95
1997,34
1990,45
1962,02
1700,13
1592,57
1570,52
1485,24
1442,56
1455,69
1315,22
1356,37
1284,79
1226,51
1334,66
1188,23
1127,75
1249,86
1158,96
1104,53
1058,85
1037,30
974,44
927,86
851,61
676,33
694,89
723,30
745,43
619,76
603,79
431,88
495,40
540,92
S
N

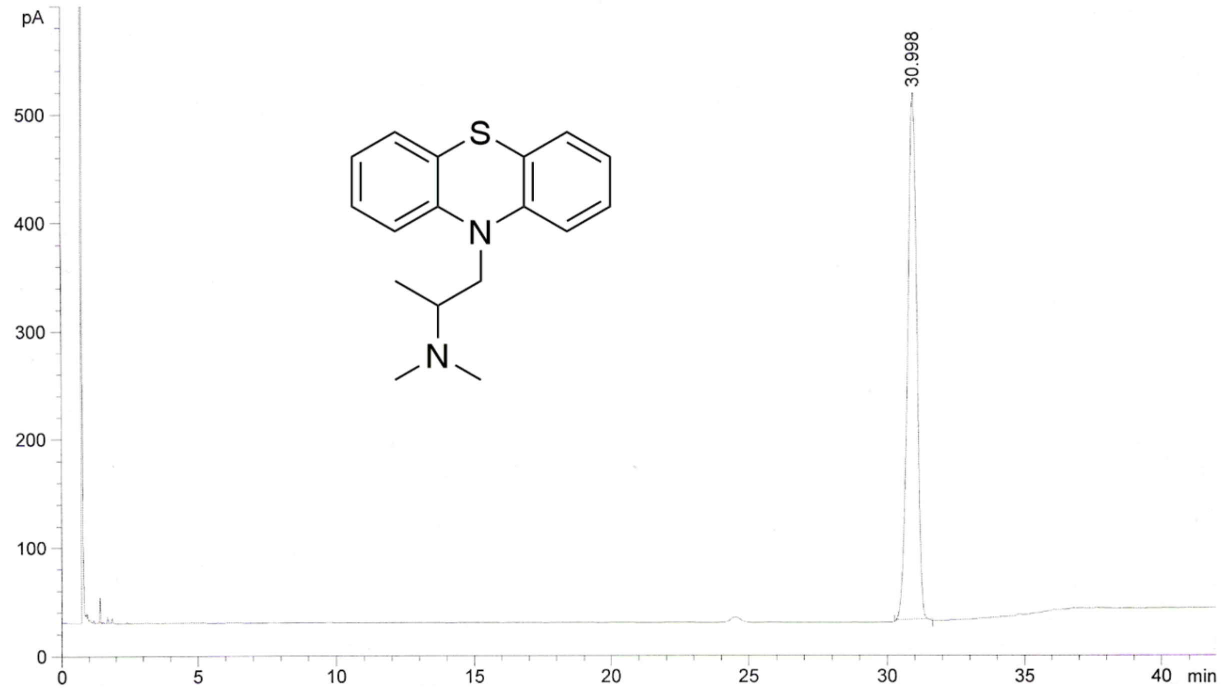

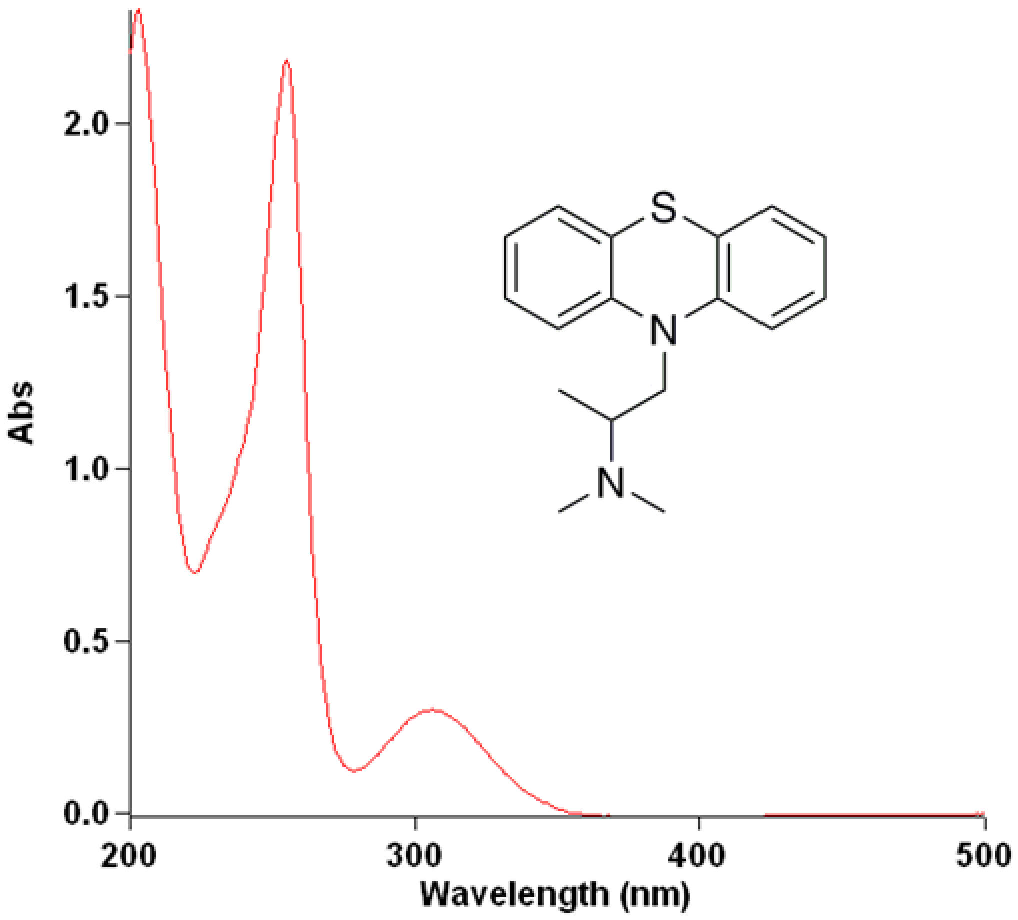

Wavelength (nm)	Abs
306.00	0.302
255.00	2.185
203.00	2.334

***N*,*N*-Diethyl-1-(10*H*-phenothiazin-10-yl)propan-2-amine (ethopropazine) (±)-10**

Dissolved in CD_3OD and recorded on Varian Mercury 400BB spectrometer 400 MHz

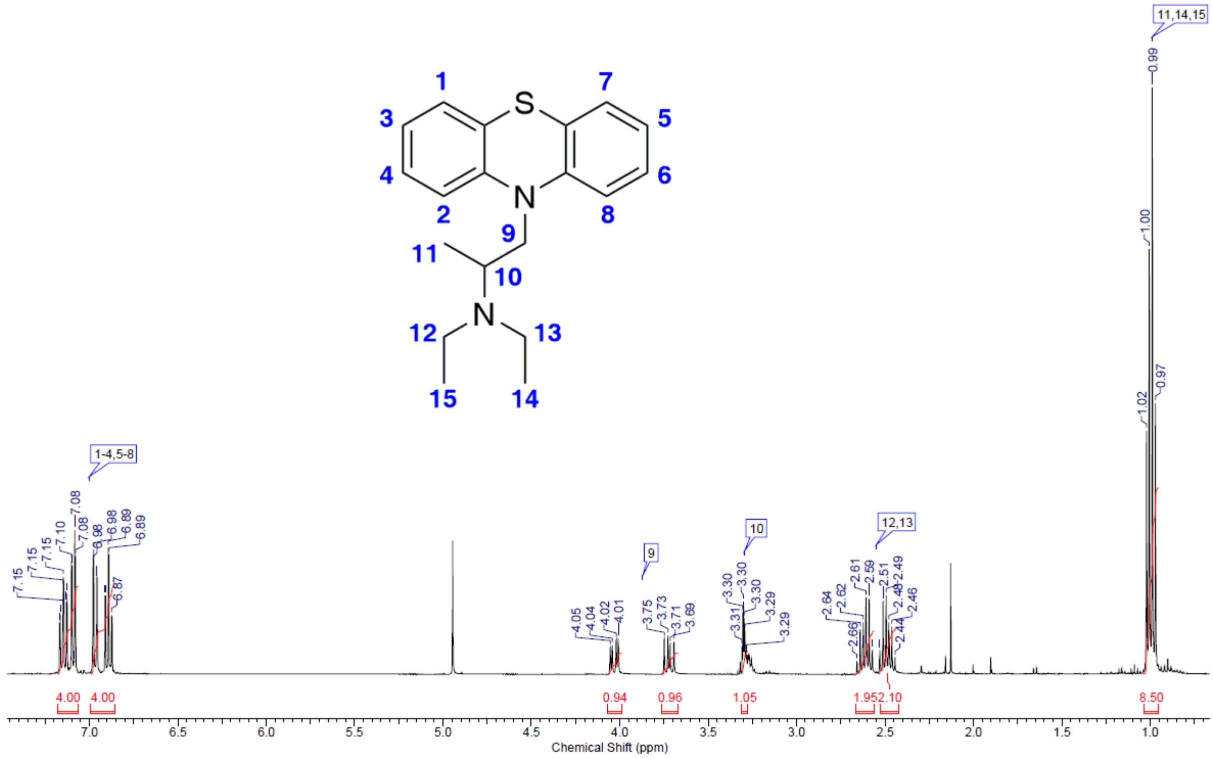

Dissolved in C_6D_6 and recorded on Varian Mercury 400BB spectrometer 400 MHz

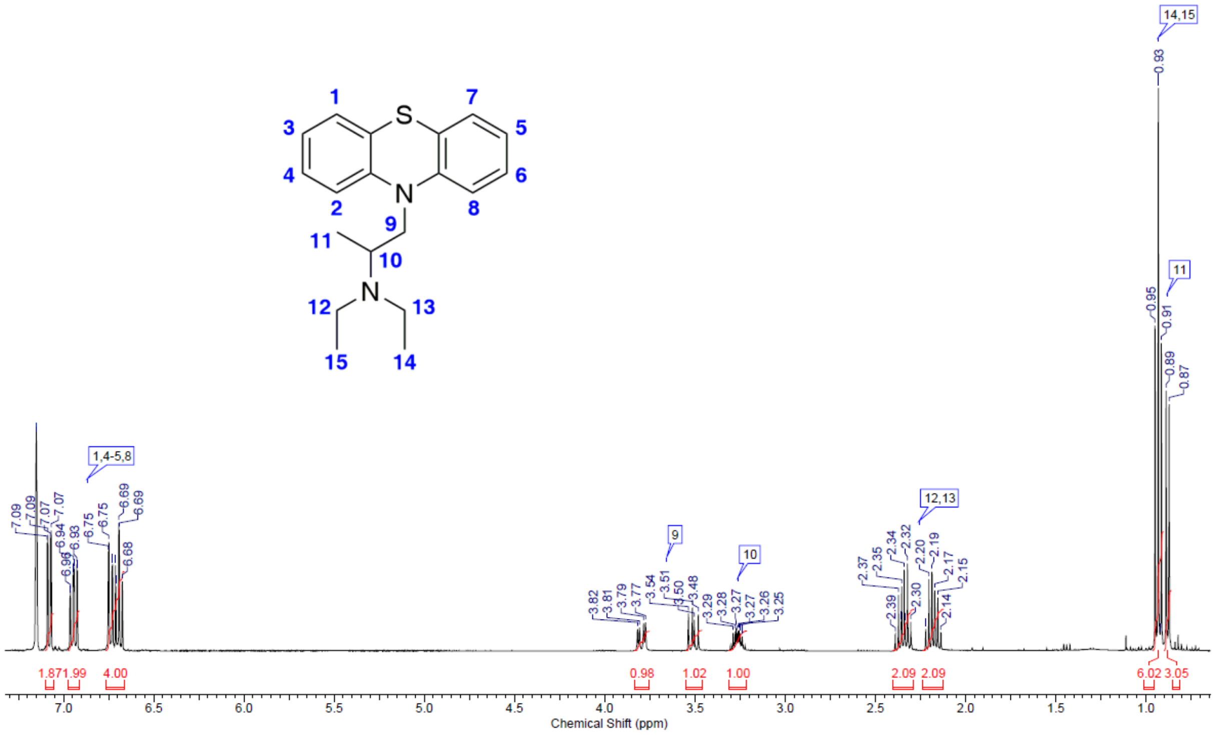

Dissolved in CD_3OD and recorded on Varian Mercury 400BB spectrometer 400 MHz

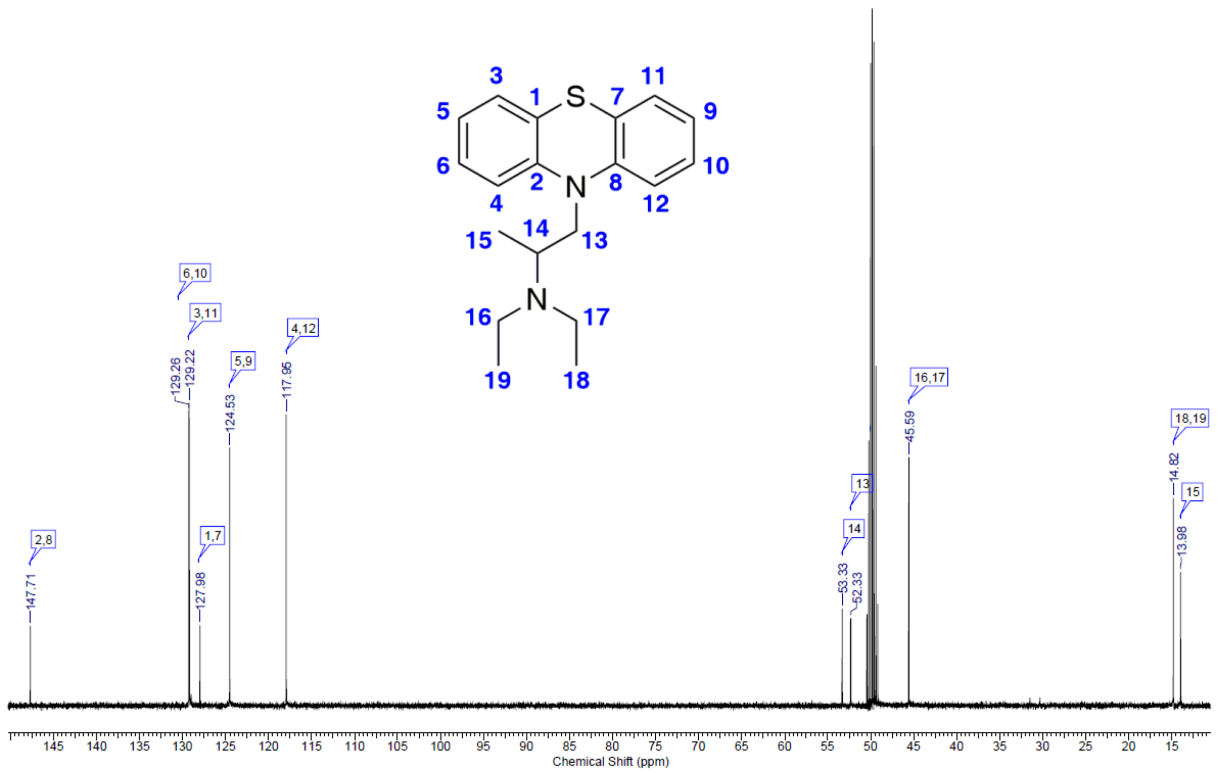

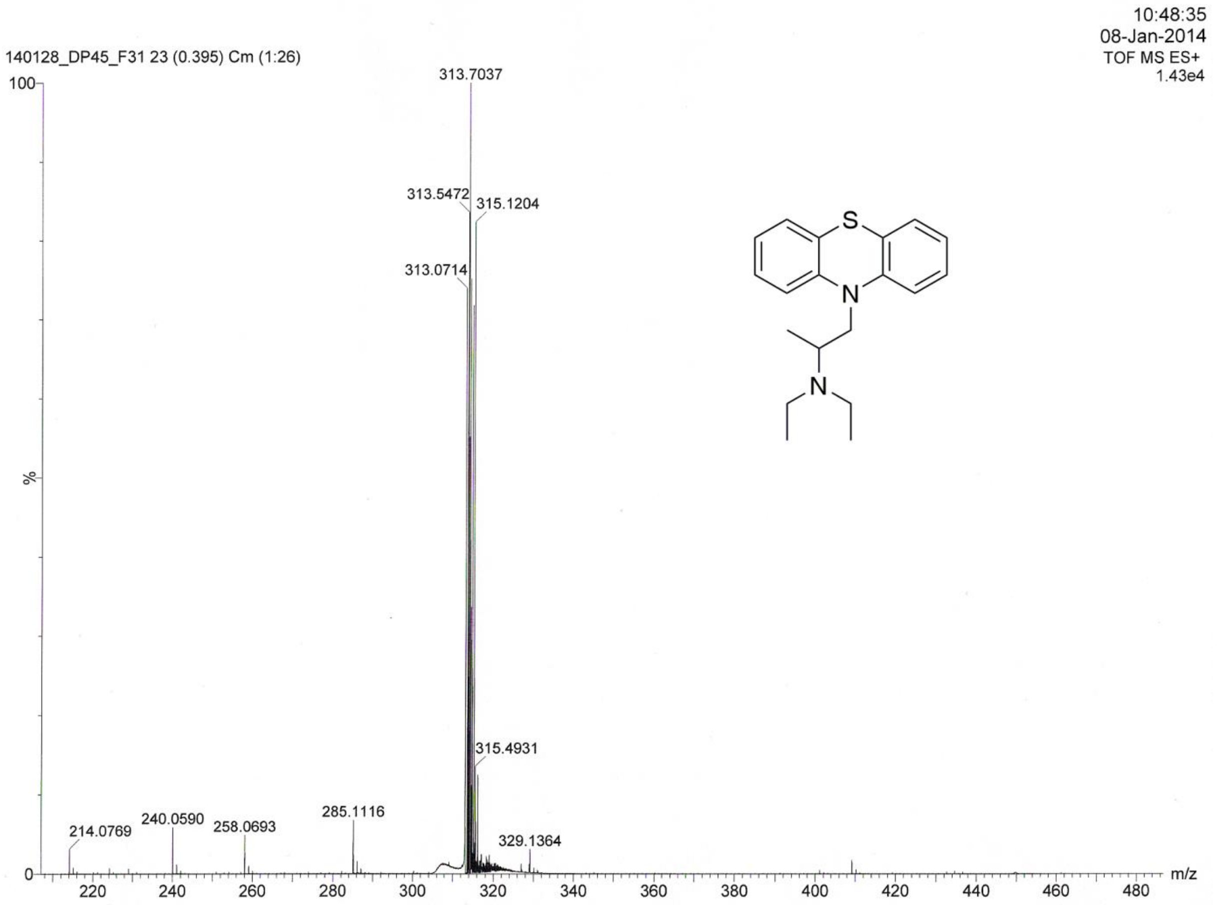

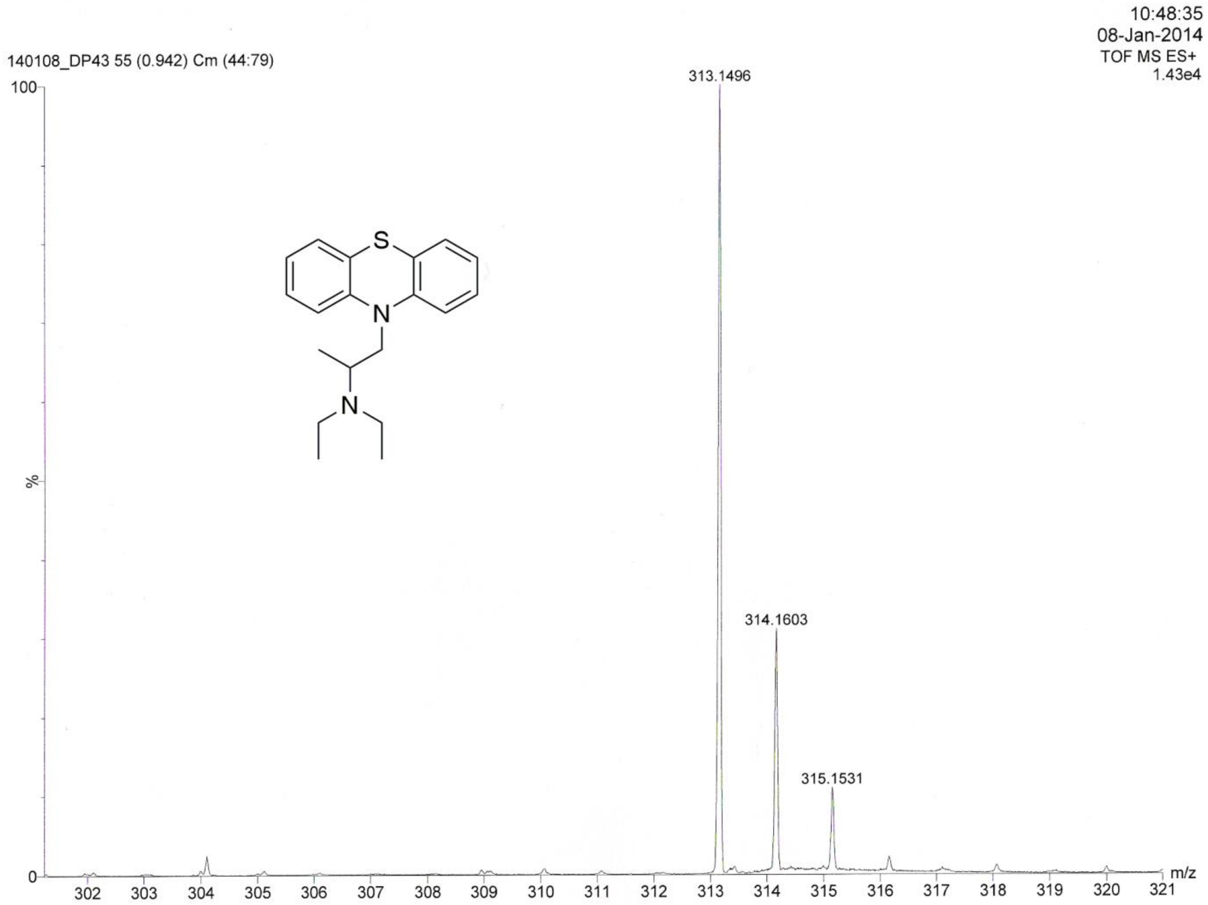
10:48:35
08-Jan-2014
TOF MS ES+
1.43e4
140108_DP43 55 (0.942) Cm (44:79)
313.1496
314.1603
315.1531
%
m/z
S
N

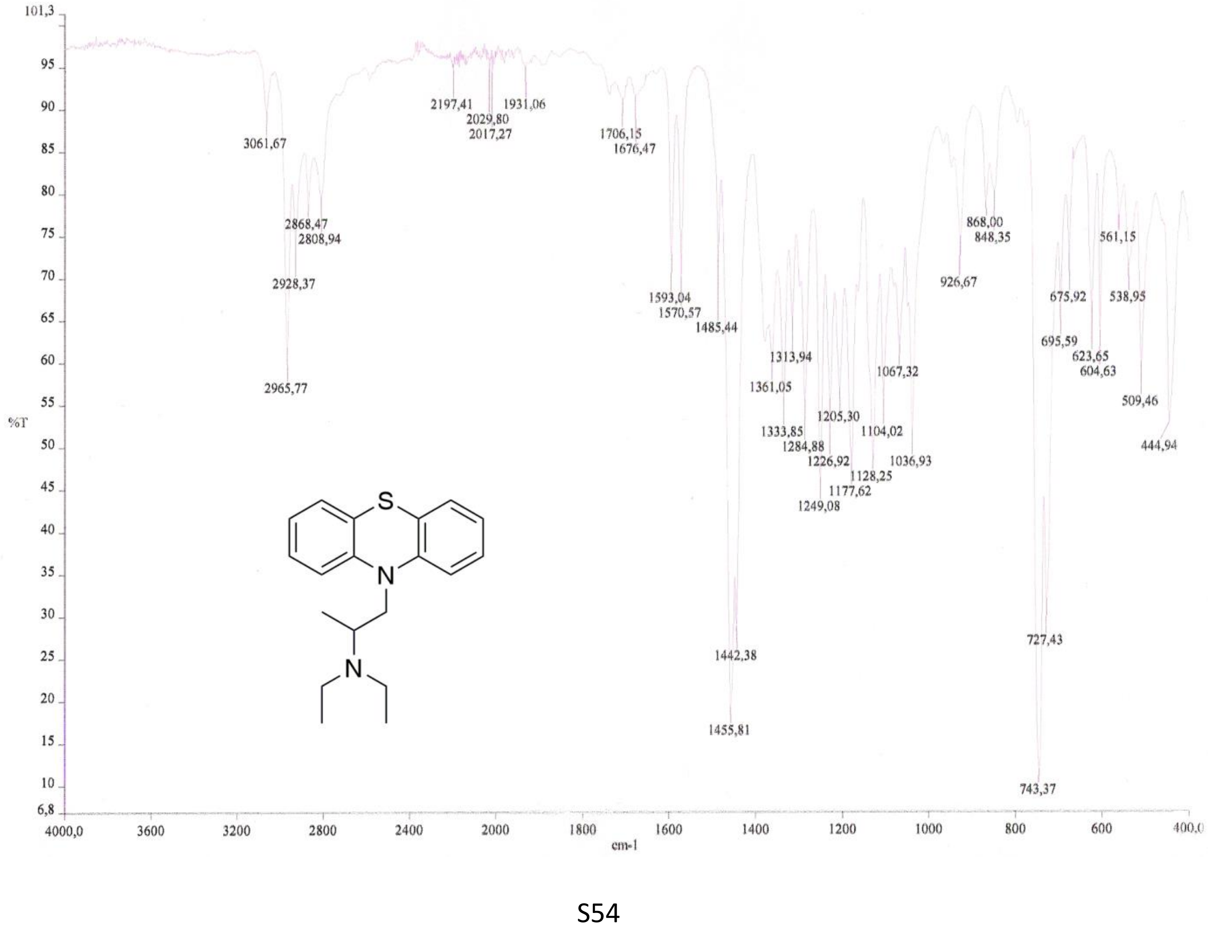
%T
cm-1
3061,67
2868,47
2808,94
2928,37
2965,77
2197,41
2029,80
2017,27
1931,06
1706,15
1676,47
1593,04
1570,57
1485,44
1442,38
1455,81
1313,94
1361,05
1333,85
1284,88
1226,92
1249,08
1205,30
1177,62
1128,25
1104,02
1067,32
1036,93
926,67
868,00
848,35
727,43
743,37
675,92
695,59
623,65
604,63
561,15
538,95
509,46
444,94
S
N

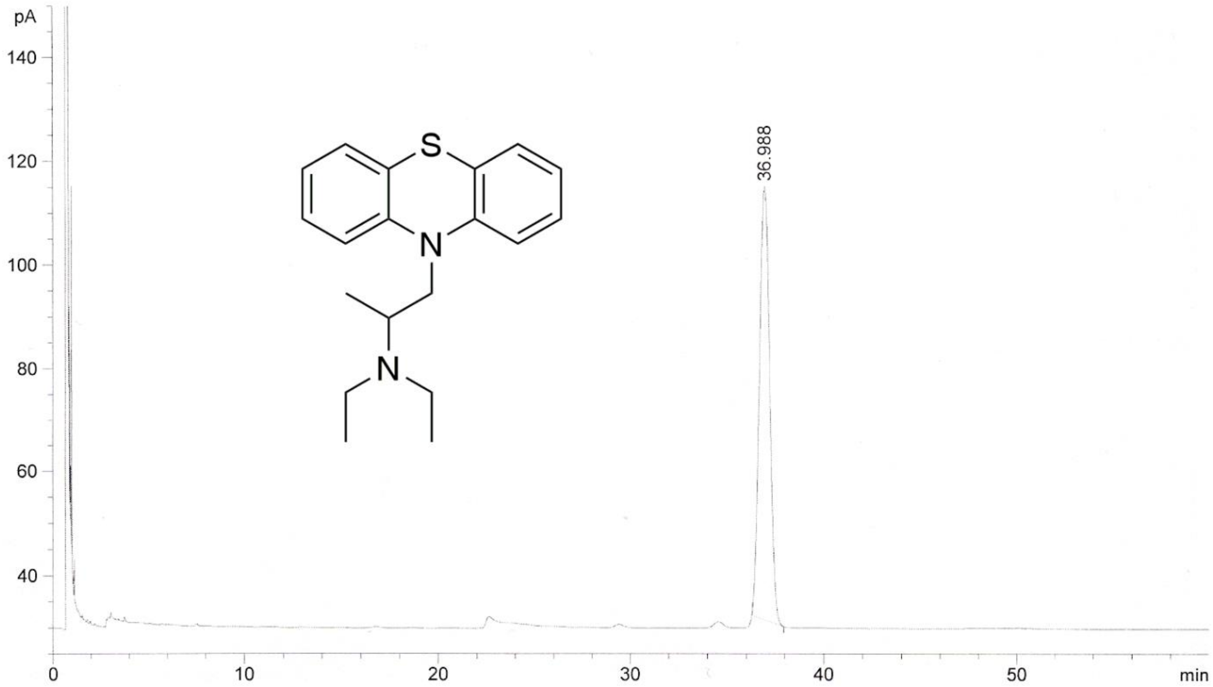

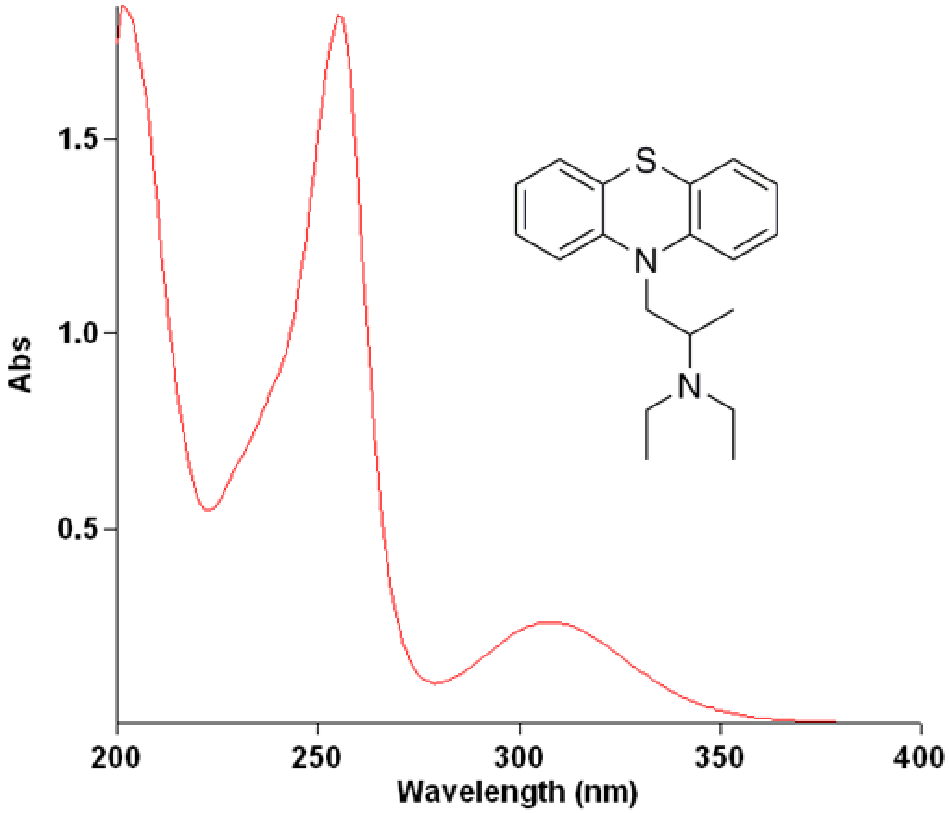

Wavelength (nm)	Abs
307.00	0.259
255.00	1.817
201.00	1.840

10-[(2*S*)-2-Methoxypropyl]-10*H*-phenothiazine (*S*)-16:

Dissolved in C_6D_6 and recorded on Varian Mercury 400BB spectrometer 400 MHz

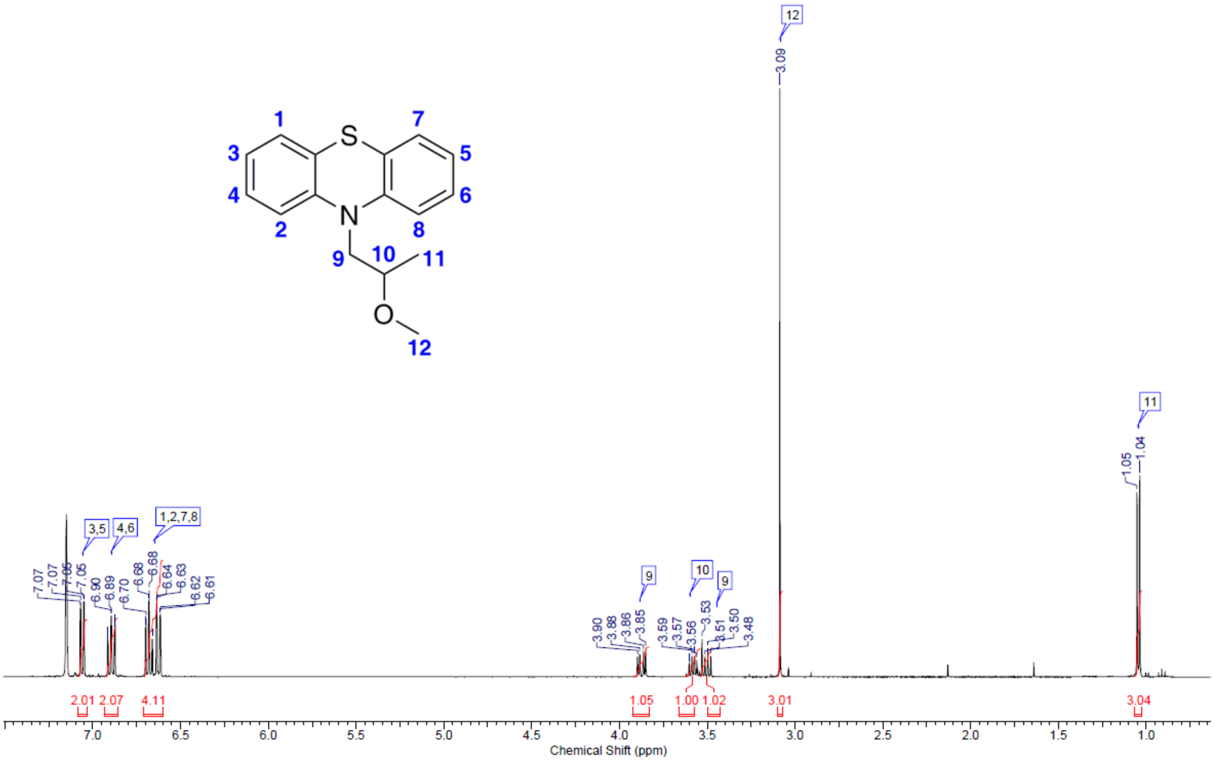

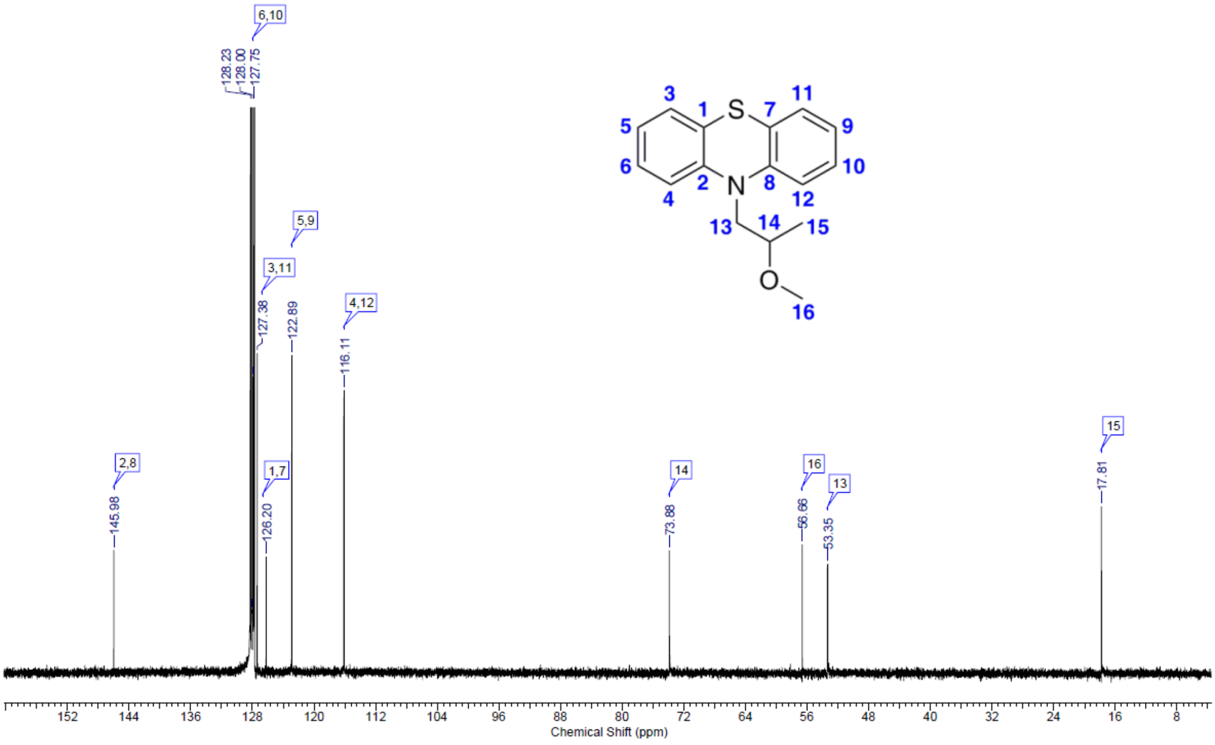

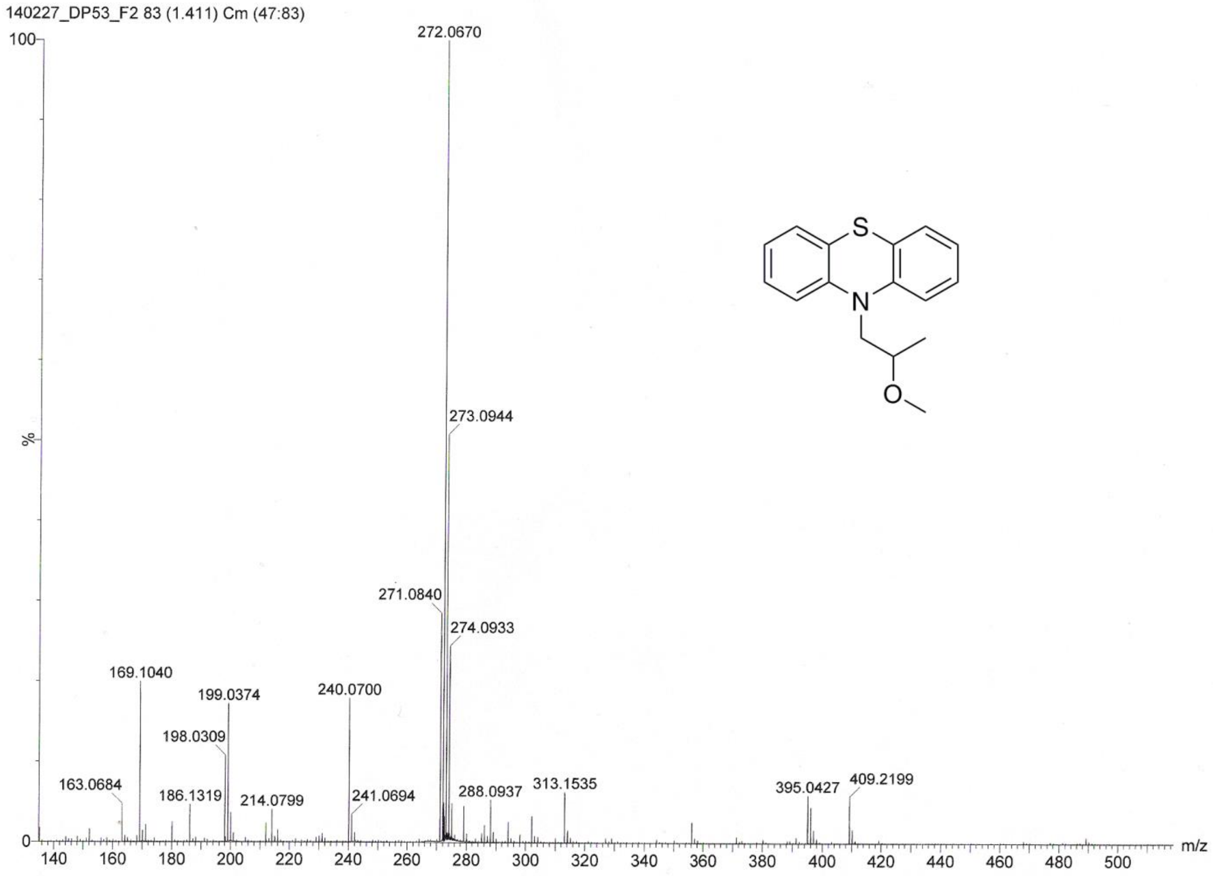

References

1. Bradley, D.; Williams, G.; Lawton, M. *J. Org. Chem.* **2010**, *75*, 8351-8354.

2. Clement, B.; Beckett, A. H. *Arch. Pharm.* **1981**, *314*, 716–722.

3. Dahlbom, R. *Acta Chem. Scand.* **1949**, *3*, 247–255.

4. *CRYSALIS^{PRO} Software system*, Agilent Technologies, Oxford, UK, **2013**.

5. Sheldrick, G. M. *Acta Cryst.* **2008**, *A64*, 112–122.

6. Dolomanov, O. V.; Bourhis, L. J.; Gildea, R. J.; Howard, J. A. K.; Puschmann, H. *J. Appl. Cryst*. **2009**, *42*, 339–341.

7. Flack, H. D. *Acta Cryst*. **1983**, *A39*, 876–881.

8. Parsons, S.; Flack, H. *Acta Cryst*. **2004**, *A60*, s61.

9. Spek, A. L. *Acta Cryst*. **2009**, *D65*, 148–155.

10. Hooft, R. W. W.; Straver, L . H.; Spek, A. L. *J. Appl. Cryst.* **2008**, *41*, 96–103.

11. Farrugia, L. J. *J. Appl. Crystallogr.* **1997**, *30*, 565.
